# Efficacy of lysostaphin-coated titanium plates on implant-associated MRSA osteitis in minipigs

**DOI:** 10.1007/s00068-024-02448-4

**Published:** 2024-01-24

**Authors:** Carina Jaekel, Ceylan D. Windolf, Dan Bieler, Lisa Oezel, Lars F. Seiler, Felix N. Lakomek, Christoph Beyersdorf, Jann Mertens, Andrea Steuwe, Joachim Windolf, Jan P. Grassmann

**Affiliations:** 1 Department of Orthopaedics and Trauma Surgery, Medical Faculty and University Hospital, Heinrich Heine University Düsseldorf, Moorenstraße 5, 40225 Düsseldorf, Germany; 2 Department of Trauma Surgery and Orthopedics, Reconstructive Surgery, Hand Surgery and Burn Medicine, German Armed Forces Central Hospital Koblenz, Koblenz, Germany; 3 Department of Trauma Surgery, Orthopaedics and Hand Surgery, Städtisches Klinikum Solingen, Solingen, Germany; 4 Department of Diagnostic and Interventional Radiology, Medical Faculty and University Hospital, Heinrich Heine University Düsseldorf, Düsseldorf, Germany; 5 Department of Trauma, Hand and Reconstructive Surgery, Klinikum Osnabrück GmbH, Osnabrück, Germany

**Keywords:** Plate-associated osteitis, Implant-associated infections, MRSA, Lysostaphin, Minipig, Biofilm

## Abstract

**Purpose:**

The growing incidence of implant-associated infections (IAIs) caused by biofilm-forming *Staphylococcus aureus* in combination with an increasing resistance to antibiotics requires new therapeutic strategies. Lysostaphin has been shown to eliminate this biofilm. Own studies confirm the effectiveness in a murine model. The current study characterizes the effects of lysostaphin-coated plates in an IAI minipig model.

**Methods:**

The femur of 30 minipigs was stabilized with a five-hole plate, a bone defect was created, and in 20 cases methicillin-resistant *Staphylococcus aureus* was applied. Ten animals served as control group. After 14 days, local debridement, lavage, and plate exchange (seven-hole plate) were performed. Ten of the infected minipigs received an uncoated plate and 10 a lysostaphin-coated plate. On day 84, the minipigs were again lavaged, followed by euthanasia. Bacterial load was quantified by colony-forming units (CFU). Immunological response was determined by neutrophils, as well as interleukins. Fracture healing was assessed radiologically.

**Results:**

CFU showed significant difference between infected minipigs with an uncoated plate and minipigs with a lysostaphin-coated plate (*p* = 0.0411). The infection-related excessive callus formation and calcification was significantly greater in the infected animals with an uncoated plate than in animals with a lysostaphin-coated plate (*p* = 0.0164/*p* = 0.0033). The analysis of polymorphonuclear neutrophils and interleukins did not reveal any pioneering findings.

**Conclusion:**

This study confirms the minipig model for examining IAI. Furthermore, coating of plates using lysostaphin could be a promising tool in the therapeutic strategies of IAI. Future studies should focus on coating technology of implants and on translation into a clinical model.

**Supplementary Information:**

The online version contains supplementary material available at 10.1007/s00068-024-02448-4.

## Introduction

The increasing number of fracture-related infection (FRI) is a major complication and challenging problem in trauma surgery. Especially the use of implants enhances the risk of infection, which is the main reason for treatment failure [1]. Implant-associated infection (IAI) in combination with growing resistance to conventional antibiotics requires novel therapeutic strategies. Staphylococci, with the leading species *Staphylococcus aureus* (SA) and *Staphylococcus epidermidis* (SE), are the most common causes of FRI, accounting for 30–60% of human cases [2–4]. SA’s ability to form a biofilm complicates the treatment. This biofilm can be defined as a structured microbial community of cells attached to a substrate and embedded in a matrix of extracellular polymeric substances that they previously produced [5, 6]. It protects bacteria from the penetration of immune cells, and prevents phagocytosis and reactive oxygen species (ROS) killing [6–10]. Experimental studies as well as clinical practice indicated clearly that in most cases, antibiotic therapy alone is not sufficient to eliminate the biofilm and that bacterial implants usually require surgical therapy [11]. Despite everything, it must be mentioned that each surgery to replace failed implants has a higher risk of recurrence, up to 30%, than that of the initial surgery [12]. Therapeutic strategies to eradicate the biofilm have made great research progress, such as studies on the surface modification of implants. However, these new treatments require further preclinical and clinical validation before they are widely accepted for therapeutic use [13]. Antimicrobial biomaterials currently represent one of the most promising therapeutic strategies [14, 15]. Therefore, the perfect implant surface would be one that reduces bacterial adhesion, confers effective sterilization, and does not compromise bone healing [16]. Great efforts have been made for coated implants, especially titanium plates, with antimicrobial agents to prevent IAIs [17, 18]. In 2014, our working group was able to show that an implant coating with lysostaphin prevents methicillin-sensible *Staphylococcus aureus* (MSSA) osteomyelitis in a murine model [19]. Lysostaphin is a 27-kDa extracellular antimicrobial endopeptidase produced by *Staphylococcus simulans biovar staphylolyticus* and was first discovered in the 1960s [20]. Lysostaphin destroys sessile bacteria in a biofilm and can also damage the extracellular biofilm matrix [21]. Moreover, the antibacterial potency of lysostaphin is well documented in animals and humans [20]. With regard to bony infection, the efficacy of lysostaphin has been tested under in vitro and in vivo animal models for the treatment of orthopedic infections [19, 22, 23]. Nonetheless, these studies were all carried out in mice. Examinations in large animal models (e.g., pig or sheep) are pending but are urgently needed to approach the clinical practice and pave the way for future human clinical trials. The most recent study of our working group shows the successful establishment of an IAI by methicillin-resistant *Staphylococcus aureus* (MRSA) in minipigs [24]. This was deliberately developed as a fundamental for investigating further therapeutic strategies in IAIs.

The aim of this study was to evaluate the effects of lysostaphin-coated plates in a standardized IAI minipig model.

## Materials and methods

### Animals and ethics statement

In this study, 30 2-year-old Aachener minipigs of heterogeneous sex (eight male, 22 female) with a mean weight of 64 kg were examined (animal facility of the Heinrich Heine University Düsseldorf; Zentrale Einrichtung für Tierforschung und wissenschaftliche Tierschutzaufgaben (ZETT), Germany). All animals were allowed to acclimate for 7 days after arrival before being included in the experiment. The animals were fed with commercial pig food, had free access to water, and were kept in separate stalls with a 12-h light/dark cycle. All animal procedures were carried out under local and national ethical guidelines and were approved by the regional ethical committee, Regional Office for Nature, Environment and Consumer Protection Nordrhein-Westfalen, Germany, with the ethical approval ID 84–02.04.2017.A181.

### Bacterial inoculum

The biofilm-forming MRSA strain ATCC 33592 was cultured in BactoTryptic Soy Broth overnight and then diluted 1:10. The mean inoculation colony-forming units (CFU) was 10^5^.

### Titanium plate coating

Titanium locking compression plates (LCP, DePuy Synthes, Raynham, USA) were coated with 1 mg/ml lysostaphin (ProSpec, East Brunswick, NJ, USA) in a poly(D,L)-lactide (PDLLA) matrix. PDLLA was dissolved in ethyl acetate (133 mg/ml) and mixed with lysostaphin to obtain a final concentration of 1 mg/ml. Sterile seven-hole LCPs (DePuy Synthes, Raynham, USA) were dipped in PDLLA-lysostaphin mixture and air-dried. This dip-drying step was repeated four times to obtain a multi-layer comprising PDLLA and lysostaphin. Titanium plates were used in animal experiments within 5 days.

### Low-grade acute osteitis model

**Fig. 1:**
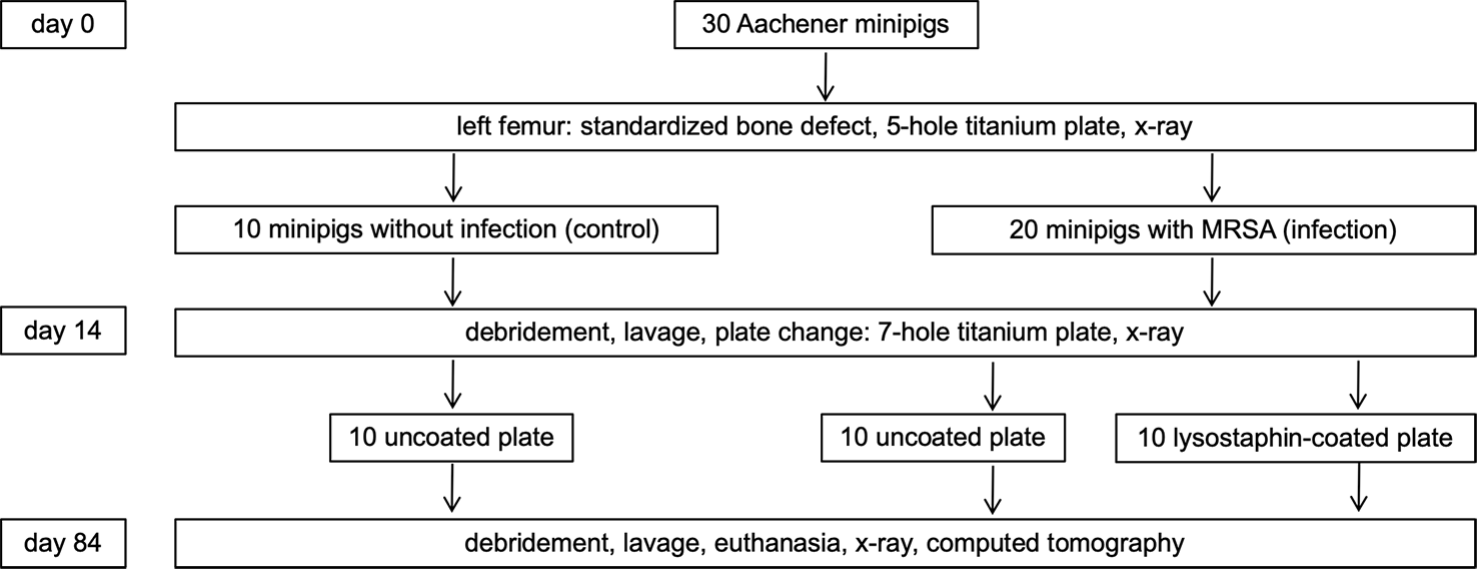
Experimental setup of the implant-associated minipig model

All surgeries were performed under aseptic conditions in the operating rooms of our local animal facility according to the standardized infection model previously established by our research group [24]. For premedication, we used ketamine 10 mg/kg i.m., azaperone 5 mg/kg i.m., diazepam 10–20 mg i.m., and atropine 0.5 mg i.m. The anesthesia was performed via thiopental 5 mg/kg i.v. and 2% isoflurane/oxygen mixture for the induction of anesthesia. The anesthesia was maintained with 1.3% isoflurane/oxygen mixture. Buprenorphine 0.3 mg i.v. was used as an analgesic. The dirt from the skin of the left hind leg was first removed with water and soap, and subsequently disinfected by swabbing with 70% alcohol. Before skin incision, we finally disinfected with Betaisodona. After skin incision in sterile, the fascia of the thigh muscle was exposed and then opened to dissect directly onto the bone. A standardized bone defect of 2.8 × 5 mm was created midway on the left femur using an LCP drill (DePuy Synthes, Raynham, USA). A five-hole LCP titanium plate 3.5 mm (DePuy Synthes, Raynham, USA) was modeled onto the bone. The plate was then implanted laterally on the femur with four locking self-tapping screws (Stardrive®, 3.5 mm, titanium, DePuy Synthes Raynham, USA). In 20 animals, 5 × 5 µl MRSA (ATCC 33592) with a mean CFU of 10^5^ was placed on the plate to dry. Ten animals were given a sterile LCP titanium plate (control group). For postoperative pain management, all animals received oral metamizole 20 mg/kg three times daily for 3 days and buprenorphine 0.3 mg i.m. for the night. They also received oral meloxicam 0.4 mg/kg once daily for 5 days. Systemic antibiotic therapy with enrofloxacin 2.5 mg/kg was performed from the first to the third postoperative day. On day 14, plate change from five-hole to seven-hole LCP titanium plate 3.5 mm (DePuy Synthes Raynham, USA) was performed. The five-hole LCP titanium plate was removed, and lavage and debridement were conducted. Lavage and blood samples were taken. Ten of the infected animals and the 10 control animals received a sterile seven-hole LCP titanium plate. The other 10 infected animals received a seven-hole LCP titanium plate coated with lysostaphin. On day 84, the animals were anesthetized again. Lavage and blood samples were collected, after which the animals were euthanized with an overdose of thiopental. Figure 1 shows the experimental setup.

On day 0, all minipigs received a standardized bone defect midway on the left femur. A five-hole titanium plate was modeled onto the bone. In 20 animals, methicillin-resistant *Staphylococcus aureus* (MRSA) was placed on the plate. On day 14, lavage and debridement were performed in all mini-pigs. Ten infected and 10 control minipigs received a sterile seven-hole titanium plate. The other 10 infected minipigs received a seven-hole titanium plate coated with lysostaphin. On day 84, lavage and blood samples were collected again, the minipigs were euthanized, and computed tomography examinations were performed.

### Counts of colony-forming units (CFU)

Level of CFU was determined in the lavages on days 14 and 84. Lavages were obtained during debridement. Therefore, 200 μl of the lavage fluid was serially diluted in phosphate-buffered saline (PBS). Four replicates of 10 μl of each dilution were applied to Columbia agar plates containing 5% sheep blood. The plates were incubated at 37 °C for 24 h. Colonies were then counted. Results are expressed as CFU/ml of lavage fluid (quadruplicate preparation).

### Radiographic and computer tomographic analysis

The plate position (day 0) and bone healing (day 14 and 84) were controlled radiologically with a standard digital X-ray machine. After sacrificing the animals, the femurs were removed and fixed in 4% formalin.

Image acquisition was performed on a single-source computed tomography (CT) scanner (Somatom Definition Edge, Siemens Healthineers, Forchheim, Germany). A high-resolution CT scan was performed with a detector collimation of 16 × 0.3 mm at 120 kV_p_. High-resolution multiplanar reformations were reconstructed with a slice thickness of 0.4 mm and a bone kernel in axial, sagittal, and coronal directions. The sizes of all drill holes as well as the total volume of the bone and the callus were measured in the image analysis software syngo.via (Siemens Healthineers, Forchheim, Germany). Furthermore, 3D reconstructions of each bone were made using the local picture archive and communication system (PACS, Sectra IDS 7, Linkoping, Sweden).

### Analysis of local and systemic immune responses

For local and systemic immune response, polymorphonuclear neutrophils (PMN) were measured as a percentage of total leukocyte count in lavage and blood using flow cytometry (FACSLytic, BD Pharming™, Heidelberg, Germany) with CD172a (PE Mouse Anti-Pig Monocyte/Granulocyte, BD Pharmingen™, San José, USA) and 6D10 (FITC Granulocytes Monoclonal Antibody, Invitrogen Thermo Fisher Scientific, Waltham, USA) antibodies. Samples were tested in duplicate.

### Quantification of IL-6 and IL-17 by ELISA

Interleukin (IL)-6 and IL-17 levels in lavage and serum were determined using a commercially available immunokit (Porcine IL-6 Quantine® ELISA, R&D Systems®, Porcine IL-17, Invitrogen, Thermo Fisher Scientific, Waltham, USA) in a microplate reader (VICTOR X3 Plate Reader, PerkinElmer LAS, Rodgau, Germany). The mean of minimum detectable dose (MDD) of IL-6 was 2.03 pg/ml and MDD of IL-17 14 pg/ml. Samples were tested in duplicate.

### Alkaline phosphatase and bone-specific alkaline phosphatase level

The non-specific alkaline phosphatase (ALP Assay Kit, Abnova GmbH, Heidelberg, Germany) and the bone-specific alkaline phosphatase (Porcine bALP ELISA Kit, MyBioSource, San Diego, USA) were measured in lavage and serum at a wavelength of 405 nm after 0 and 4 min (Victor X3, Plate Reader, PerkinElmer LAS, Rodgau, Germany). Samples were tested in duplicate.

### Amino-terminal propeptide of type I collagen

Amino-terminal propeptide of type I collagen (PINP) concentration (Porcine procollagen 1N-terminal peptide ELISA Kit, BT Lab, Birmingham, UK) was measured in lavage and serum in a microplate reader (Victor X3, Plate Reader, PerkinElmer LAS, Rodgau, Germany) according to the manufacturer’s instructions. Samples were tested in duplicate.

### Statistical methods

All data are expressed as median and scatter dots. Omnibus normality test by D’Agostino and Pearson’s. Data were tested for statistical significance with two-tailed Student’s *t*-test and Mann–Whitney *U* test using GraphPad Prism5 (GraphPad Software, San Diego, USA): *p* values ≤ 0.05 were considered as significant.

## Results

### Clinical observations

All animals tolerated the surgical procedures very well and returned to their normal activities after anesthesia, eating and drinking independently. One animal in the group with lysostaphin-coated plates had a local multi-infection with *Escherichia coli*, *Streptococcus pyogenes*, *Streptococcus suis*, and *Staphylococcus* spp., so we excluded the minipig from the experiment. All other 29 animals survived throughout the study period without any plate breakage, fracture, or other adverse events. We could euthanize all minipigs according to the described study protocol.

### Low-grade acute osteitis model

**Fig. 2:**
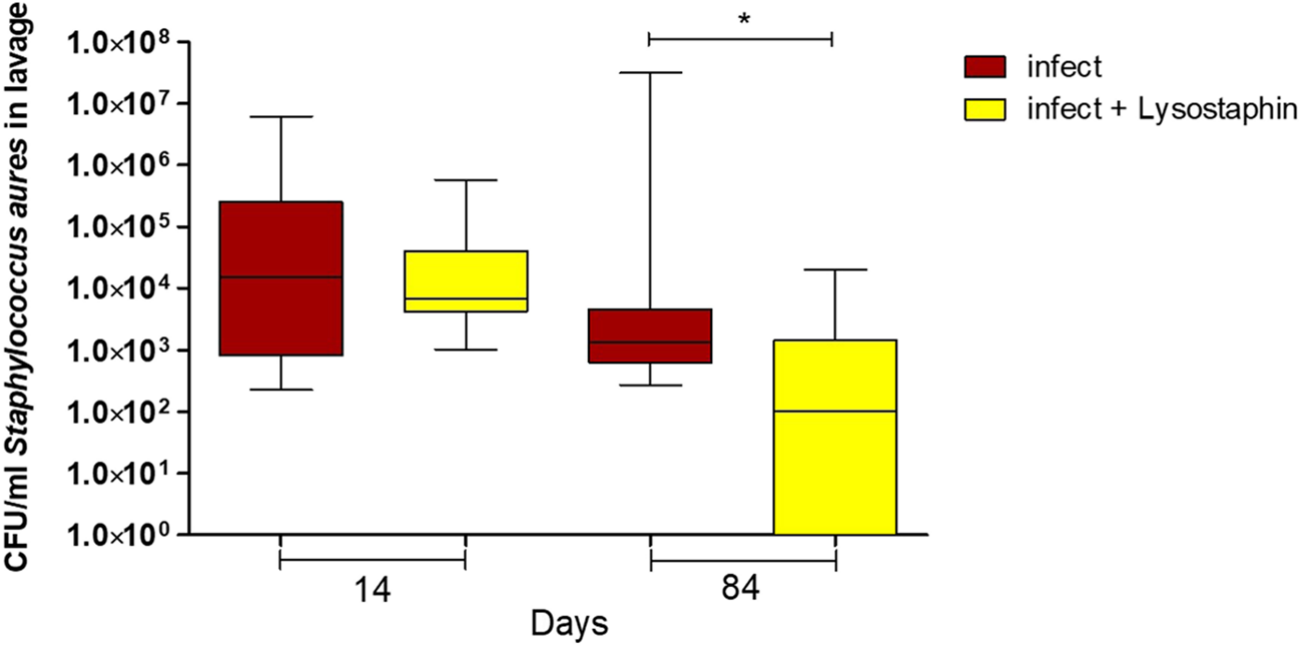
Bacterial load of methicillin-resistant *Staphylococcus aureus* (MRSA) in lavage on days 14 and 84. Colony-forming unit (CFU) levels in lavage showed a significant reduction in the lysostaphin-coated plate group on day 84 (*p* = 0.0411)

The bacterial load in the wound was determined by CFU on days 14 and 84 with an average inoculation CFU of 10^5^. Lavage showed significantly lower CFU on day 84 in infected animals with a lysostaphin-coated plate compared to infected animals with an uncoated plate (*p* = 0.0411); see Fig. 2. In three animals with a lysostaphin-coated plate, MRSA could no longer be detected on day 84.

### Bone healing

**Fig. 3:**
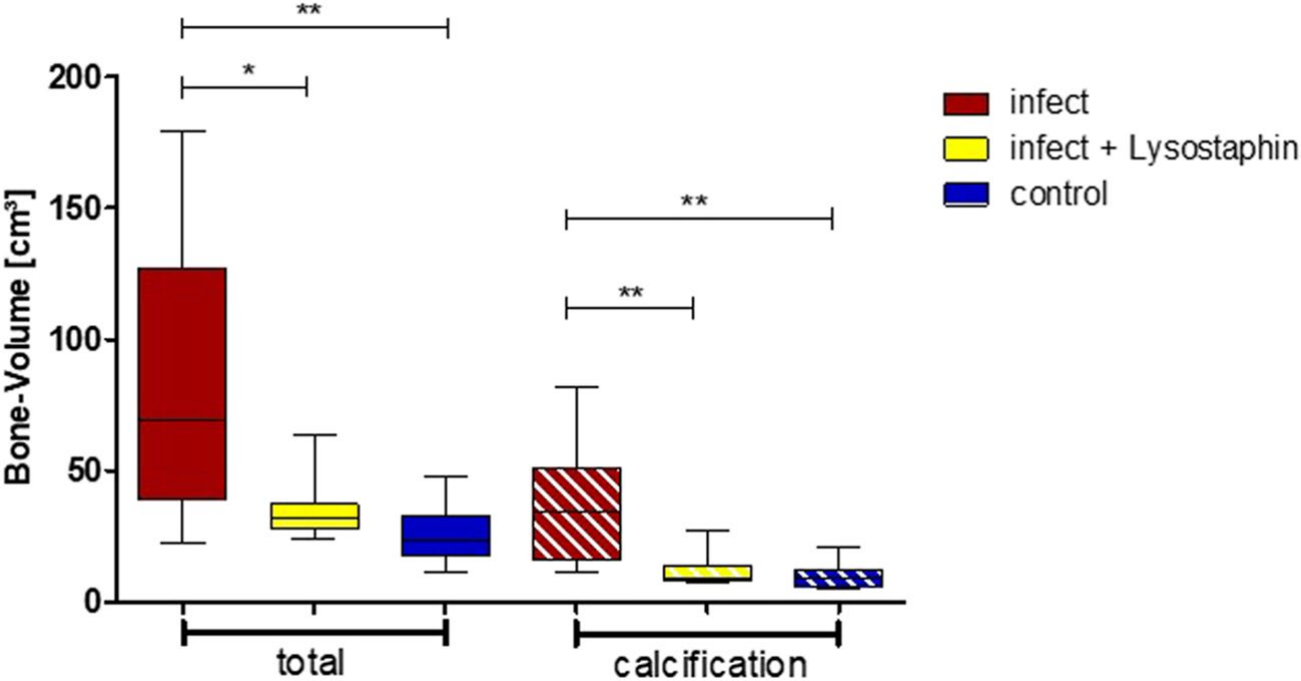
Bone volume and calcified bone volume of the femur on day 84. Infection group with uncoated plates showed a significant higher callus formation, measured by the total bone volume, compared to the control group (*p* = 0.0024). For the infection group with lysostaphin-coated plates, a significant regulated callus formation could be observed (*p* = 0.0164). Similar results can also be seen for the calcified portion of the bone volume (*p* = 0.0033, *p* = 0.0031)

**Fig. 4:**
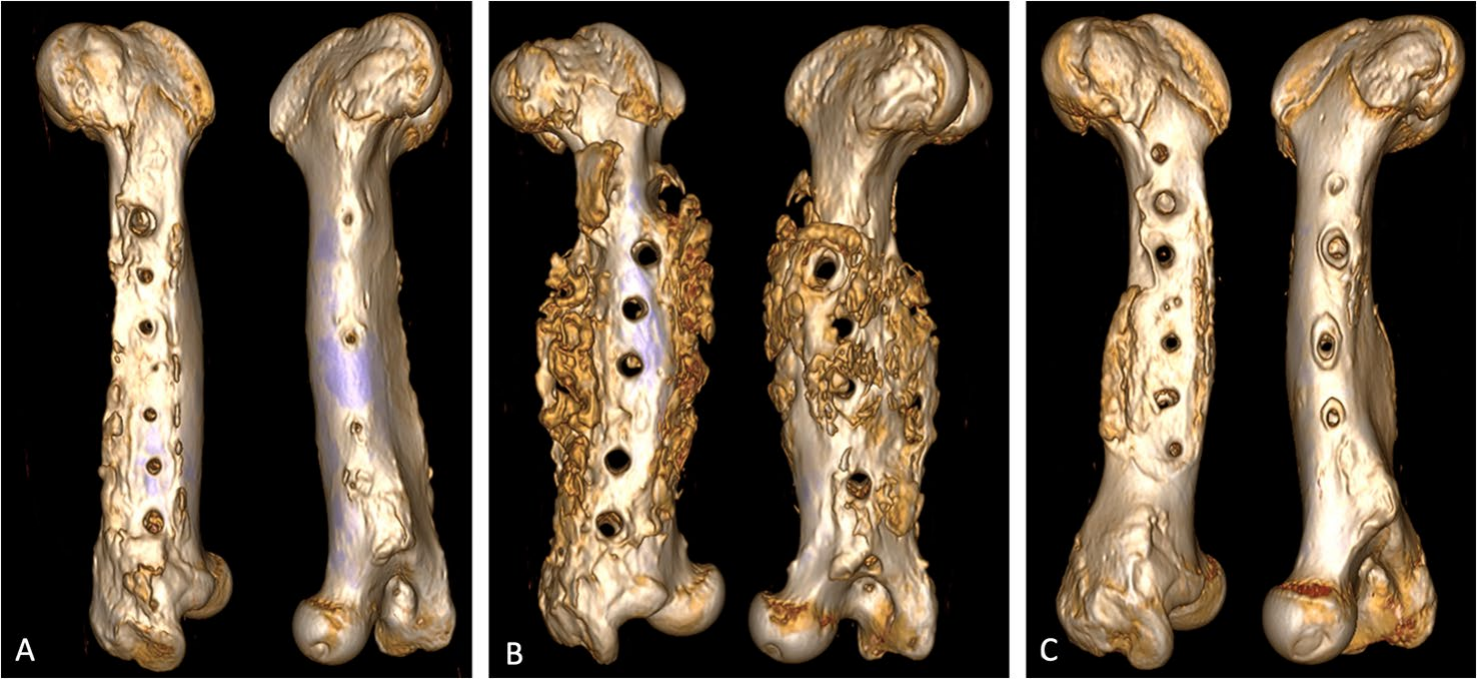
Examples of 3D imaging of the femur. 3D imaging with computed tomography (CT) of the femur on day 84. **A** Left femur of a minipig from the control group. Minimal to no callus tissue visible with round and symmetrical drill holes. **B** Left femur of a minipig from the infected group with uncoated plates. A considerable amount of callus tissue visible with asymmetrical and large drill holes in line with osteolysis. **C** Left femur of a minipig from the infected group with lysostaphin-coated plates. Minimal callus tissue visible with slightly asymmetrical and slightly enlarged drill holes in line with osteolysis

**Fig. 5:**
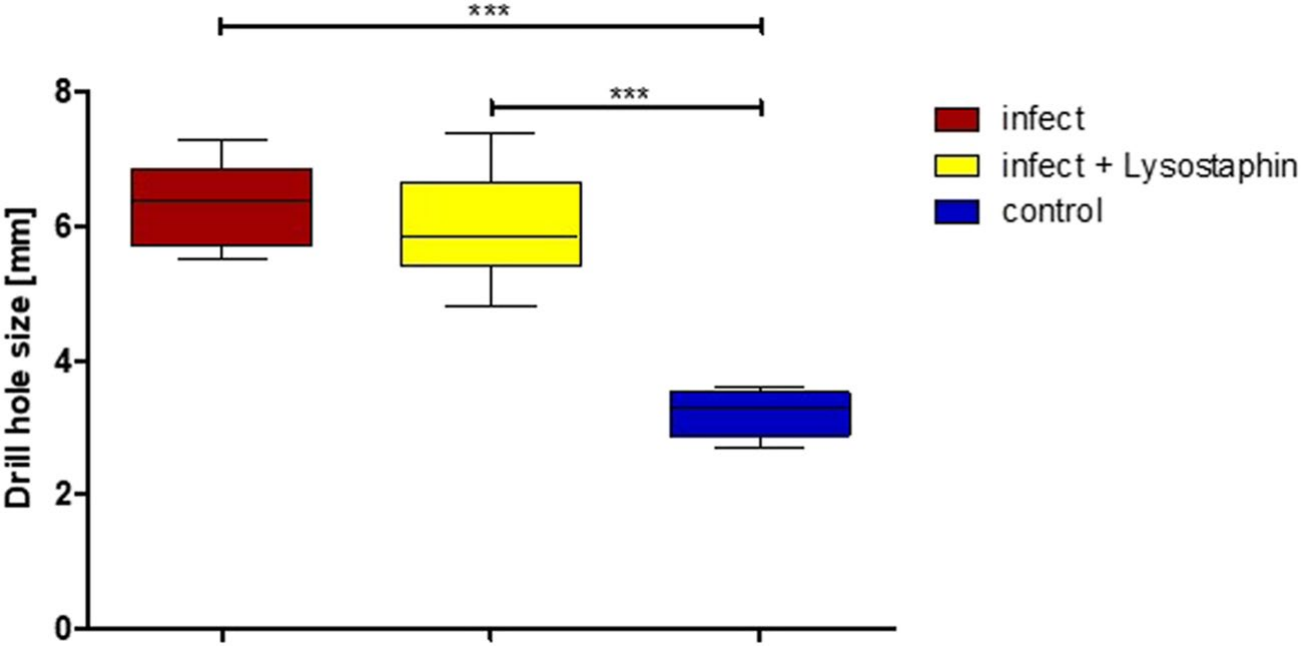
Size of drill holes of the femur. Significant increased size of the drill holes was demonstrated for both the infection group with uncoated plates and the infection group with lysostaphin-coated plates compared to the control group (*p* < 0.0001)

Bone healing was checked radiologically in all animals on days 0, 14, and 84. In the infected animals without a lysostaphin plate, excessive callus formation, measured by total bone volume and calcified bone volume, was observed by 3D CT on day 84 (Figs. 3 and 4, Sup 1). This could be inhibited in the infected animals with a lysostaphin-coated plate (*p* = 0.0164, *p* = 0.0033). Furthermore, the degree of osteolysis of the screw holes was determined. Here, there was no significant difference in the infected animals with and without lysostaphin-coated plates (*p* = 0.2673). However, we observed a trend towards a positive effect by a lysostaphin-coated plate (Fig. 5). The examined biochemical markers of bone metabolism ALP and PINP showed no significant differences in the infected animals with and without lysostaphin-coated plates (Sup 2 and 3). Furthermore, no significant difference between ALP and bone-specific ALP (bALP) could be demonstrated.

### Immune response

**Fig. 6:**
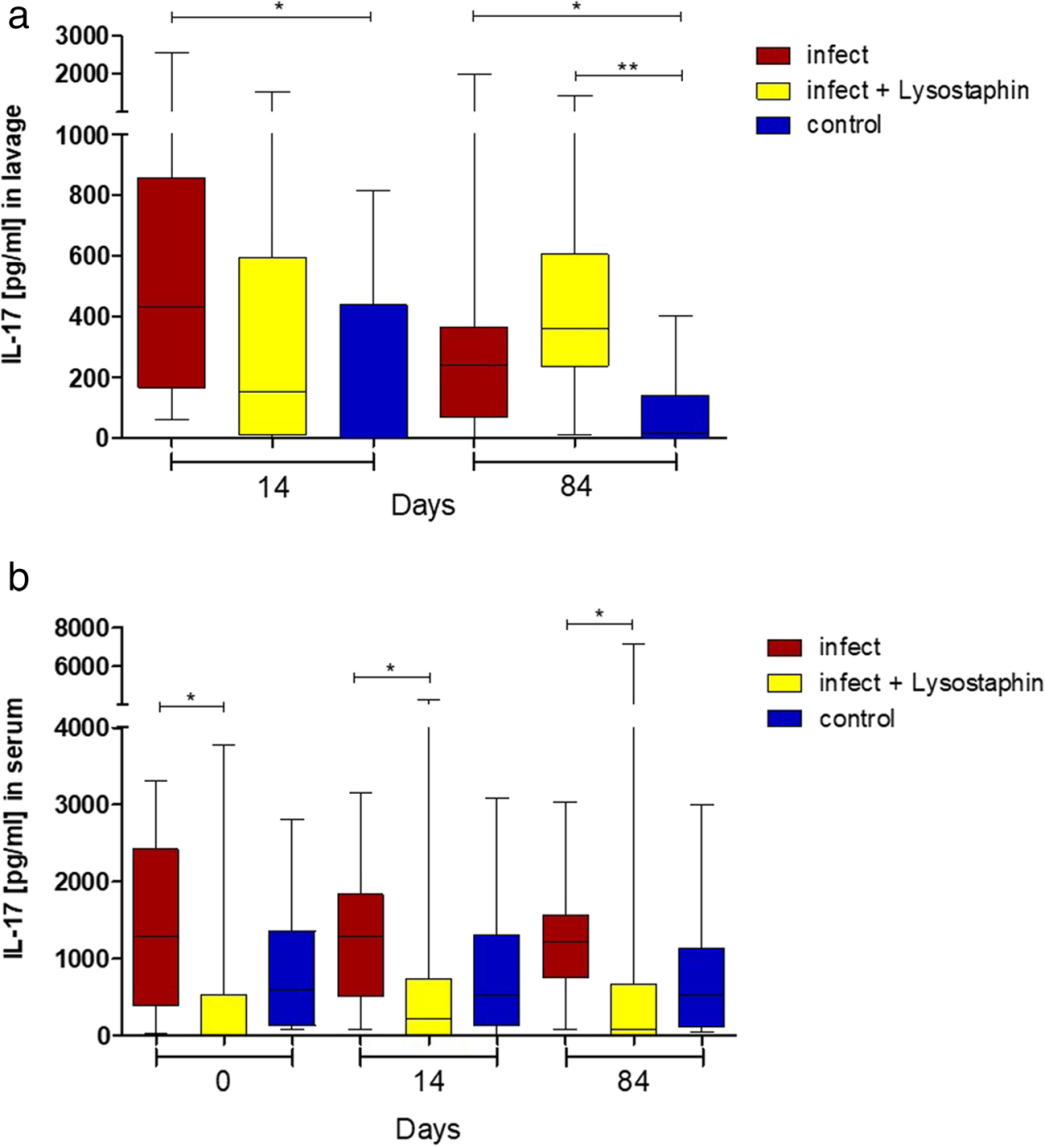
Interleukin-17 in serum (**a**) and lavage (**b**). In serum, we could detect a significant difference between uncoated infection group versus lysostaphin-coated group on day 0 (*p* = 0.0115), day 14 (*p* = 0.0355), and day 84 (*p* = 0.0172). There was a significant difference of IL-17 in lavage for lysostaphin-coated plates compared to control (*p* = 0.0021) on day 84

Figure 6 shows a significant difference for IL-17 in serum of minipigs with lysostaphin-coated plates as in the minipigs with uncoated plates on days 0, 14, and 84. Furthermore, there was significant difference of IL-17 in lavage for lysostaphin-coated plates compared to control (*p* = 0.0021) on day 84.

**Fig. 7:**
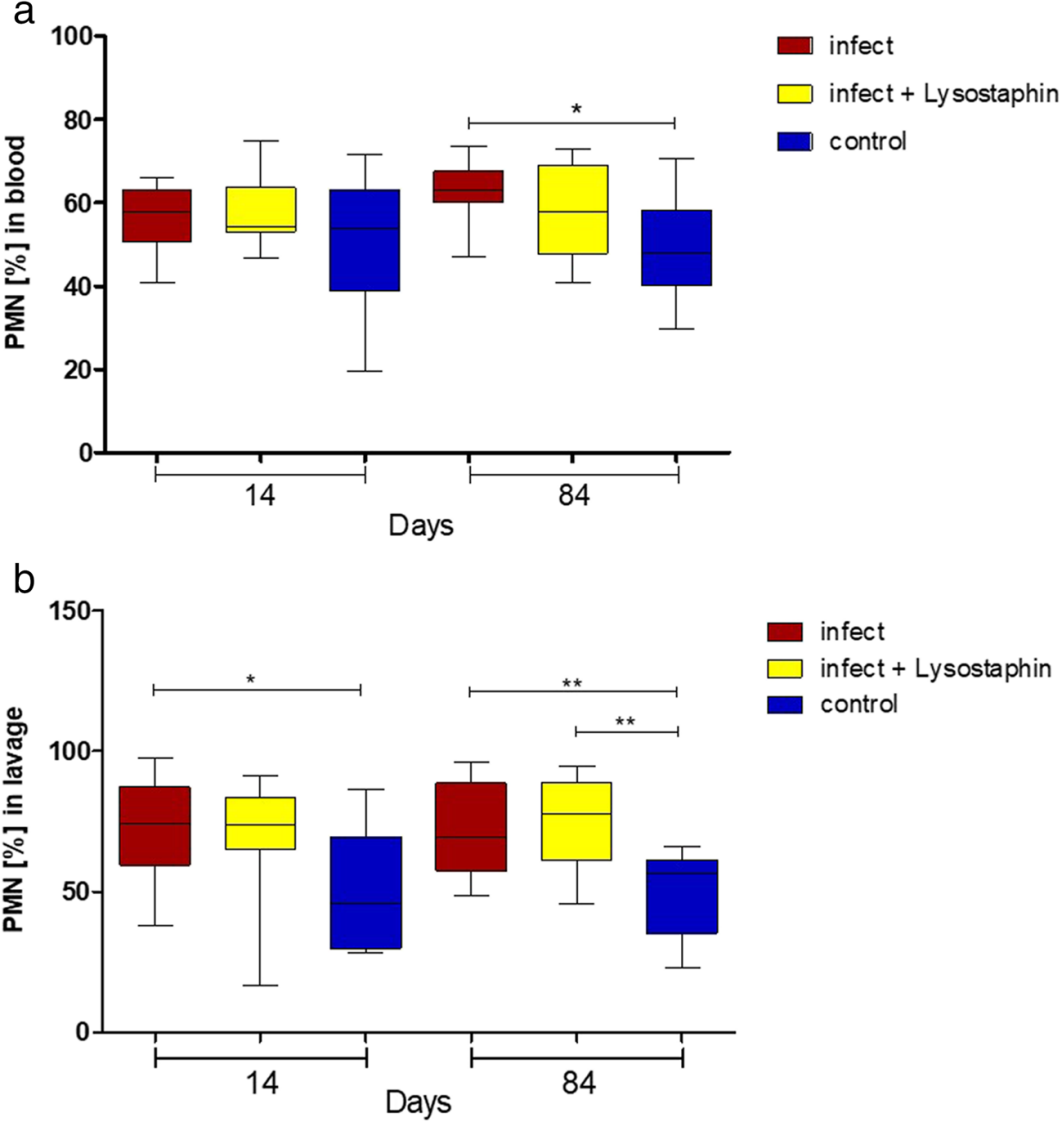
Neutrophil granulocytes in serum (**a**) and lavage (**b**). There were no significant differences of polymorphonuclear leukocytes (PMN) in blood and lavage for lysostaphin-coated plates compared to uncoated plates on days 14 and 84, although there were significant differences compared to the control group (blood day 84: *p* = 0.0224, lavage day 14: *p* = 0.0220, lavage day 84: *p* = 0.0096, *p* = 0.0044)

Further local and systemic immune response analysis showed no significant differences between the infected animals with and without lysostaphin-coated plate for PMN (Fig. 7) as well as for IL-6 (Sup 4). Immune response only showed differences between infection and control groups, and can therefore confirm our osteitis model.

## Discussion

Implant-associated infections (IAI) remain one of the greatest challenges in orthopedic and trauma surgery, despite major advances that have been achieved regarding their diagnosis and treatment. Currently, the most important treatment principles for IAI include surgical debridement, implant handling, long-term systemic antibiotics, reconstruction defects of bone and soft tissues, and functional recovery [25]. Fundamental research in vitro and in vivo should be performed to uncover the pathogenesis of this disorder more comprehensively. Here, we can confirm the established, standardized minipig model for examining IAI on the one hand, and the antimicrobial coating using lysostaphin as a possible therapeutic agent in IAI on the other hand.

Key problem of implant infections is the lack of effective therapies. Alternative strategies with antimicrobial enzymes such as lysostaphin could provide new options by killing planktonic and quiescent bacteria as well as bacteria growing in a biofilm [26]. In this study, the implanted titanium plates were dipped in a PDLLA-lysostaphin mixture and air-dried. This dip-drying step was repeated four times to obtain a multi-layer comprising PDLLA and lysostaphin. Other biomaterial strategies to deliver active lysostaphin have primarily focused on surface functionalization, by either passive adsorption [27, 28] or covalent tethering [29, 30]. Johnson et al*.* postulate that all these techniques, including our own, are effective ways to reduce bacteria at the material surface but may not be practical for settings where infection is already established, or not localized to a material surface [22]. They demonstrate that lysostaphin-delivering hydrogels have greater antibiofilm activity compared with soluble lysostaphin, even in a FRI model. However, these results have so far only been demonstrated for a small animal model (murine model) and are only been tested in an MSSA (UMAS-1) strain [22, 23]. Perhaps, lysostaphin combinations of implant coating and hydrogel application would be promising. To increase lysostaphin stability and retention times at the site of administration, Xue et al*.* analyzed the lysostaphin delivery using hydroxyapatite/chitosan composite bone cement [31]. Summarized, their study suggested that the lysostaphin-loaded self-setting injectable bone cement released the enzyme in a controlled and effective way. Furthermore, the enzymatic activity was well preserved during the setting and release process. To date, however, these experiments have only been carried out in the subcutaneous tissue of mice, and results in an IAI model are still pending.

Lysostaphin is not the only coating that has been tested in the treatment of patients with IAI from/resulting from medical devices [32]. In the last decade, several studies have investigated the ability of implant surface modifications to minimize bacterial adhesion, inhibit biofilm formation, and provide effective bacterial killing to protect implanted biomaterials [33]. Thereby, we can distinguish (1) antibacterial coatings (passive or active) and (2) modification of the surface of medical implants (physical or chemical) [34]. The lack of a common language and a common outcome set, as well as a large discrepancy between proposed and clinically implemented strategies, complicates evaluation of individual procedures and their comparison. Jeyaraman et al*.* summarize the current study situation on silver nanoparticles (AgNP) technology and conclude that AgNP has the potential to reduce implant-related infections. Yet, further studies and regulatory guidelines to avoid toxicity in clinical applications are pending.

Current therapeutic concepts require the implant removal in the treatment of osteitis, especially due to the formation of biofilm [35]. However, if the fracture has not healed, an implant replacement should be performed at least. To our knowledge, this is the first animal implant-associated osteitis model, which allows a plate replacement and thus forms a good foundation for future studies.

This study has several limitations. First, in contrast to our previously established mouse model, we also identified a significant number of foreign bacteria in one pig (*Escherichia coli*, *Streptococcus pyogenes*, *Streptococcus suis*, *Staphylococcus* spp.) when analyzing the bacterial load. Unfortunately, this cannot be avoided due to the pigs’ natural behavior, although we performed a standardized and careful disinfection of the pig skin before the respective operations. Finally, we excluded the pig in which this high number of foreign bacteria was detected from the analyses.

Secondly, the analysis of PMN, IL-6, and IL-17 did not reveal any pioneering findings. This could certainly be related to the chosen measurement times. For animal welfare reasons, the first lavage and blood samples were taken as part of the revision surgery on day 14, relatively late after infection induction. This led to a lower immune response than we expected, even in the infected animals. Compared to our own mouse model and the study of Johnson (both IL determinations were carried out on day 7), the measured IL levels in the present study were only half as high, even in the infected animals. Future studies should provide early assessments (e.g., days 1–6) of immunological parameters.

Thirdly, the analysis of the drill holes revealed no significant difference between the uncoated and the lysostaphin-coated plates, although CT showed significant differences among bone volume and mineralization. This could be attributed to the lysostaphin coating in the area of the screws. Stability of the PDLLA-lysostaphin-coating has not been evaluated so far. During inserting of the self-tapping screws, the coating could be lost due to the forces acting there. Finally, we cannot say with certainty what concentration was present after implantation of the osteosynthesis. Further studies to investigate surface adhesion, particularly in the area of screws, are pending, but planned.

Lastly, animal experiments cannot fully simulate the complex immunological and biomechanical environments of the human body, which limits the conclusions of the study. Going forward into a clinical setting will be challenging.

## Conclusion

Based on the presented in vivo study, we can confirm our minipig model for the investigation of implant-associated bone infections, especially when an exchange of the implant should be performed. This enables further advance approaches and therapeutic options to be evaluated using this model.

Moreover, we demonstrate that lysostaphin coating prevents the development of an infection in spite of a bacterial contamination of MRSA and the presence of an implant, normally leading to progressive infection due to biofilm formation. From the clinical perspective, an antimicrobial coating of the osteosynthesis material would be desirable in high-risk cases (e.g., in the treatment of open fractures or in elderly patients with compromised tissue) or even after the infection has been clinically resolved. Future studies should focus on the development of effective coating strategies and the establishment of clinical model.

### Supplementary Information

Below is the link to the electronic supplementary material.

ESM 1 (PDF 4043 kb)

ESM 2 (PNG 212 kb)

High resolution image (TIF 101 kb)

ESM 3 (PNG 179 kb)

High resolution image (TIF 86 kb)

ESM 4 (PNG 180 kb)

High resolution image (TIF 85 kb)

## Data Availability

No datasets were generated or analysed during the current study.
