# Supplementary material for: Efficacy of lysostaphin-coated titanium plates on implant-associated MRSA osteitis in minipigs
Source: Eur J Trauma Emerg Surg. 2024 Jan 24;50(3):887–95. doi: 10.1007/s00068-024-02448-4 (PMC11249774; doi:10.1007/s00068-024-02448-4)

Control Group / No Infection
Pig No. 6

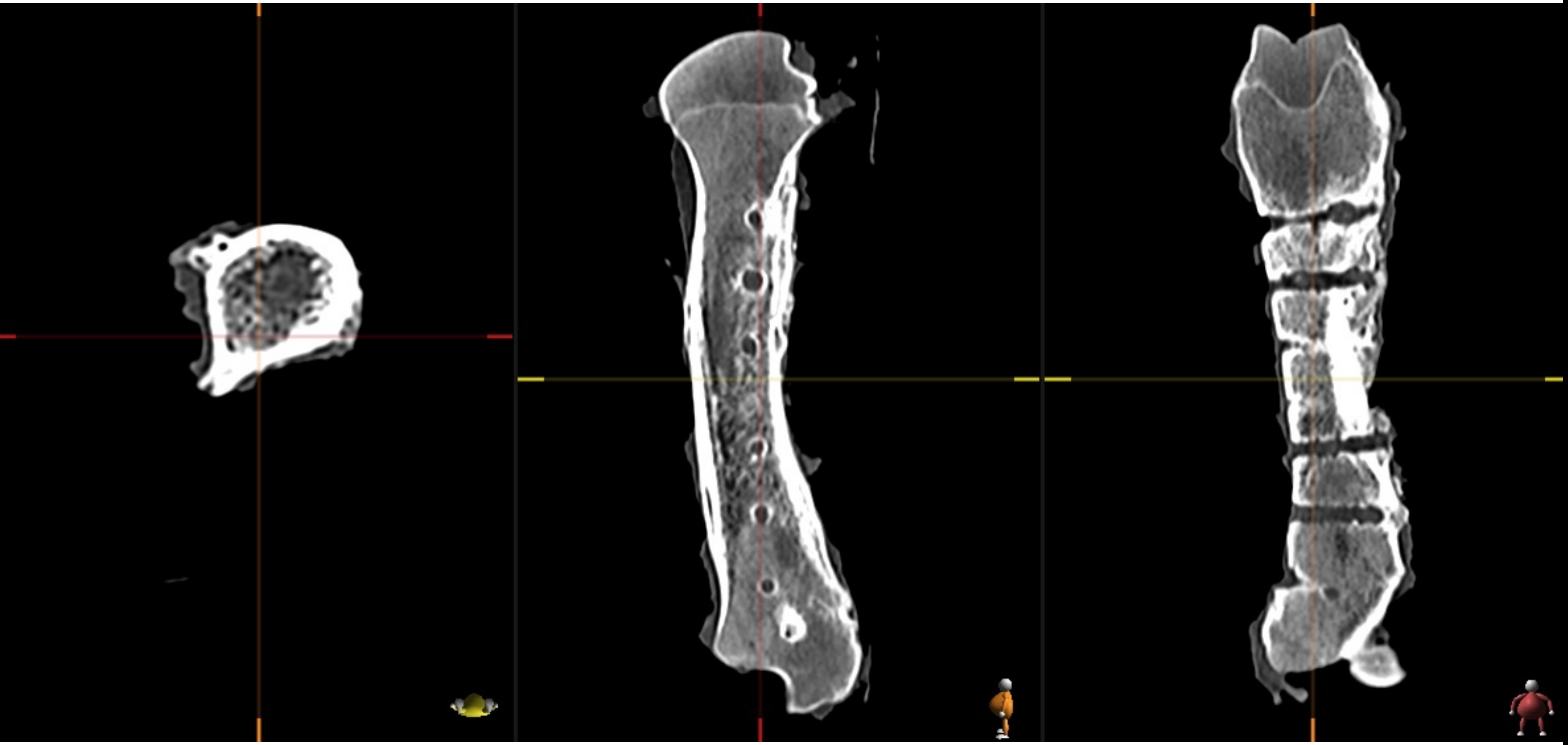

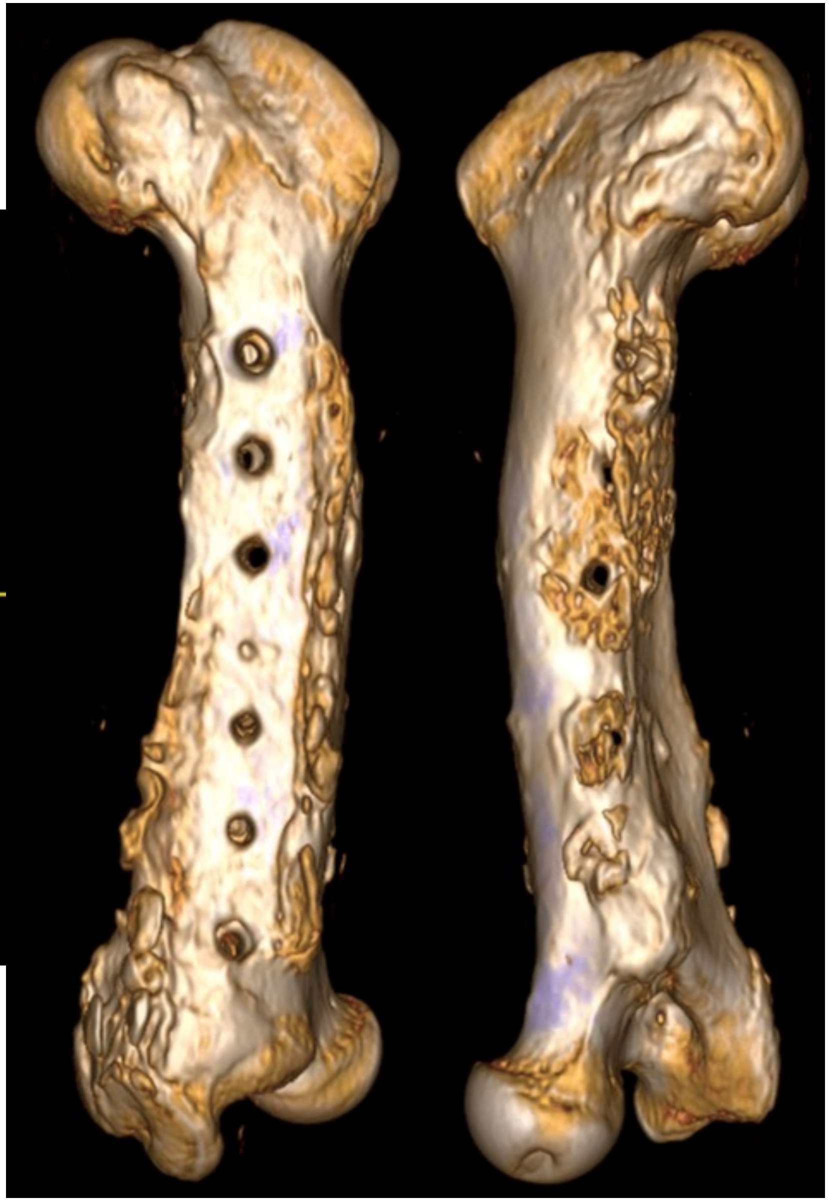

Control Group / No Infection
Pig No. 7

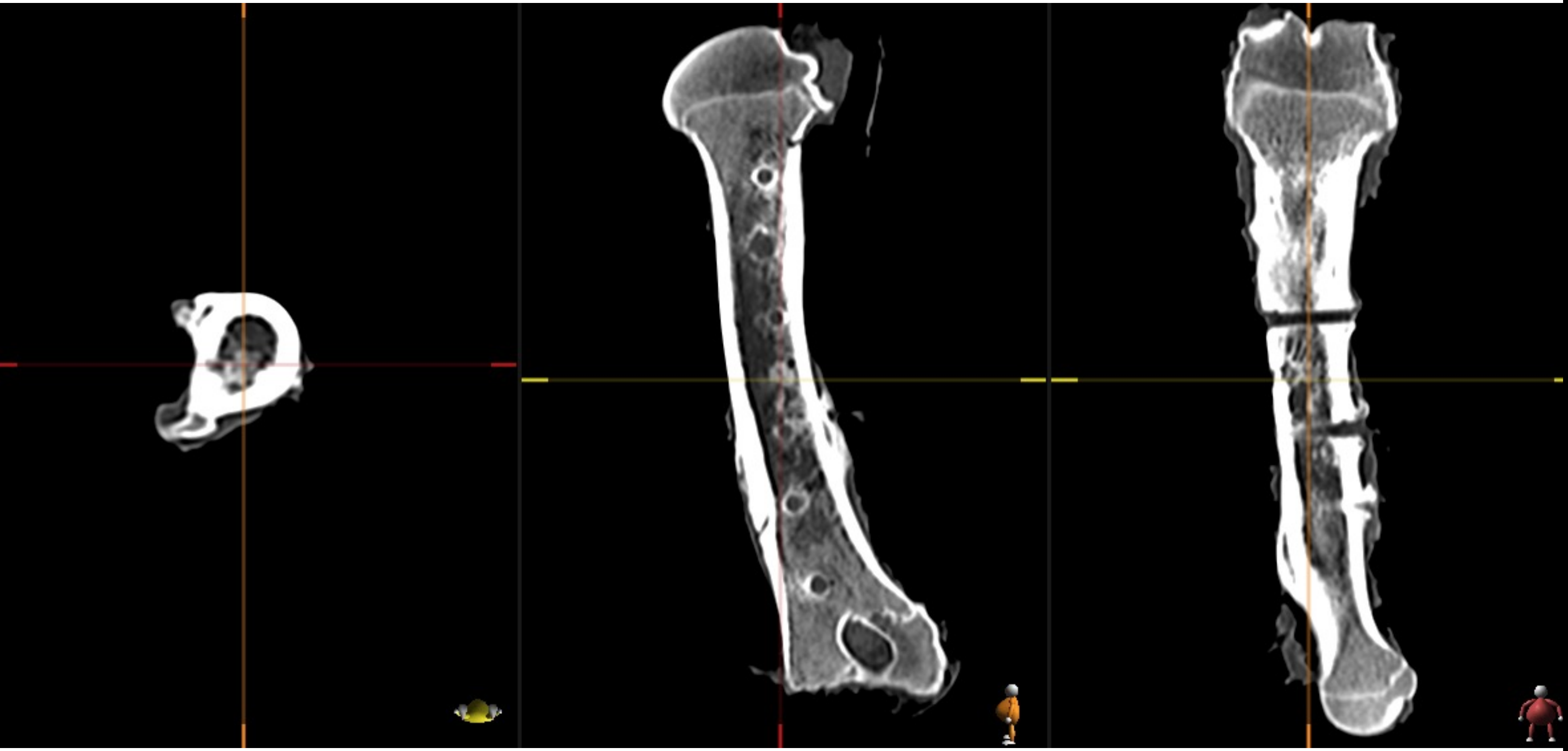

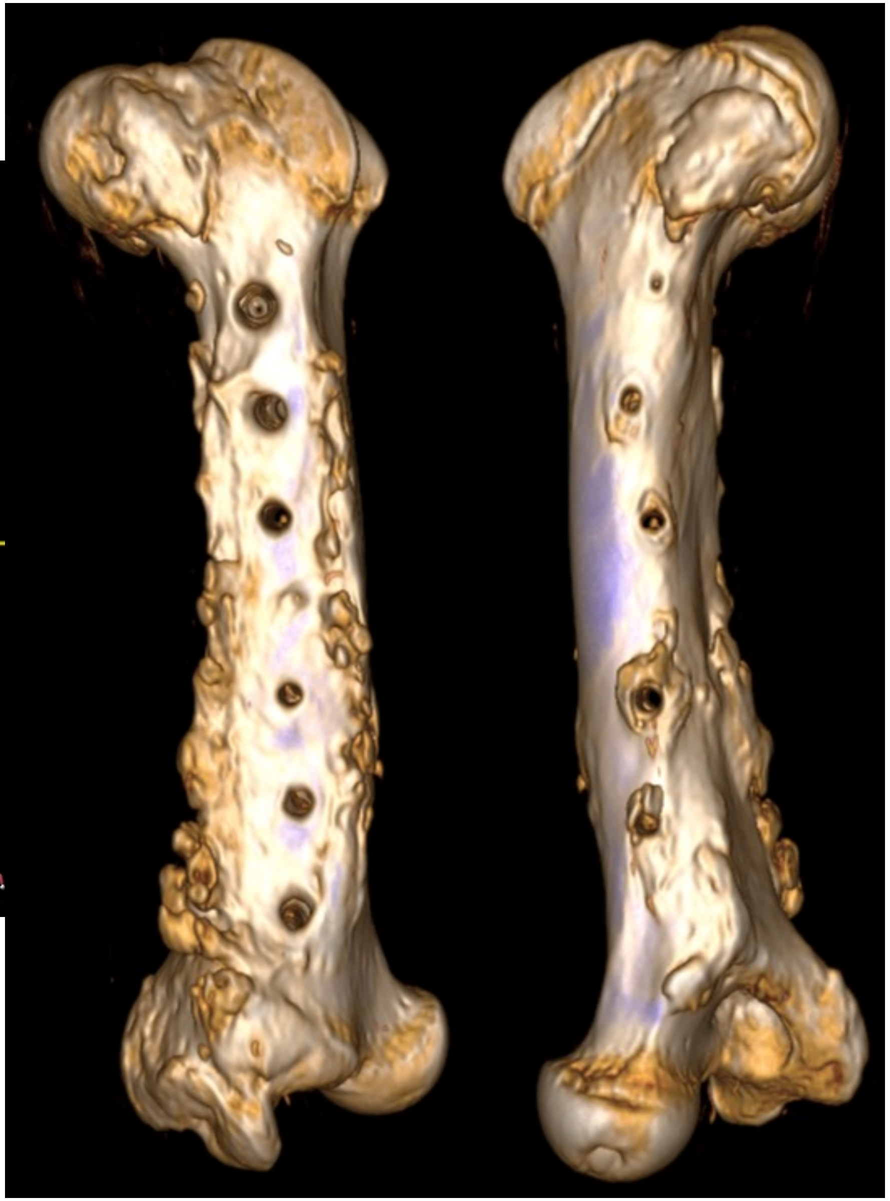

Control Group / No Infection
Pig No. 8

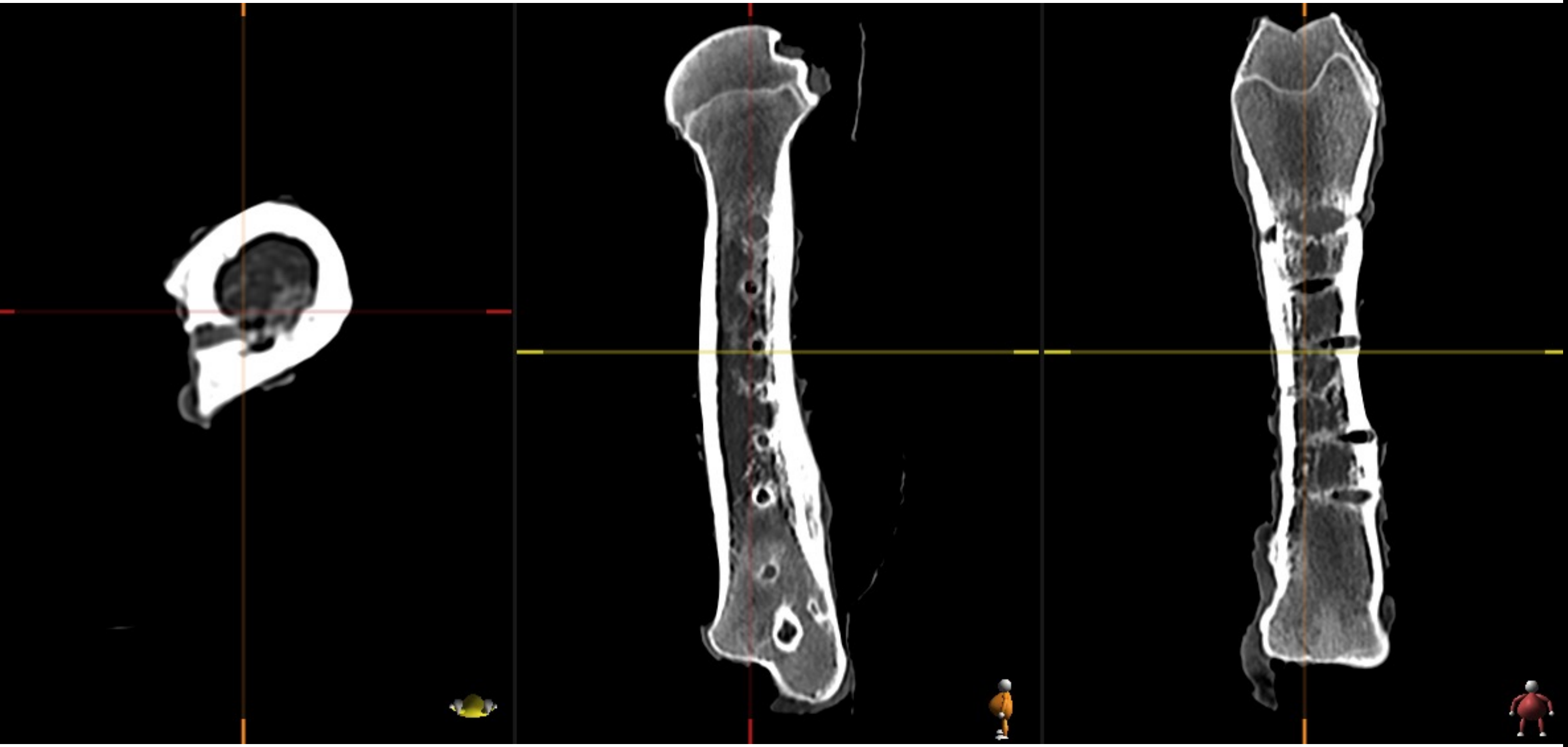

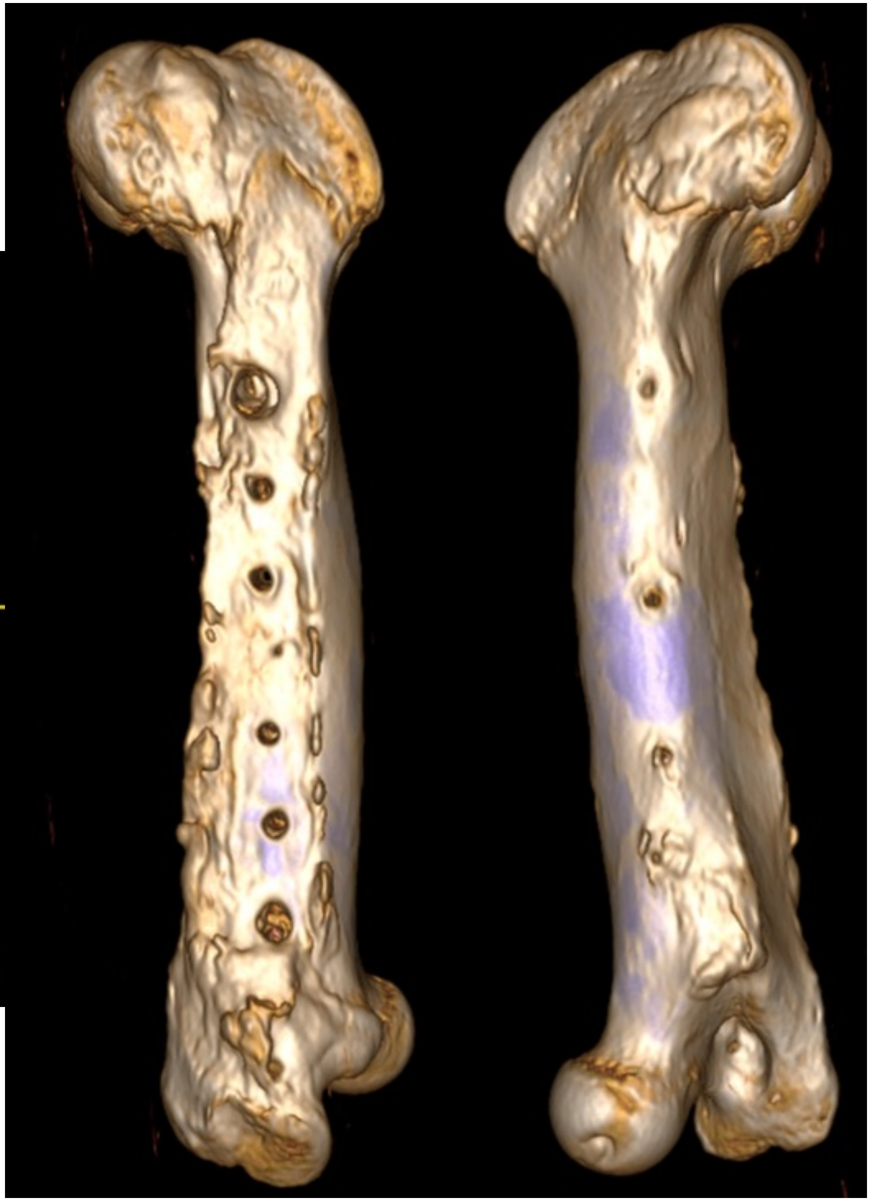

Control Group / No Infection
Pig No. 9

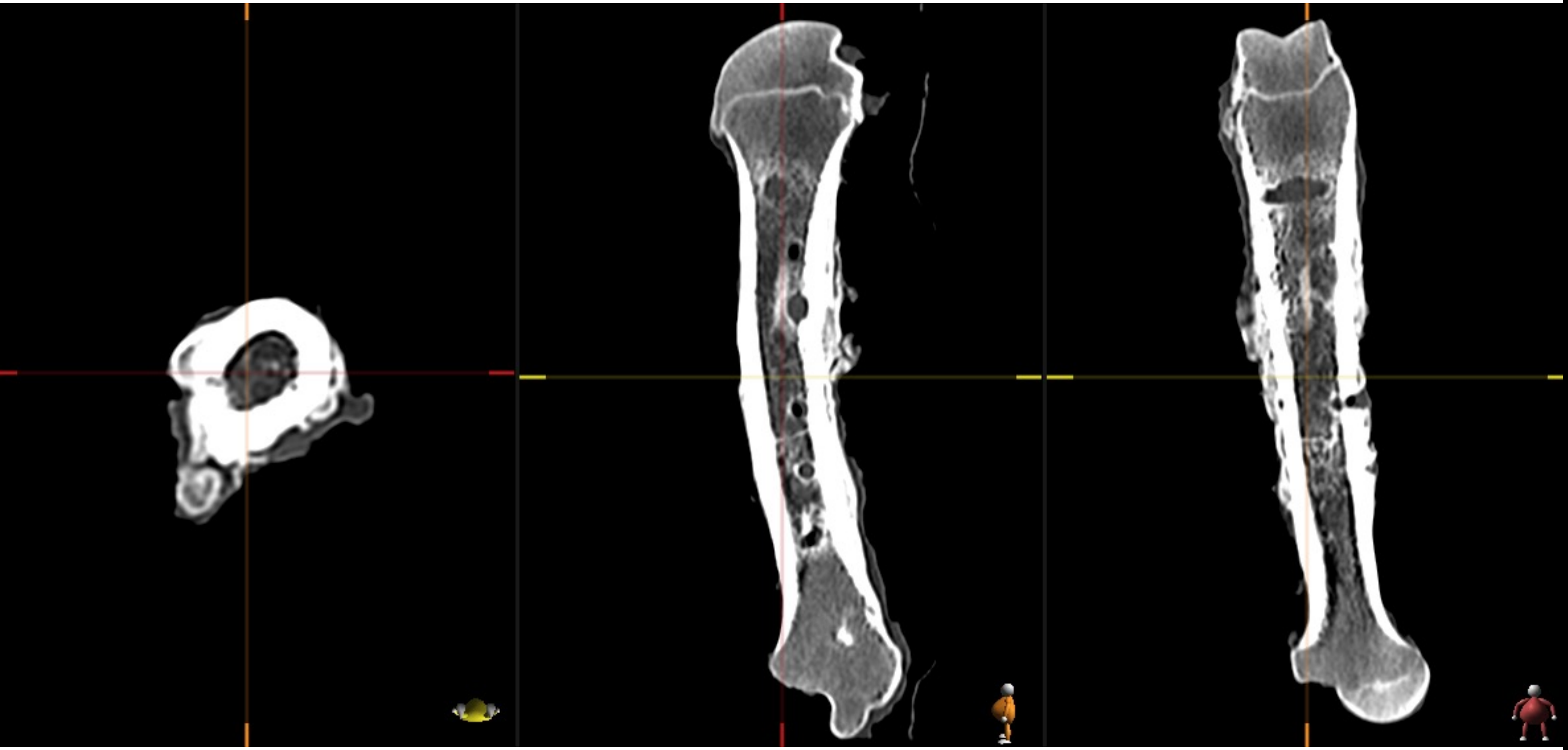

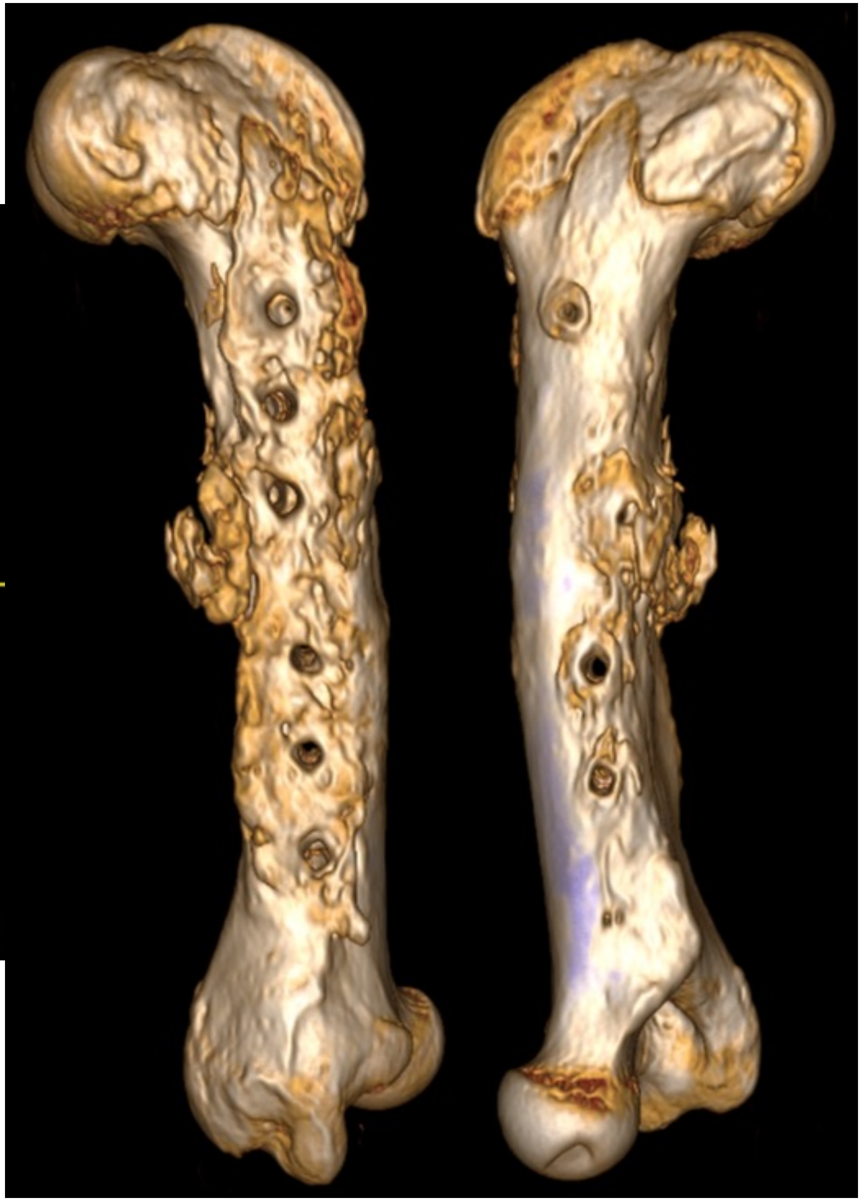

Control Group / No Infection
Pig No. 10

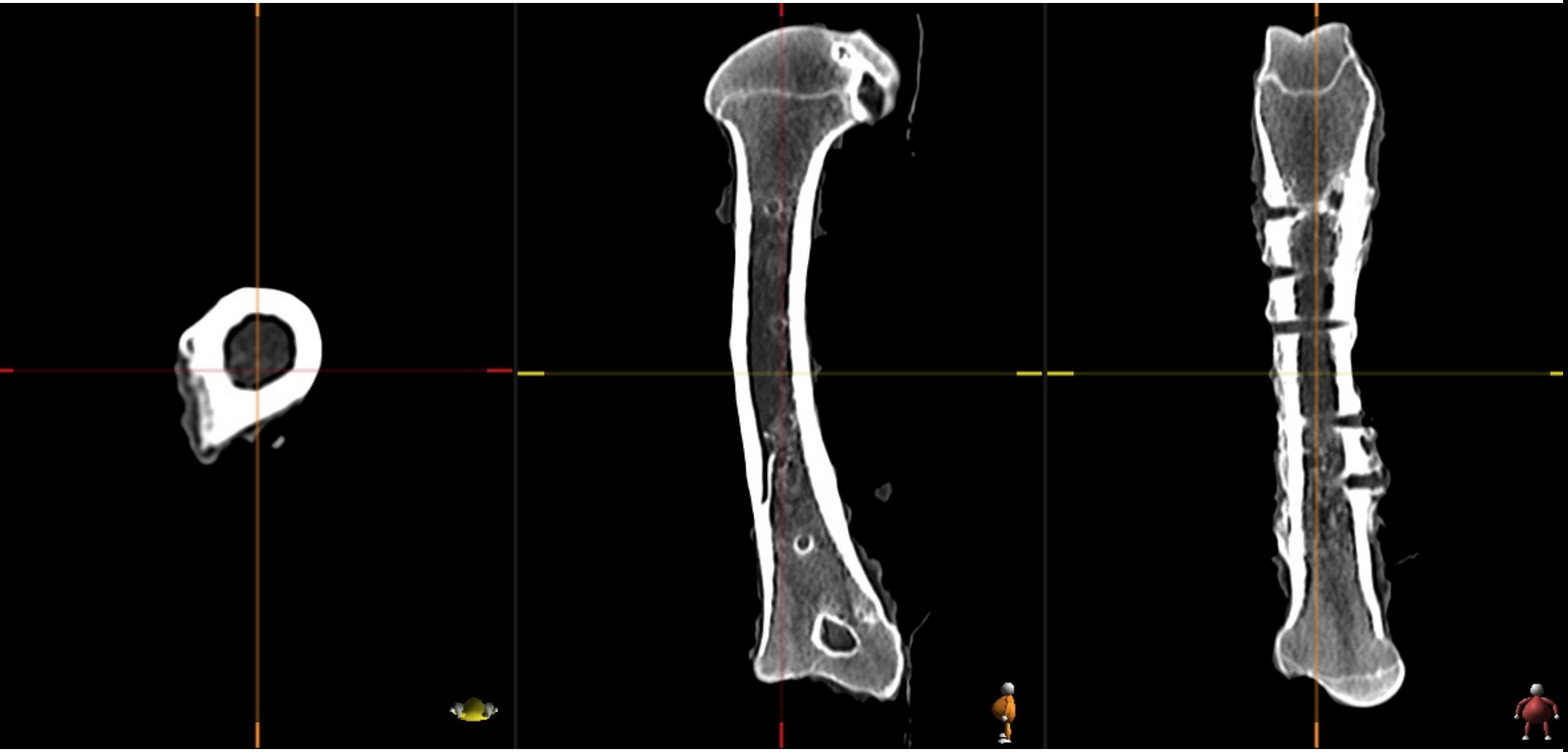

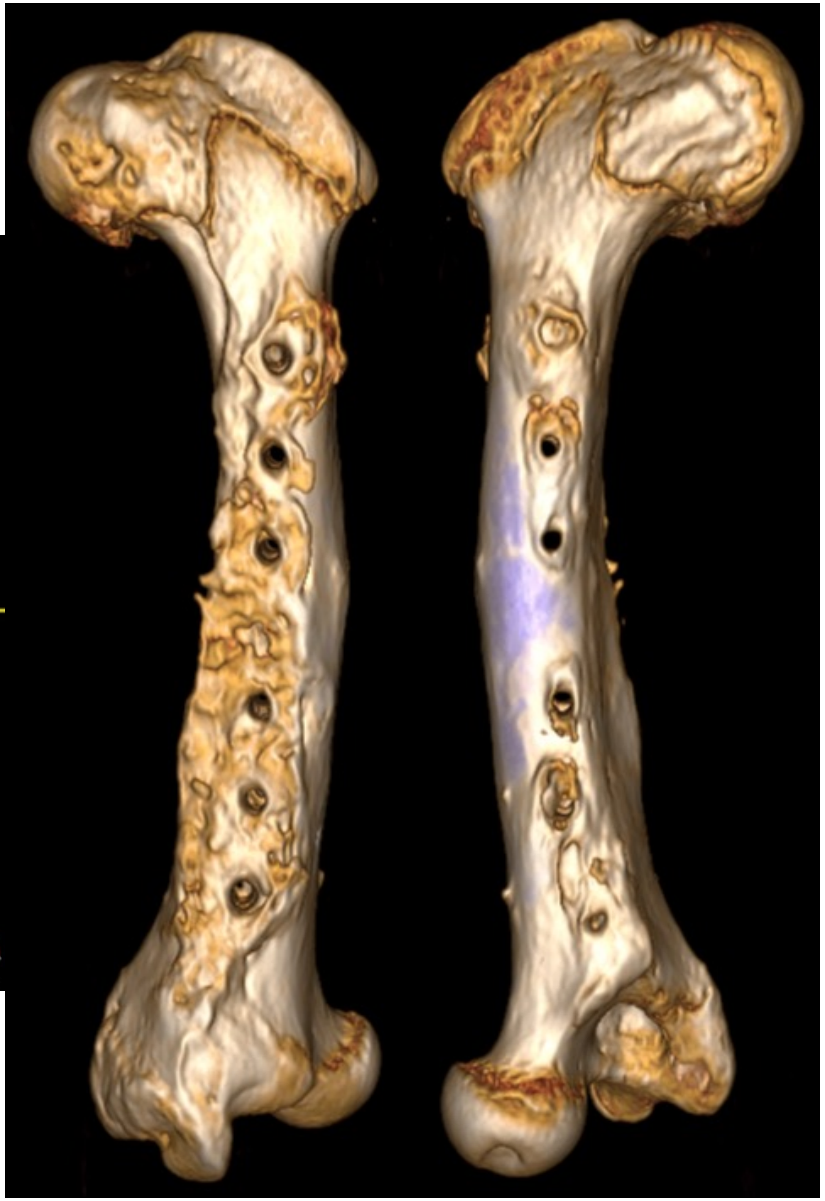

Control Group / No Infection
Pig No. 11

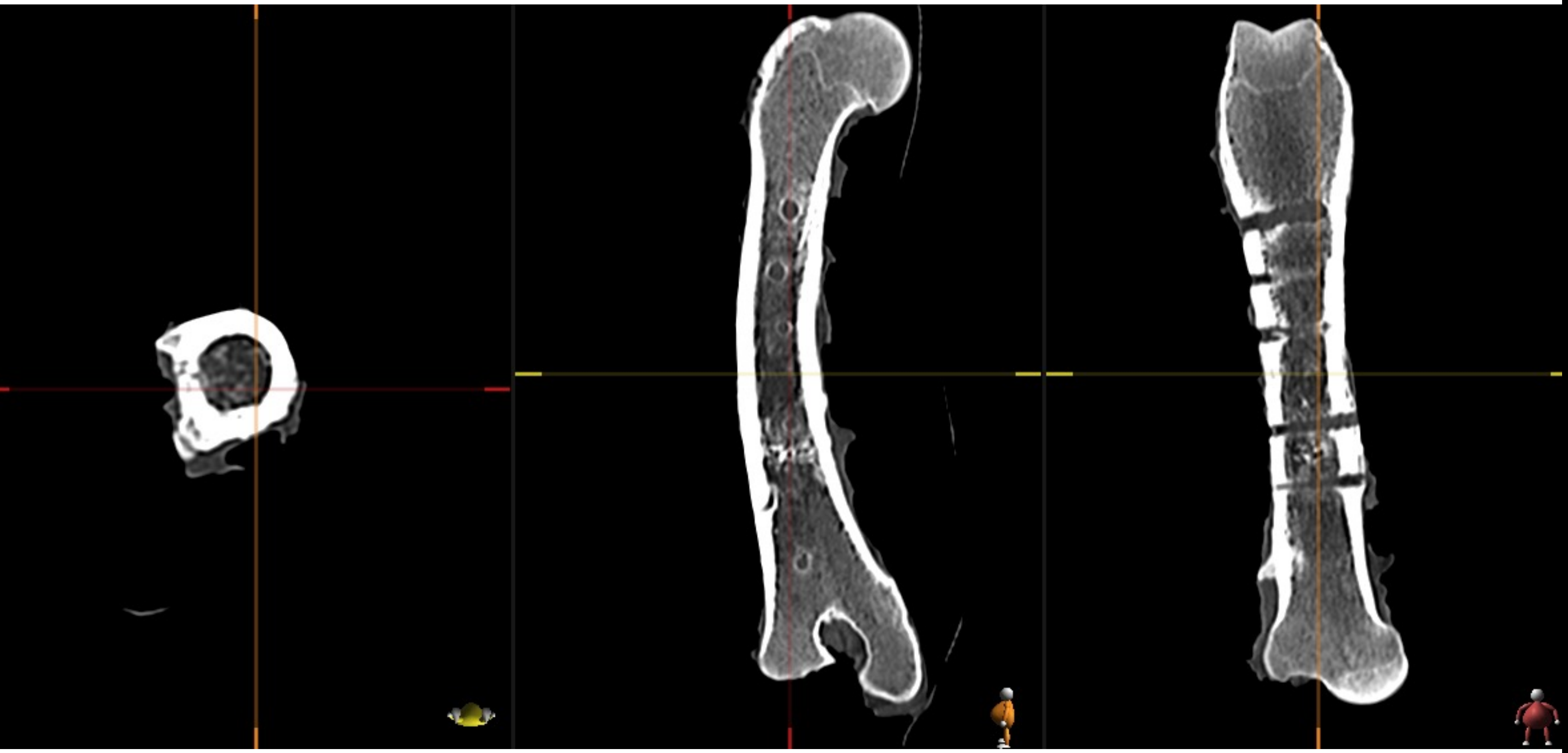

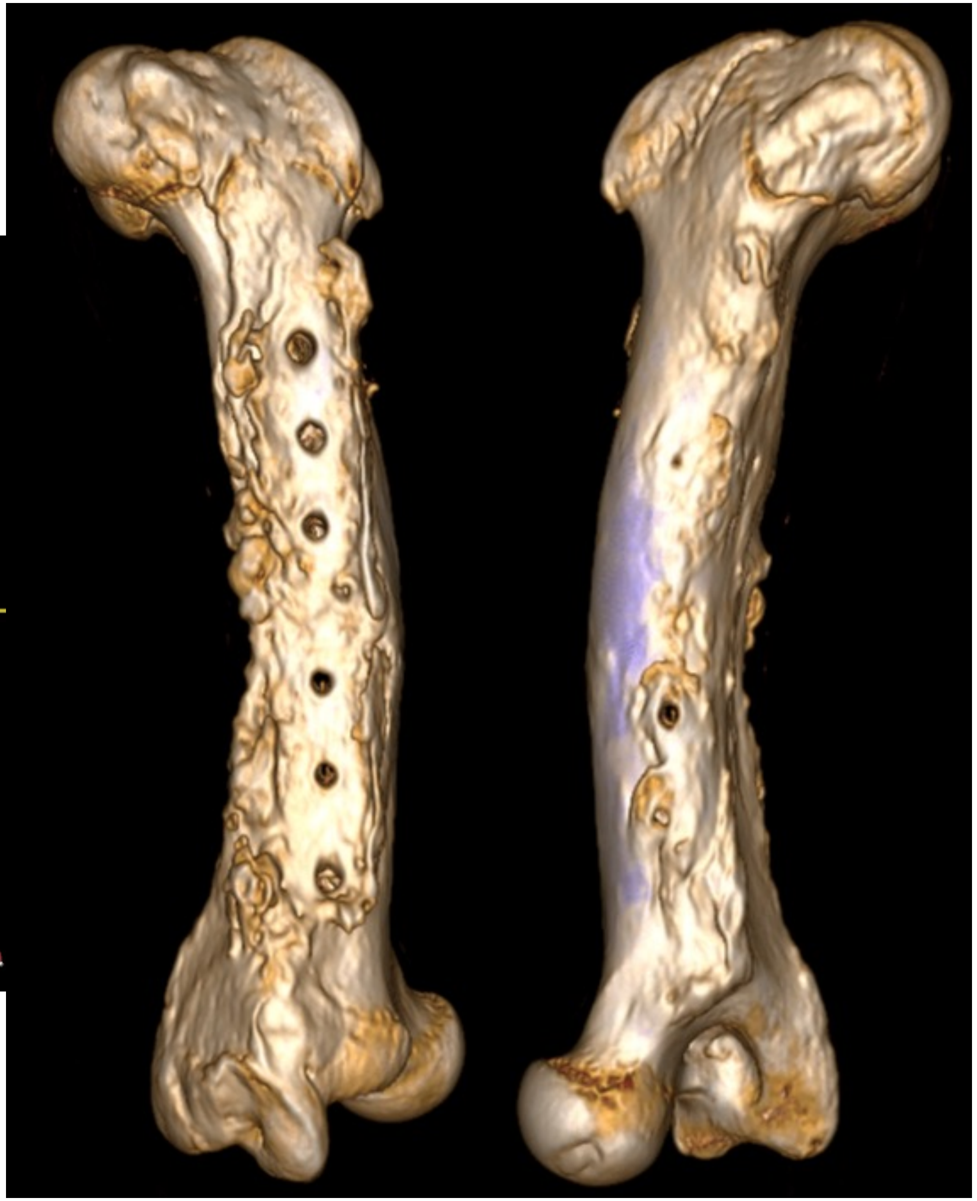

Control Group / No Infection
Pig No. 12

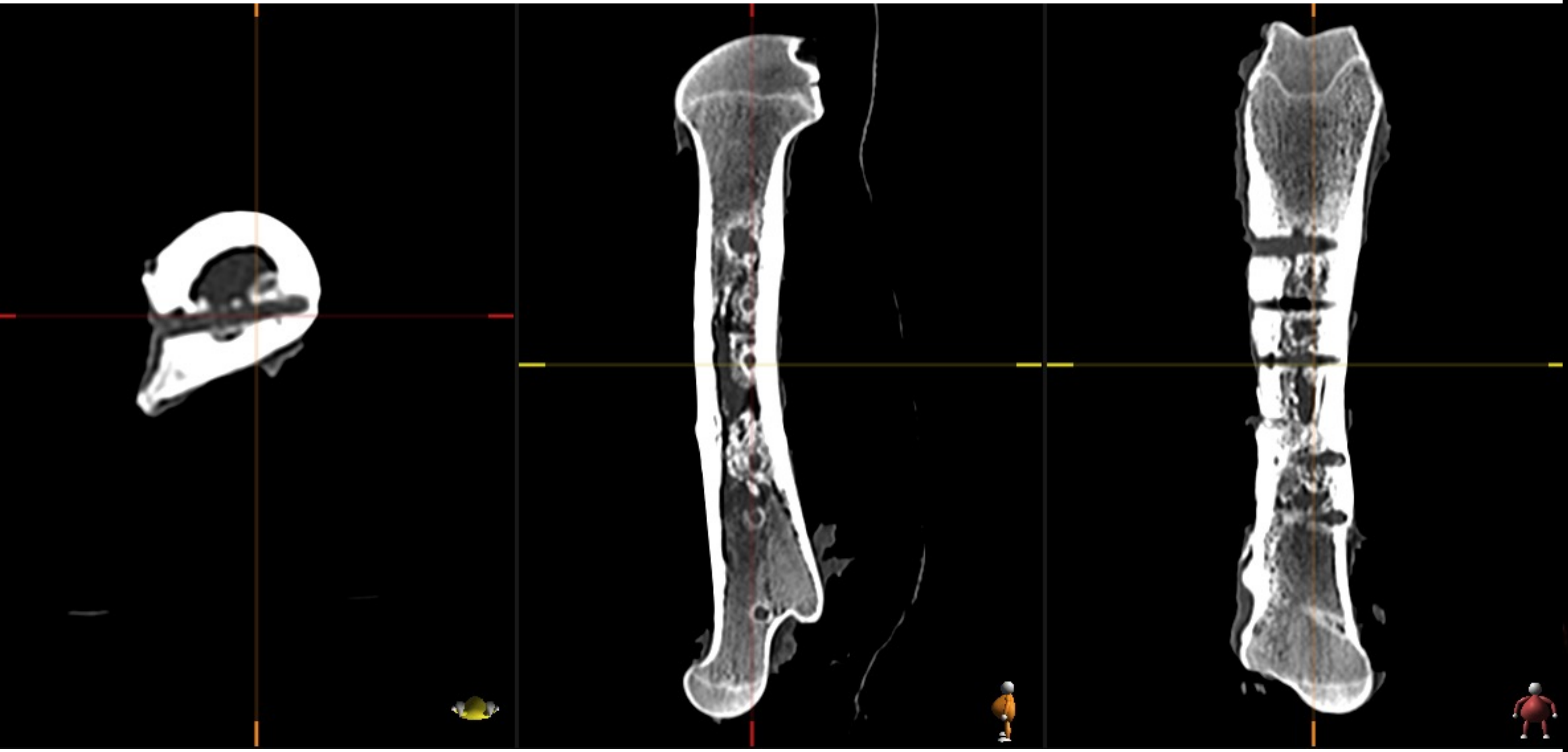

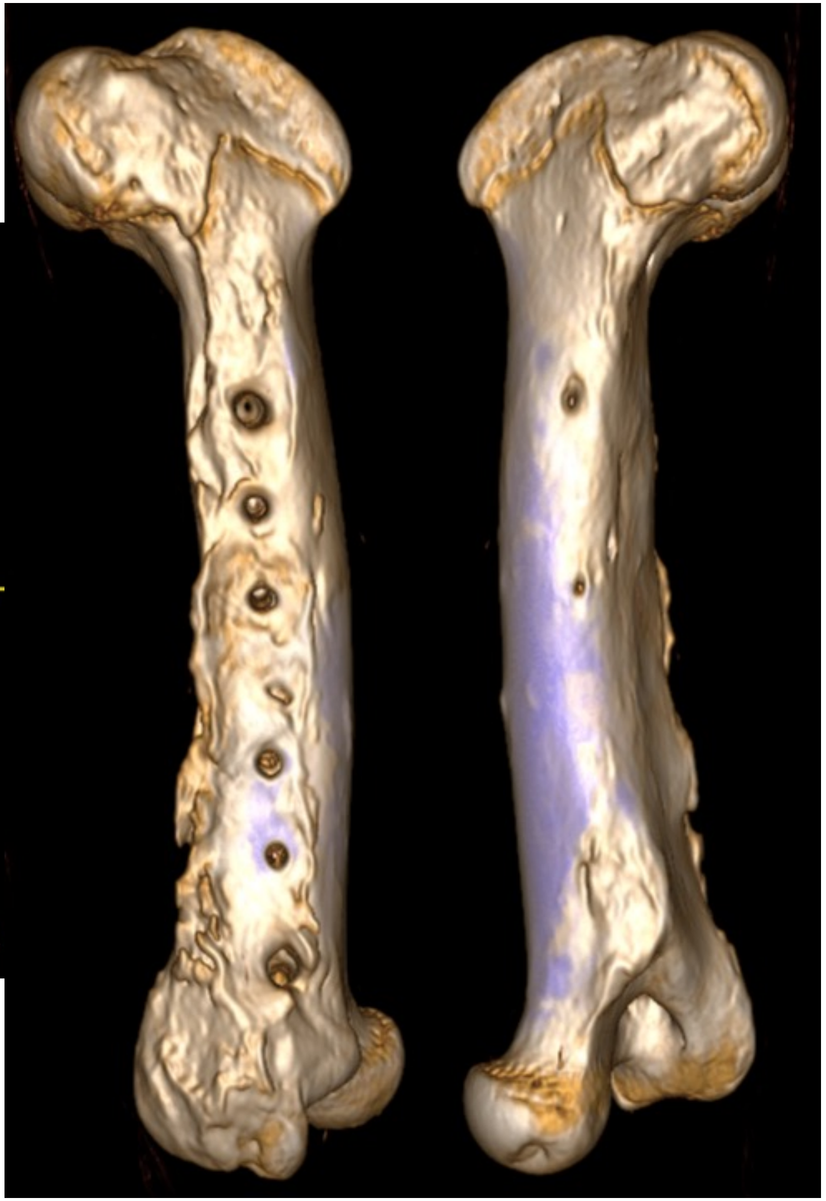

Control Group / No Infection
Pig No. 13

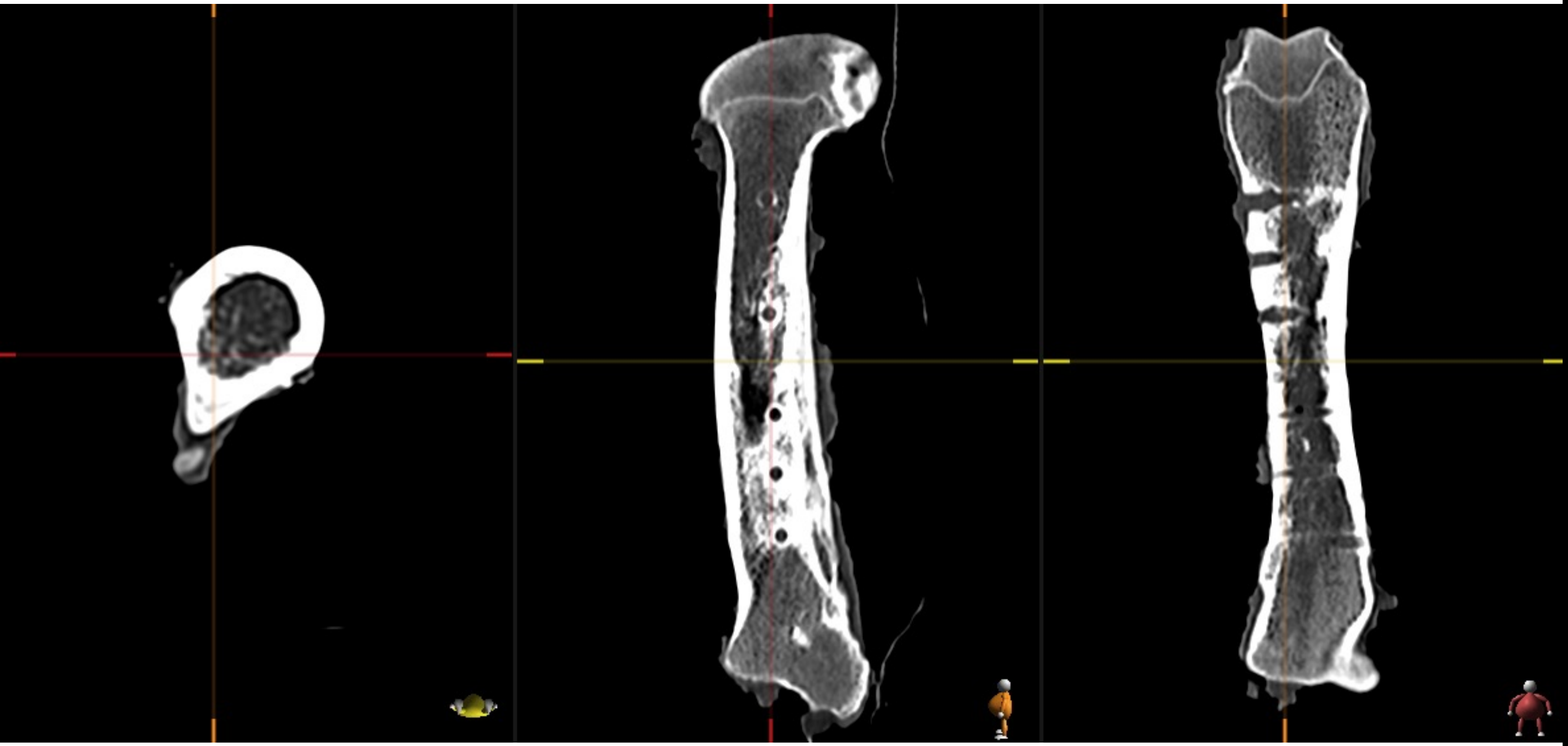

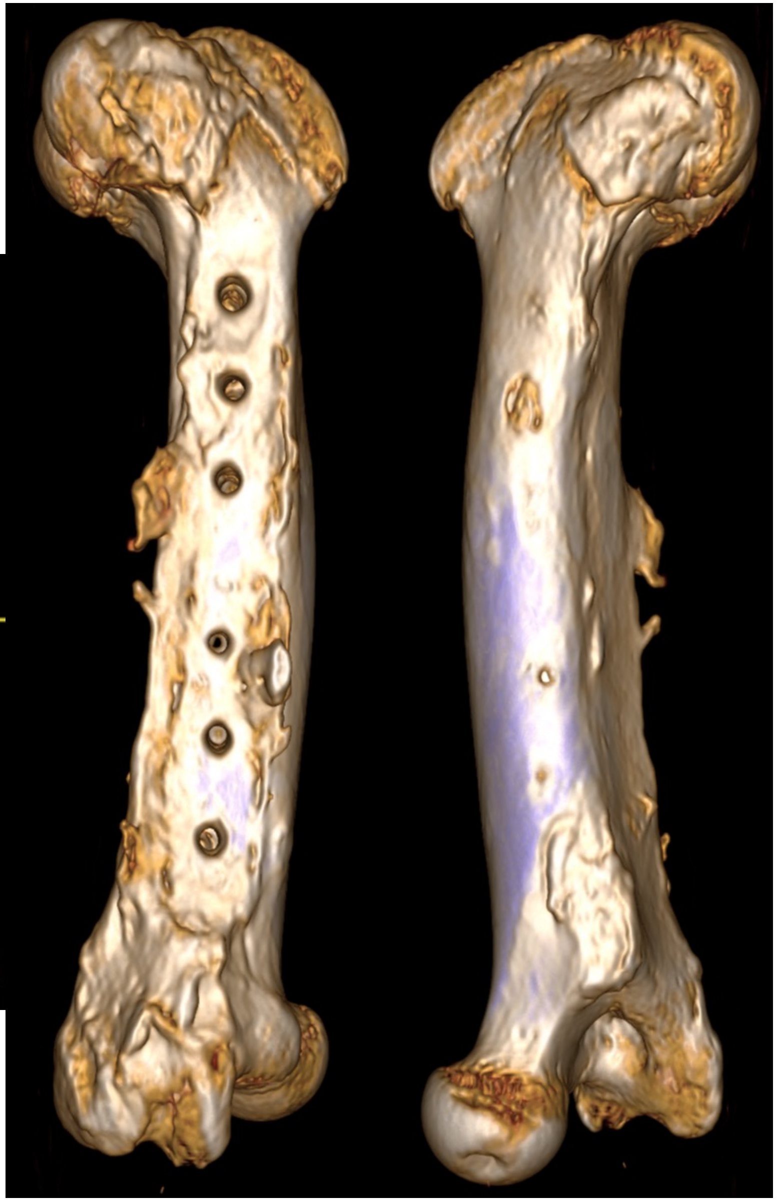

Control Group / No Infection
Pig No. 14

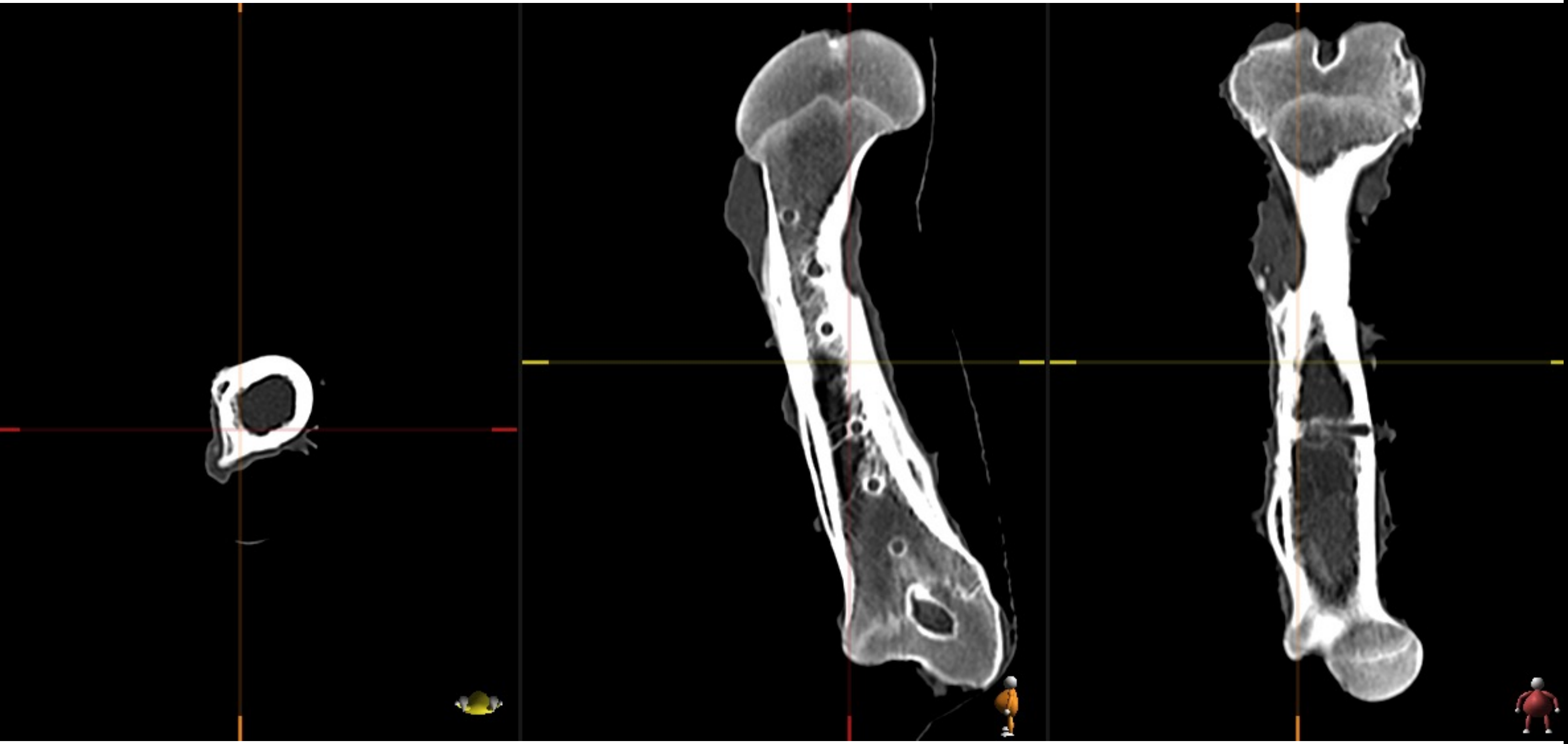

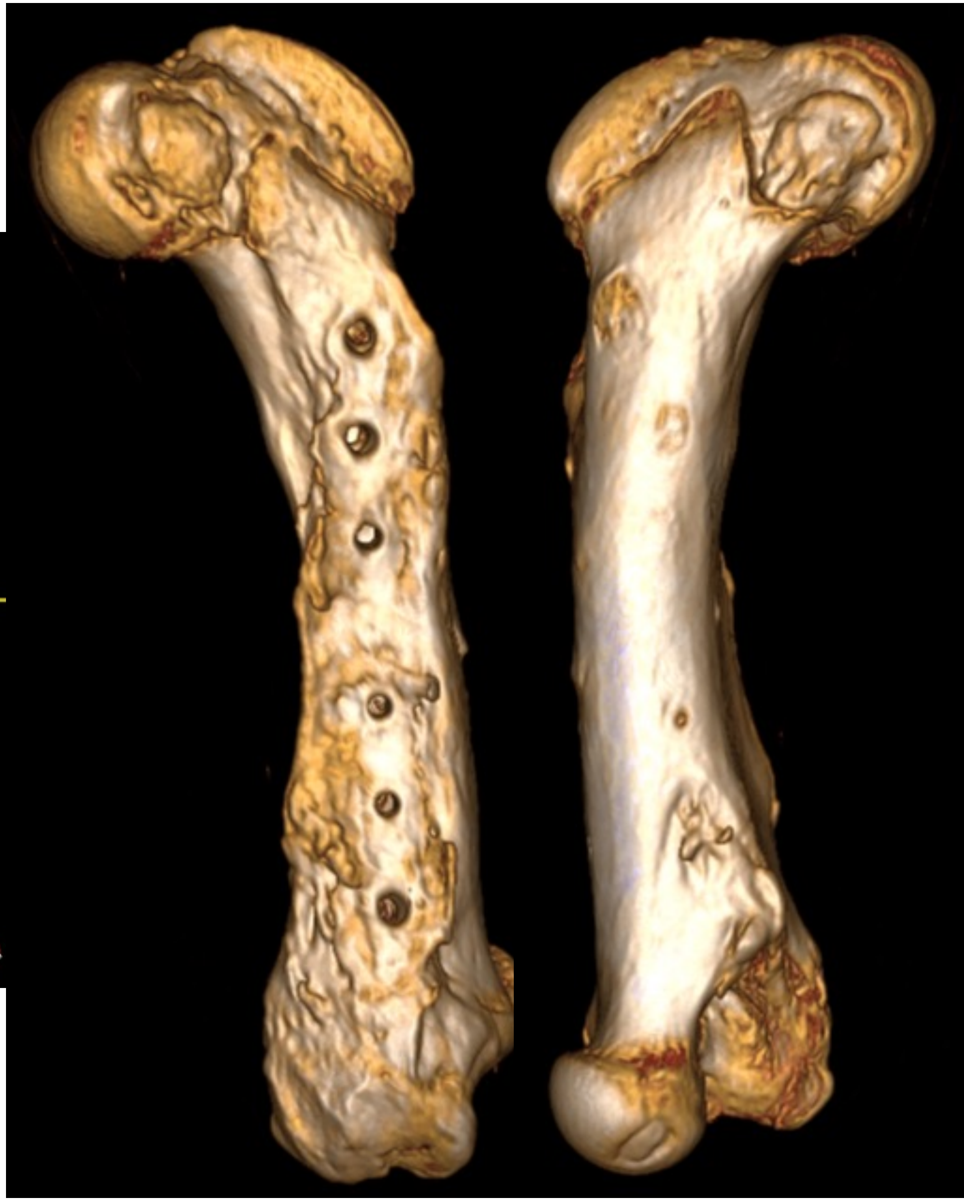

Control Group / No Infection
Pig No. 15

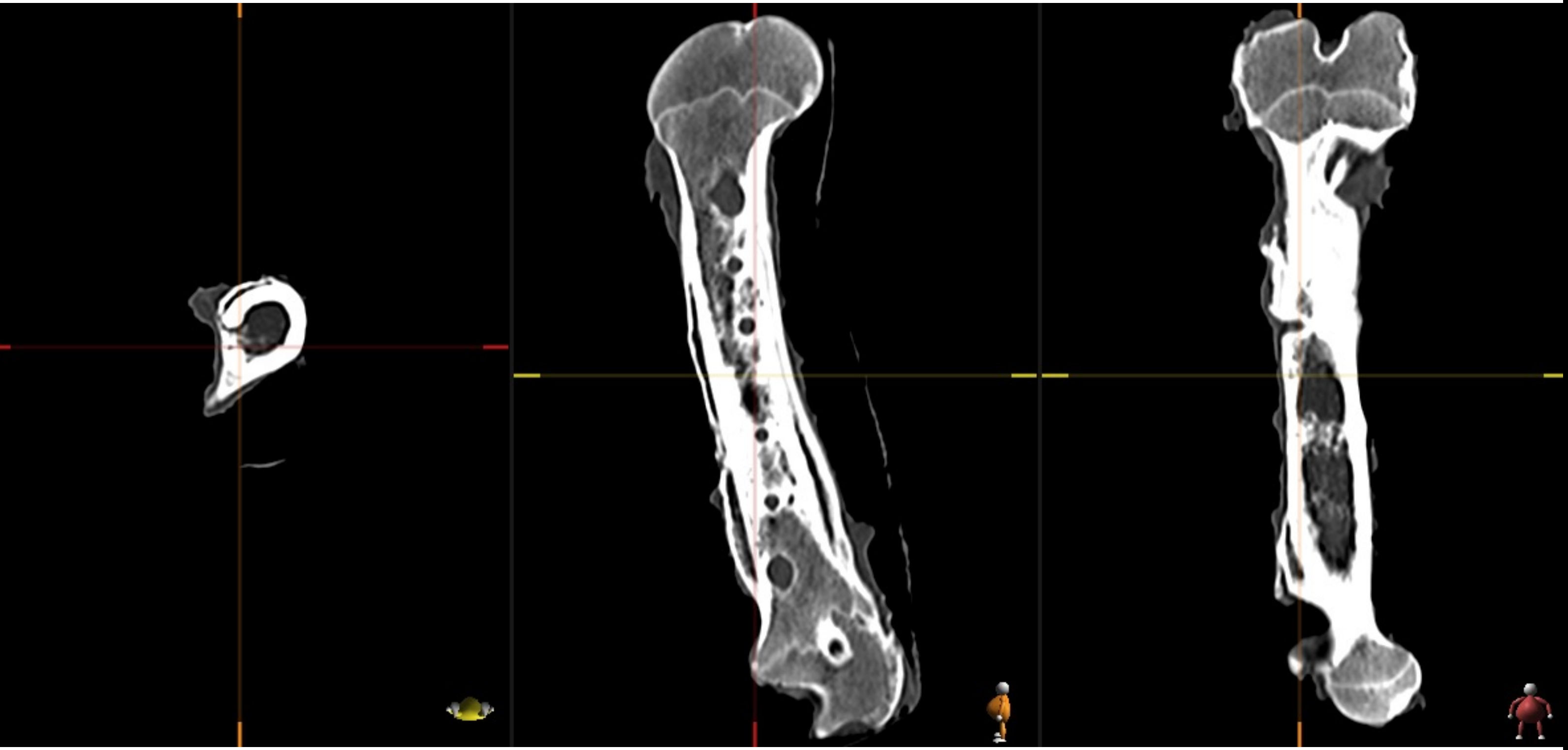

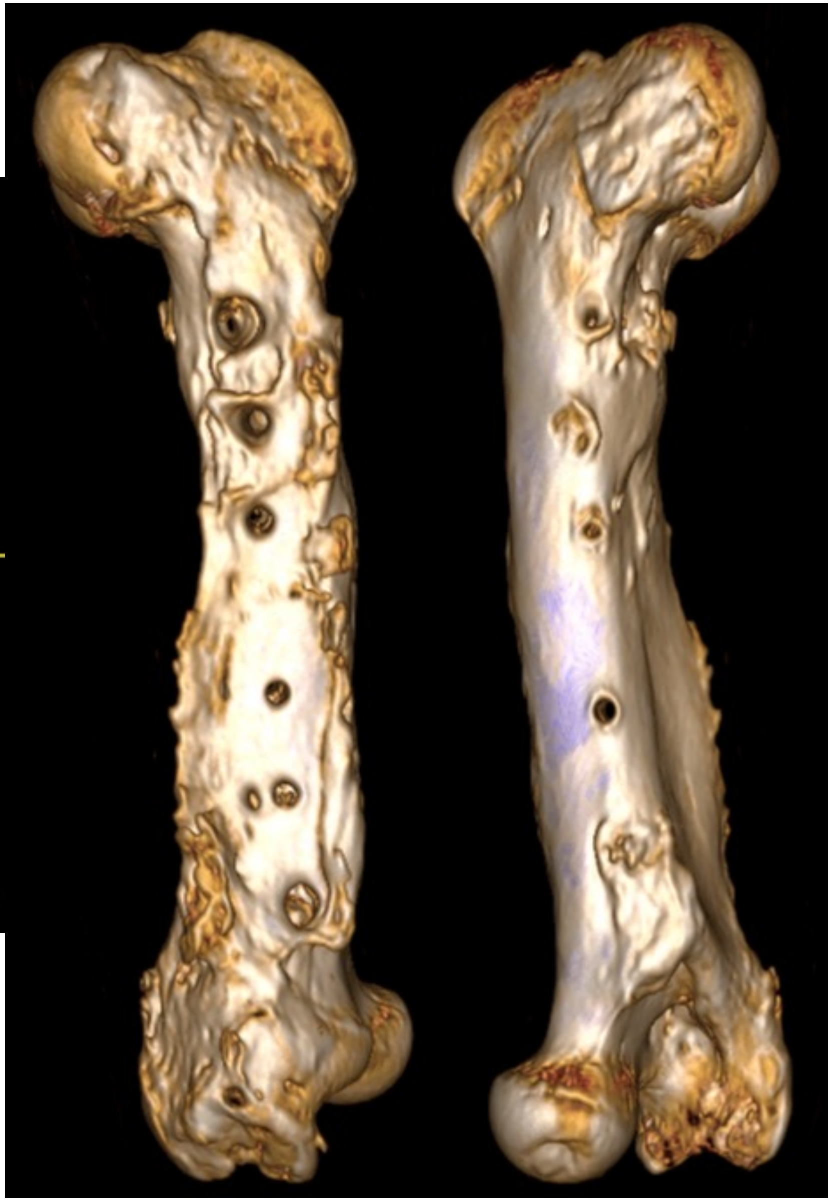

Infection Group / Infection and Uncoated Plate
Pig No. 1

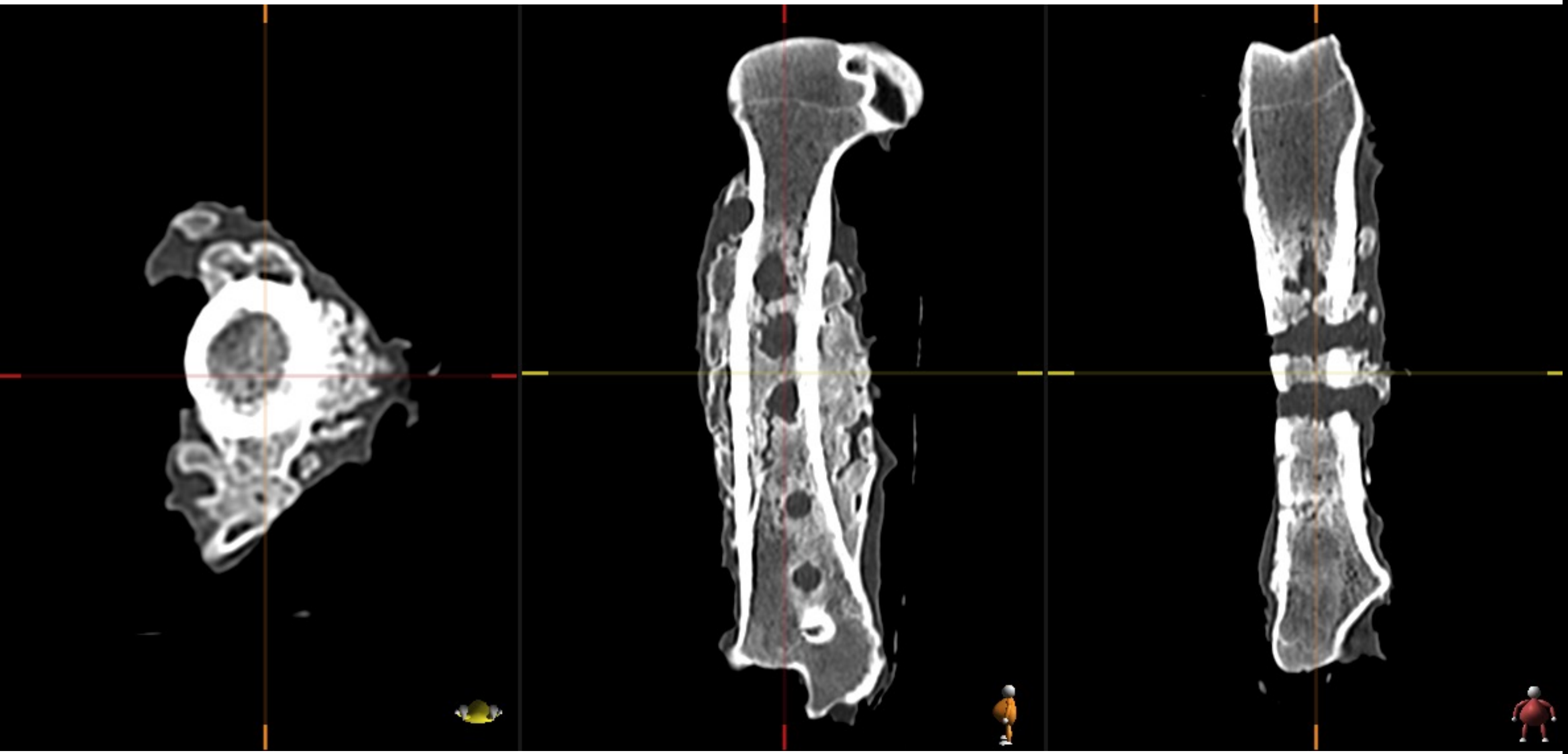

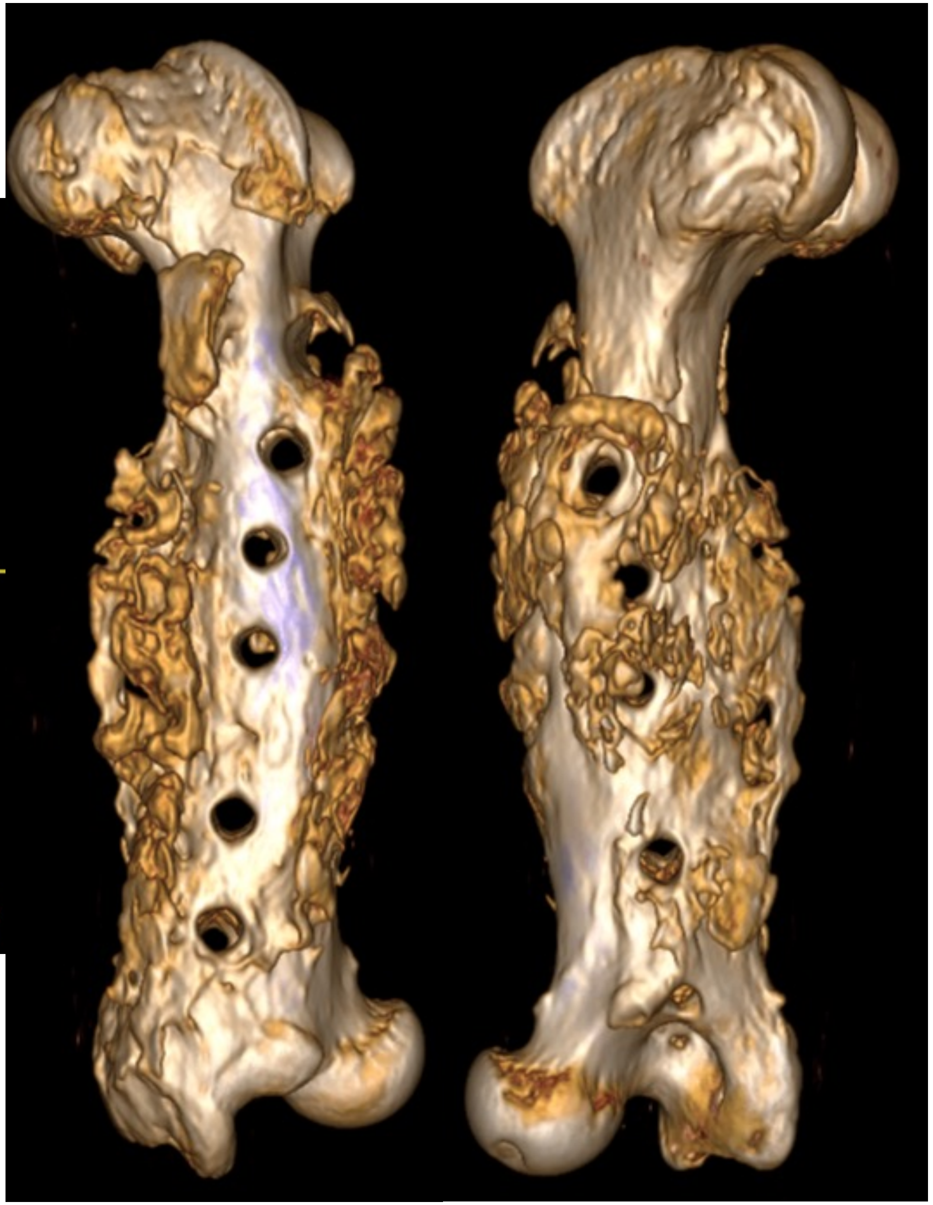

Infection Group / Infection and Uncoated Plate
Pig No. 2

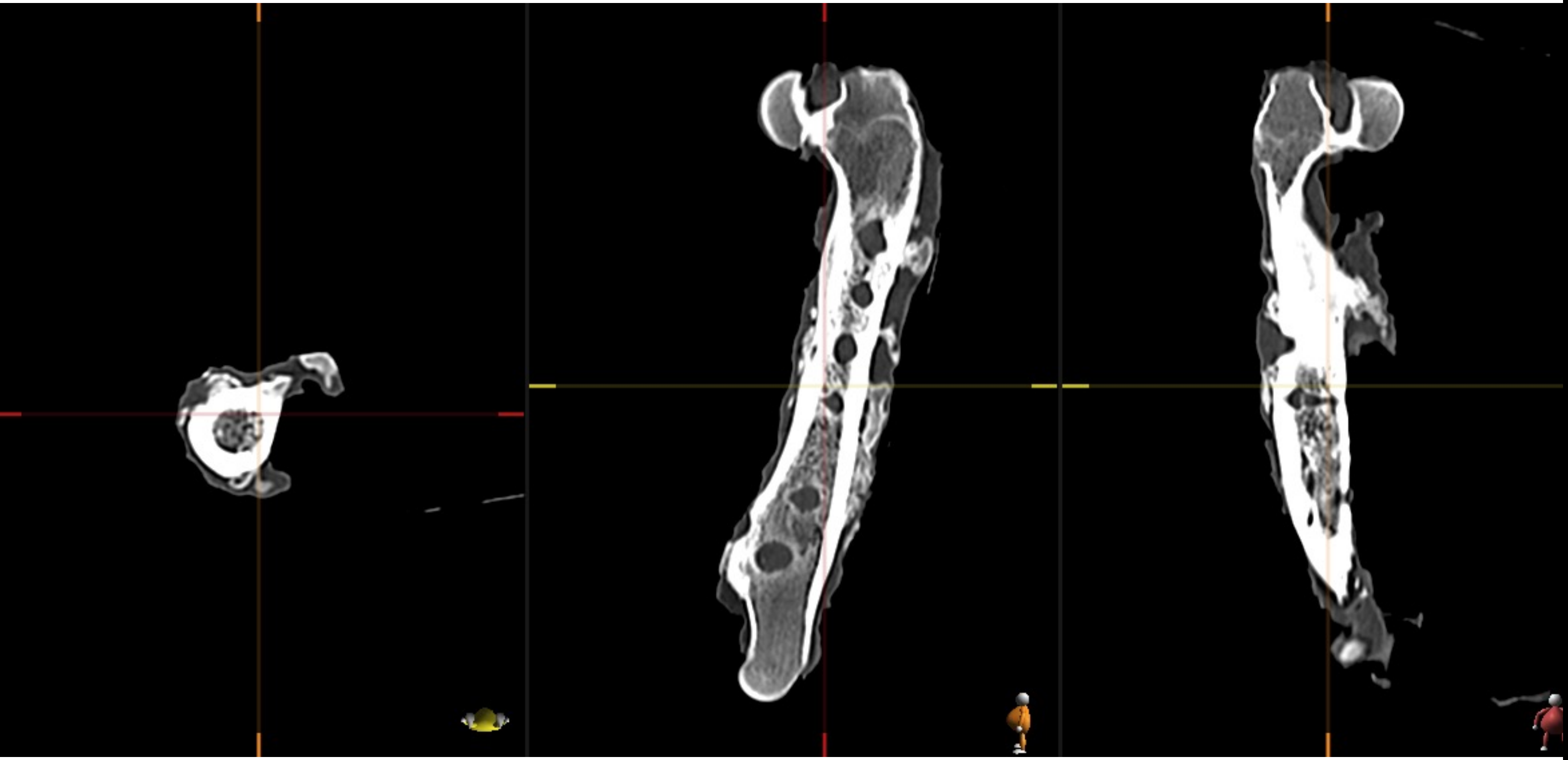

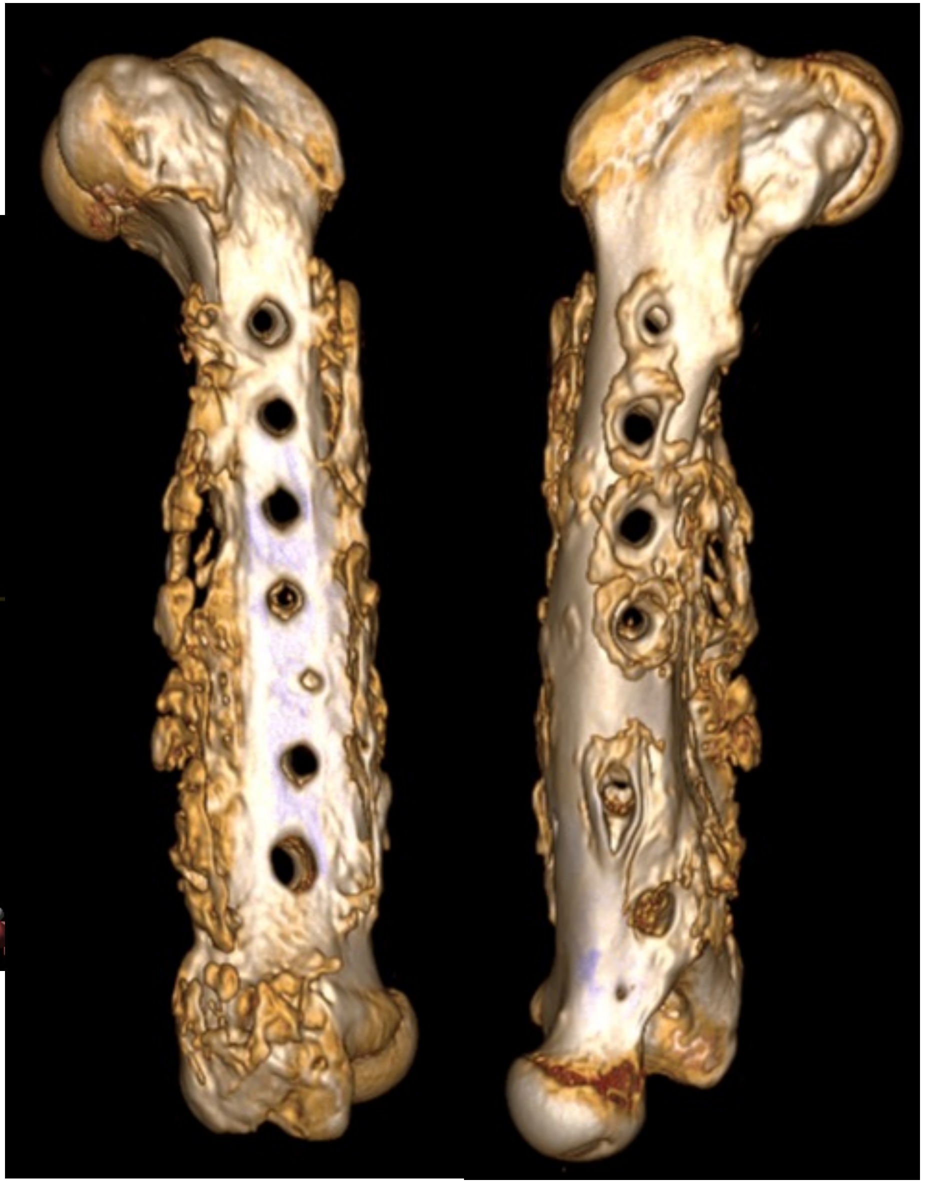

Infection Group / Infection and Uncoated Plate
Pig No. 3

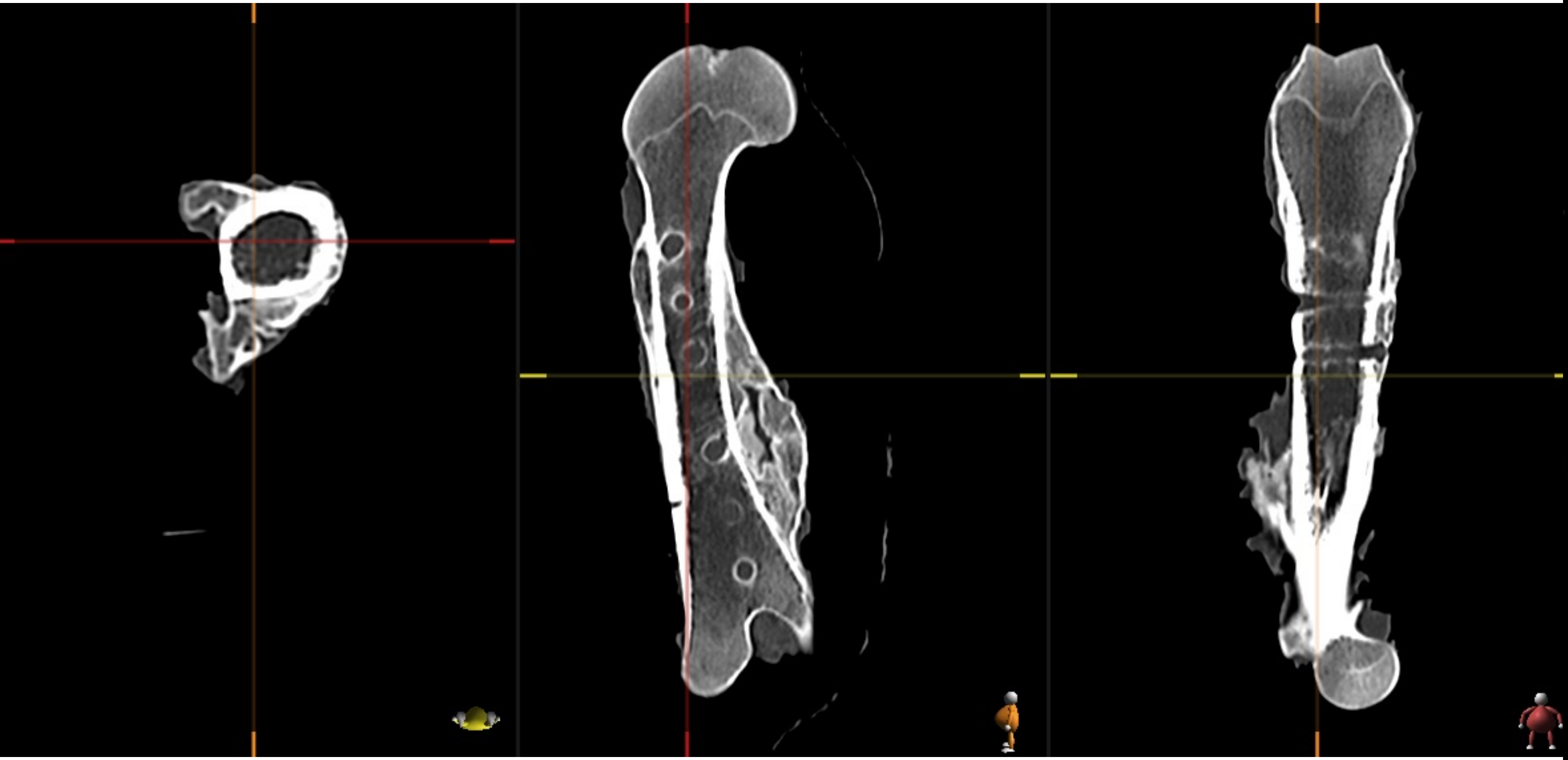

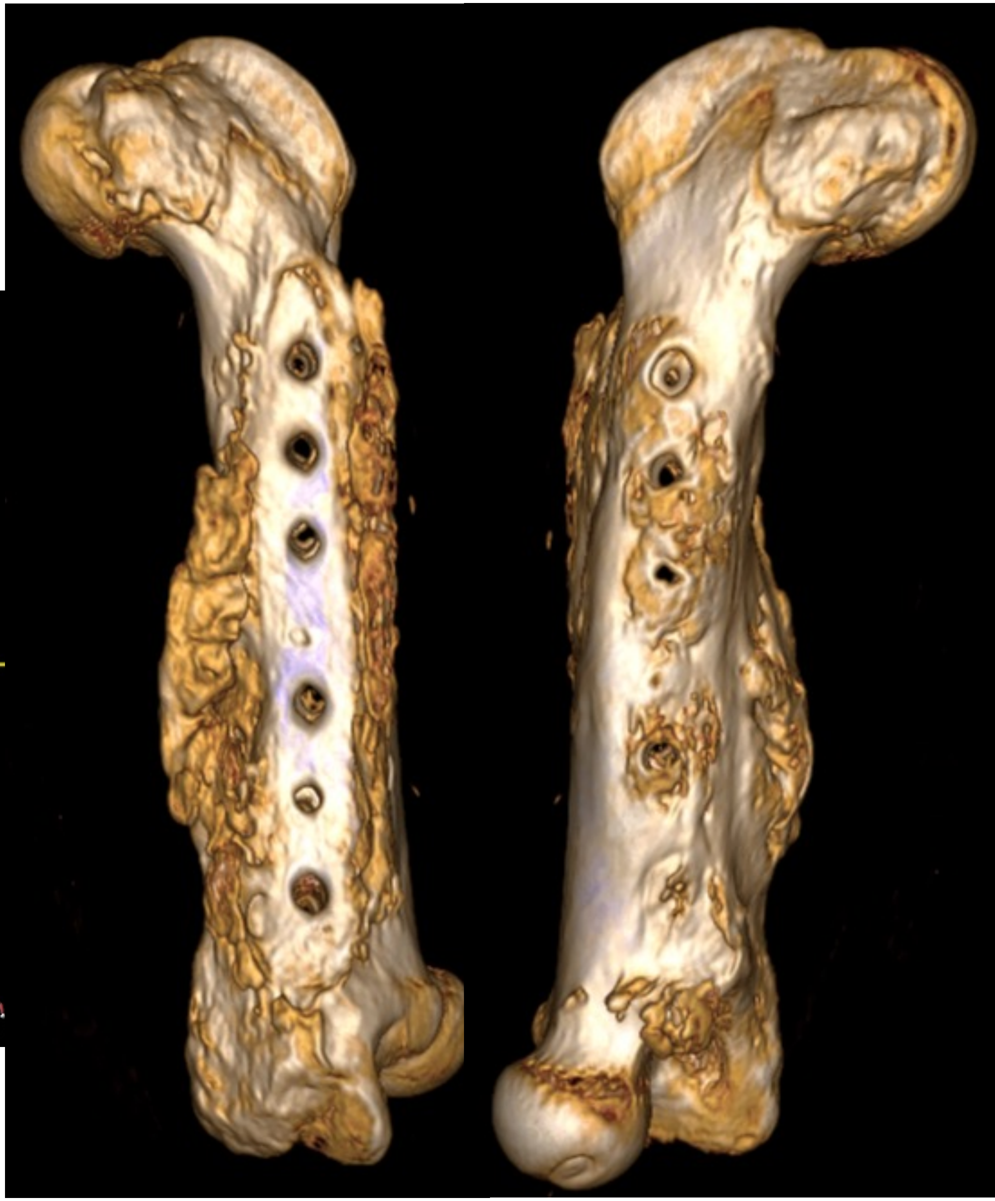

Infection Group / Infection and Uncoated Plate
Pig No. 4

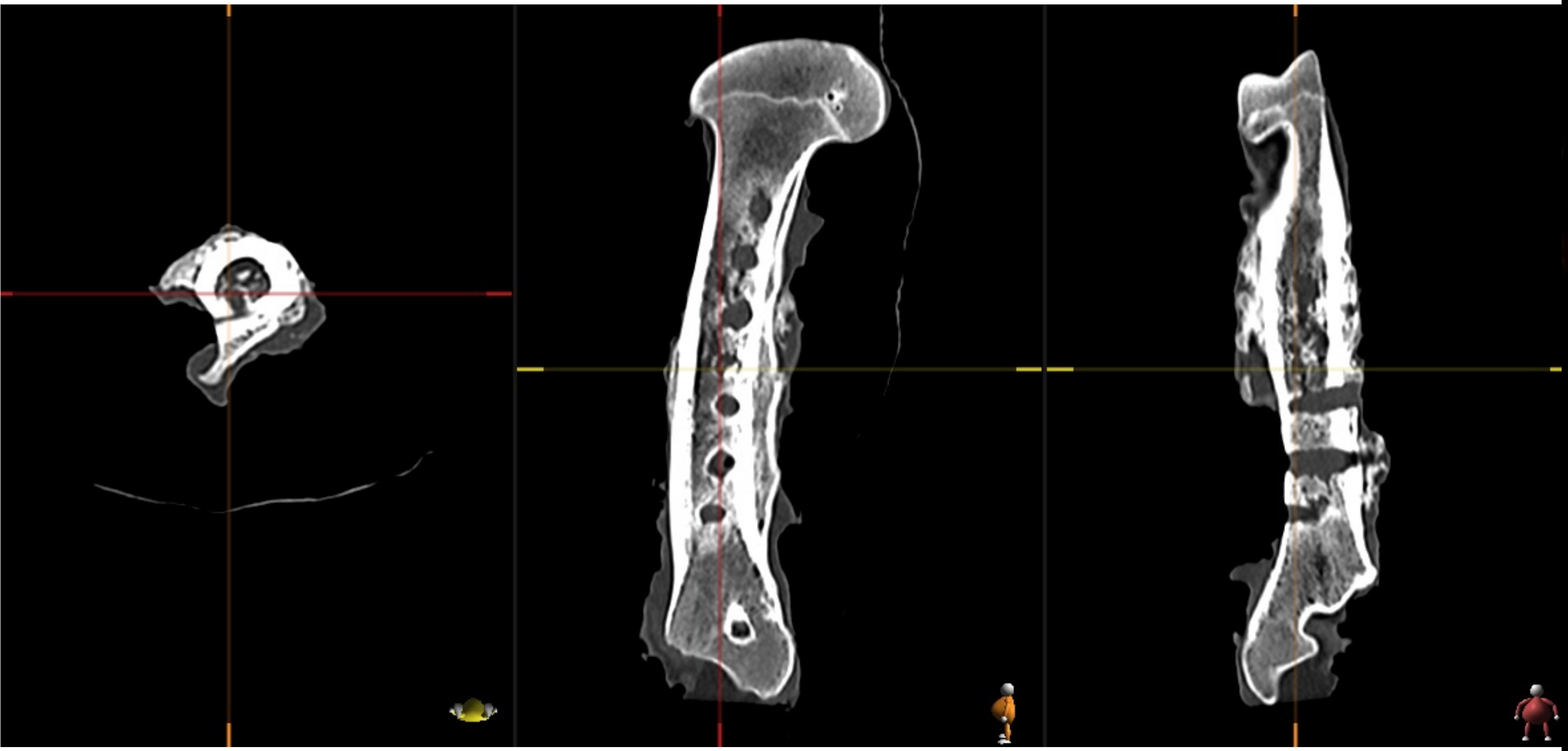

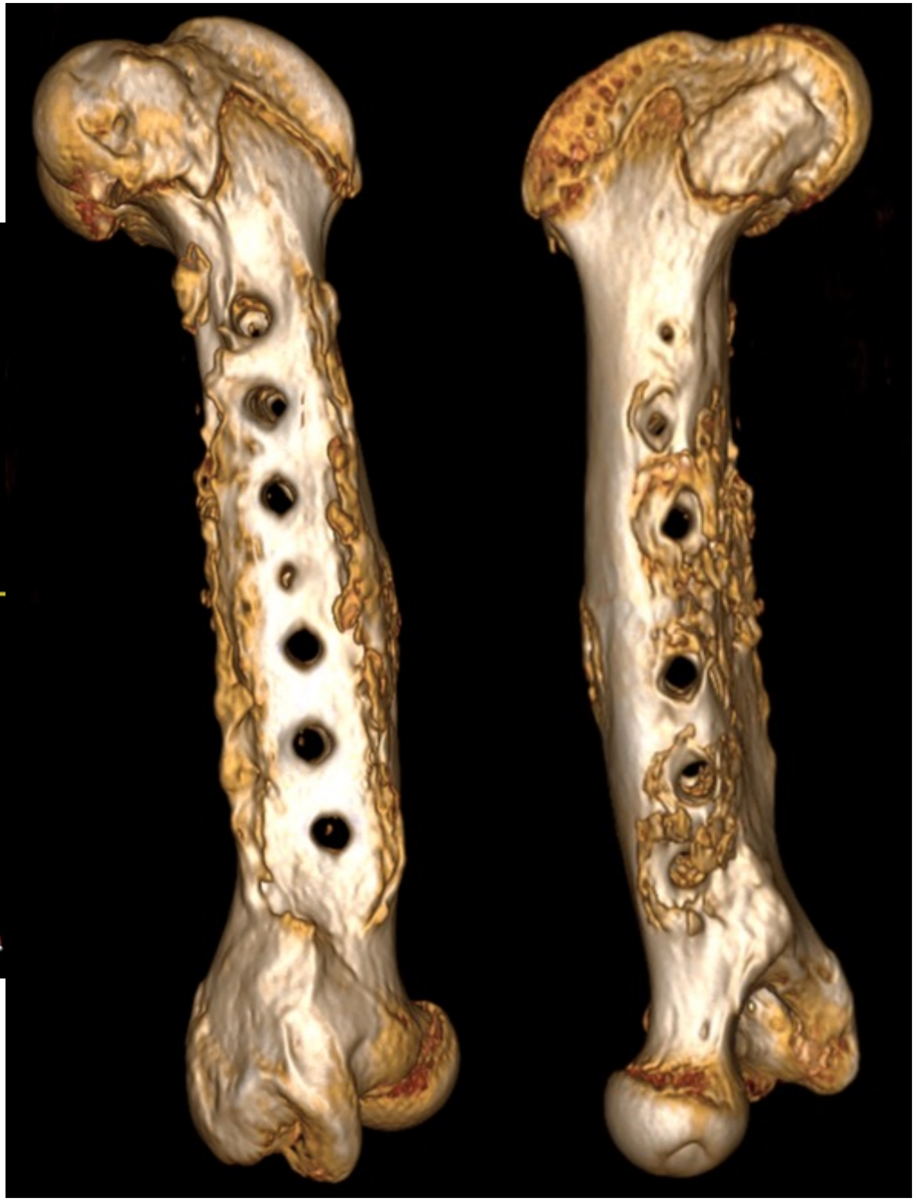

Infection Group / Infection and Uncoated Plate
Pig No. 5

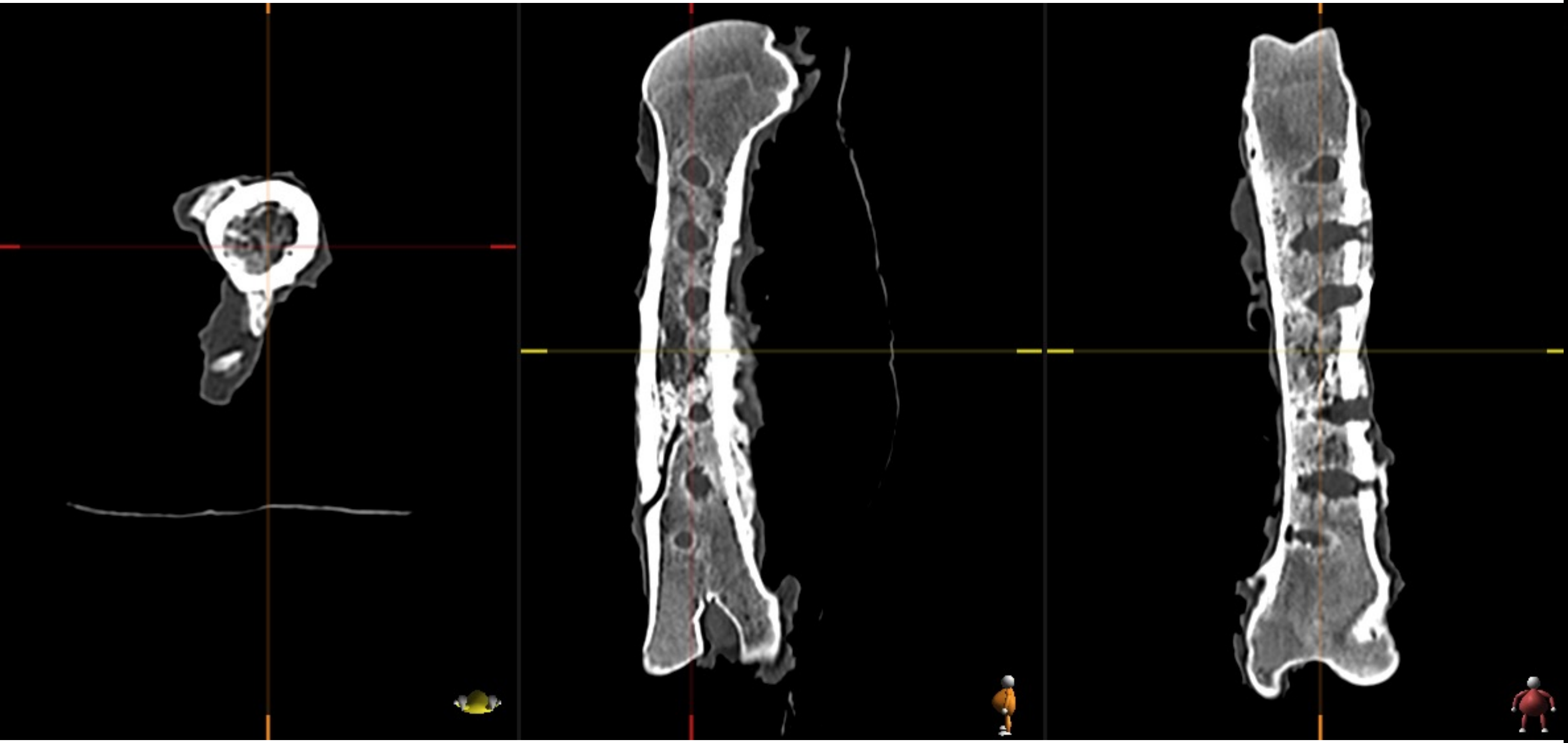

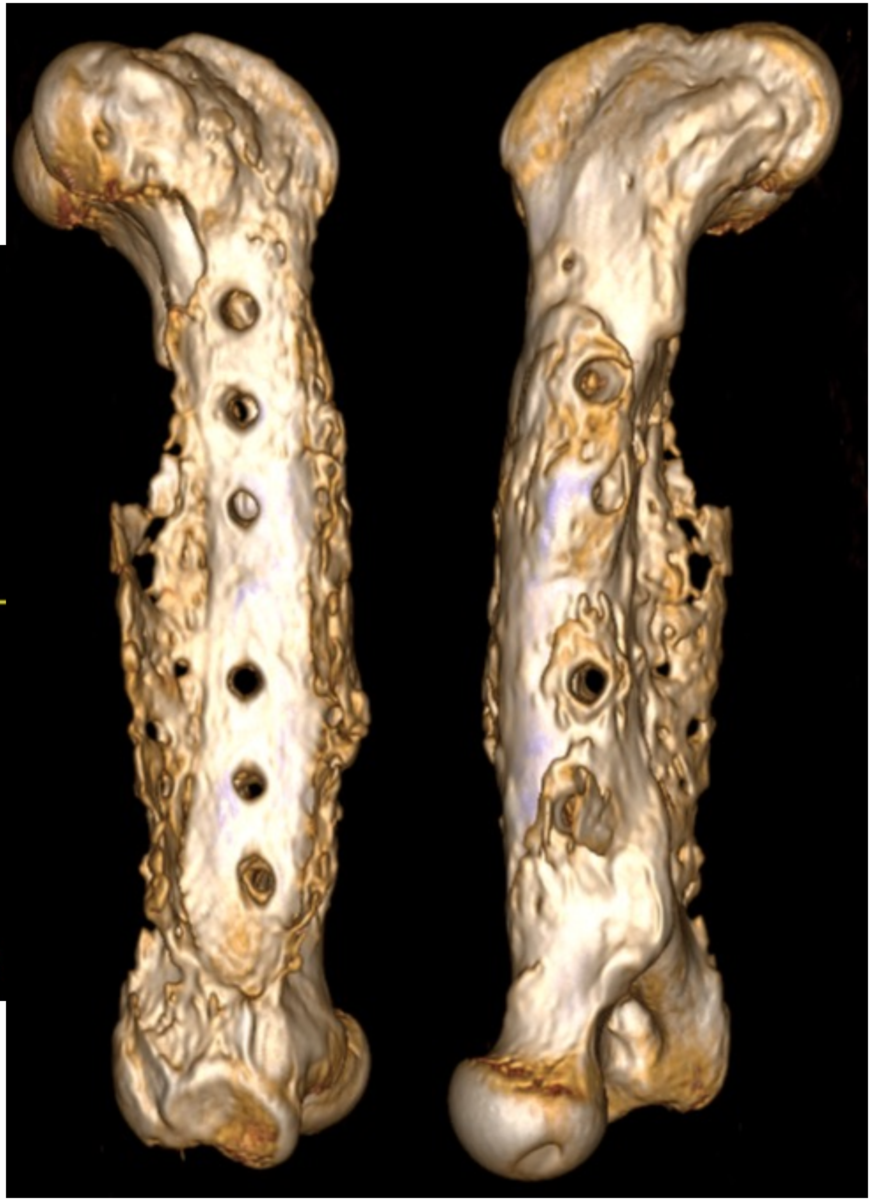

Infection Group / Infection and Uncoated Plate
Pig No. 28

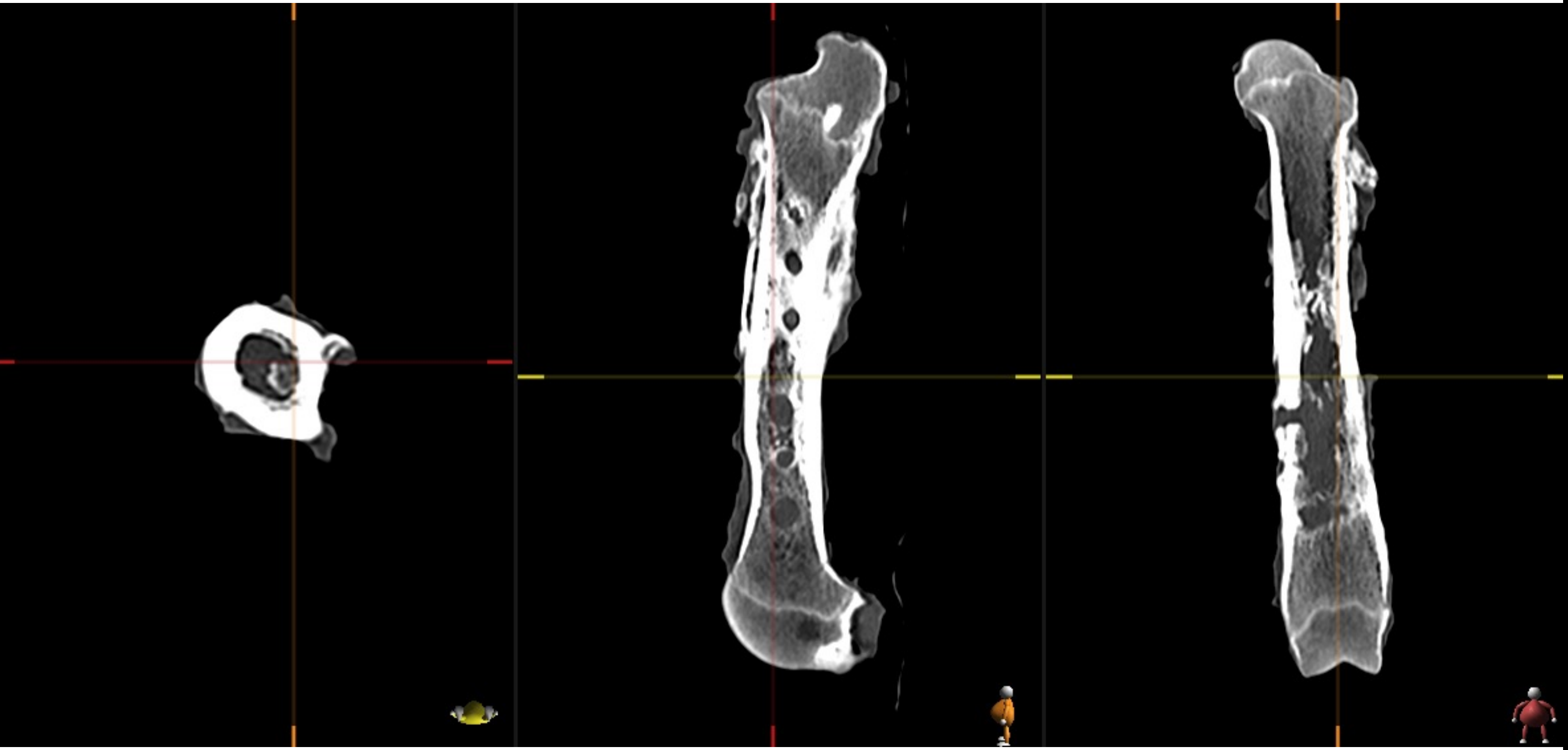

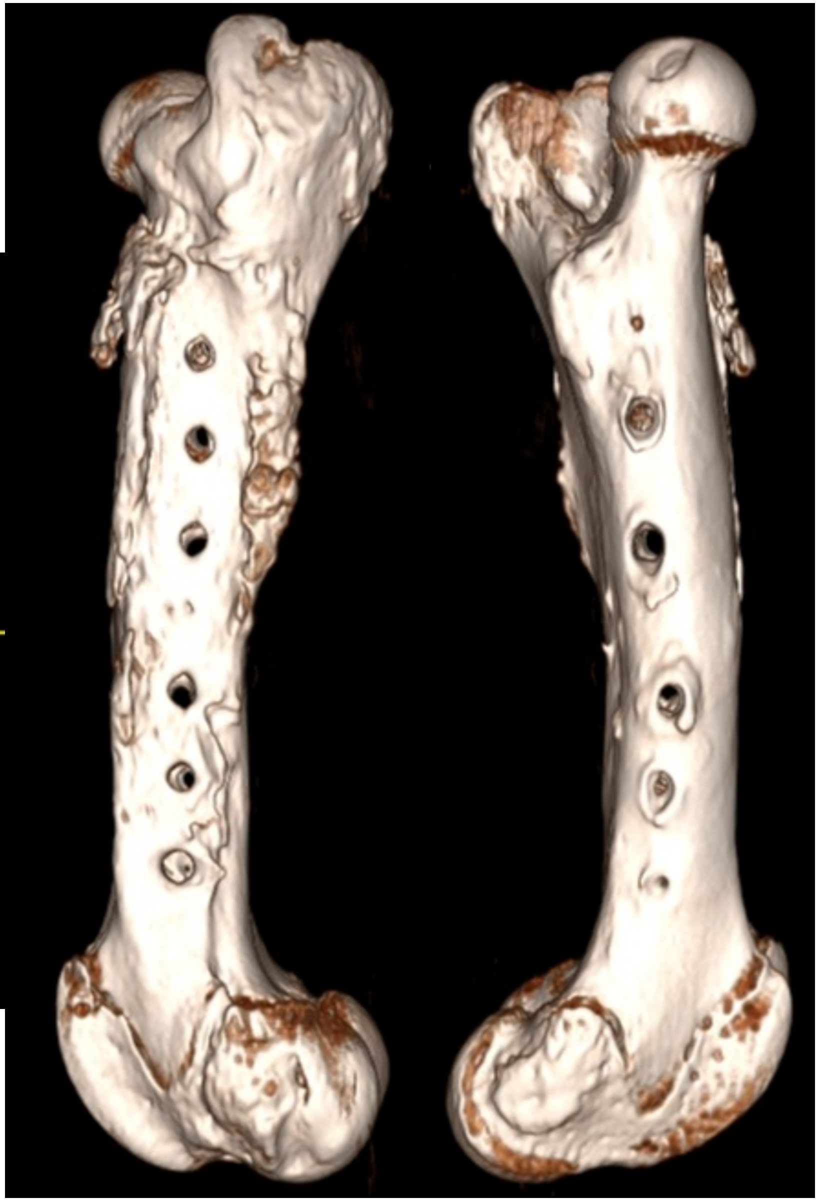

Infection Group / Infection and Uncoated Plate
Pig No. 29

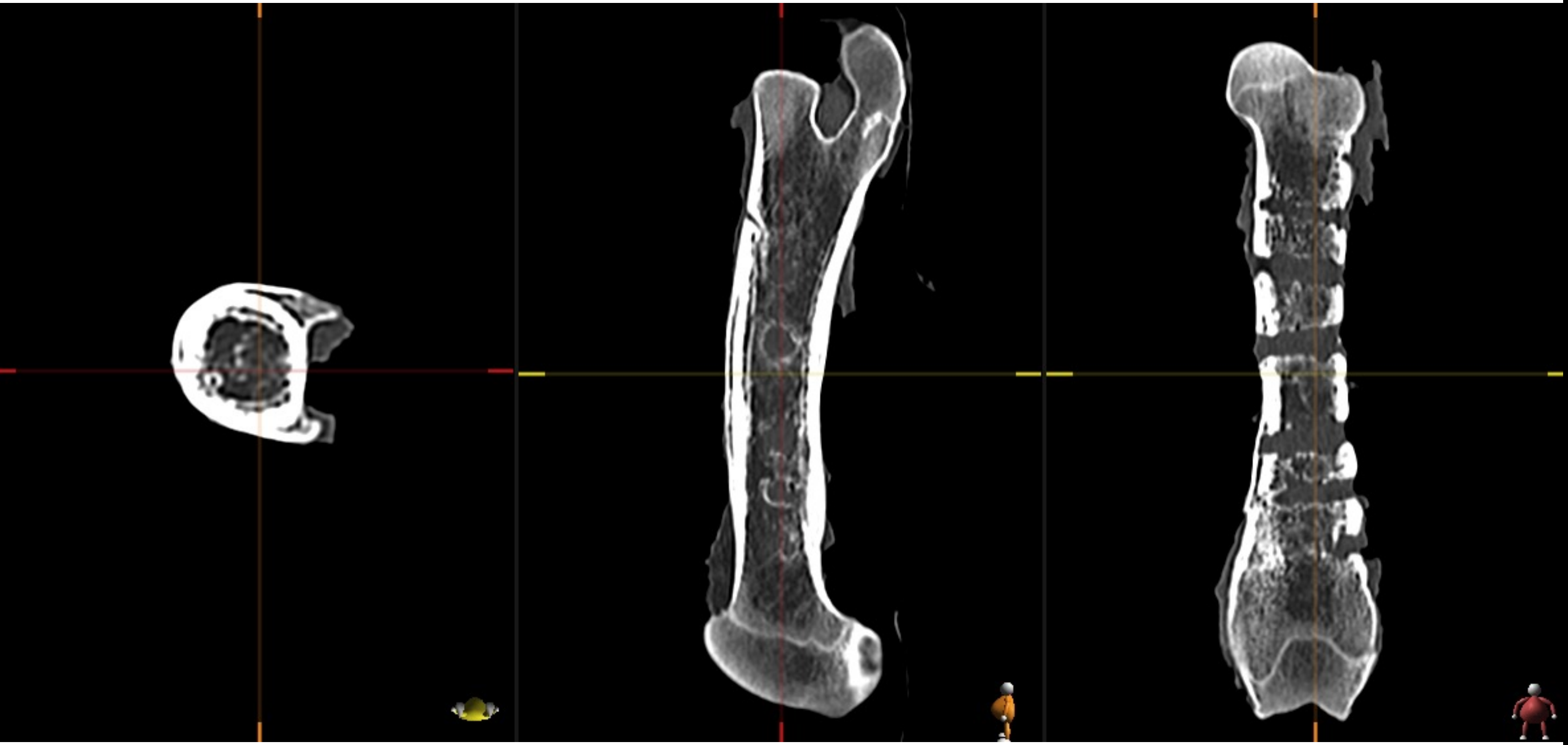

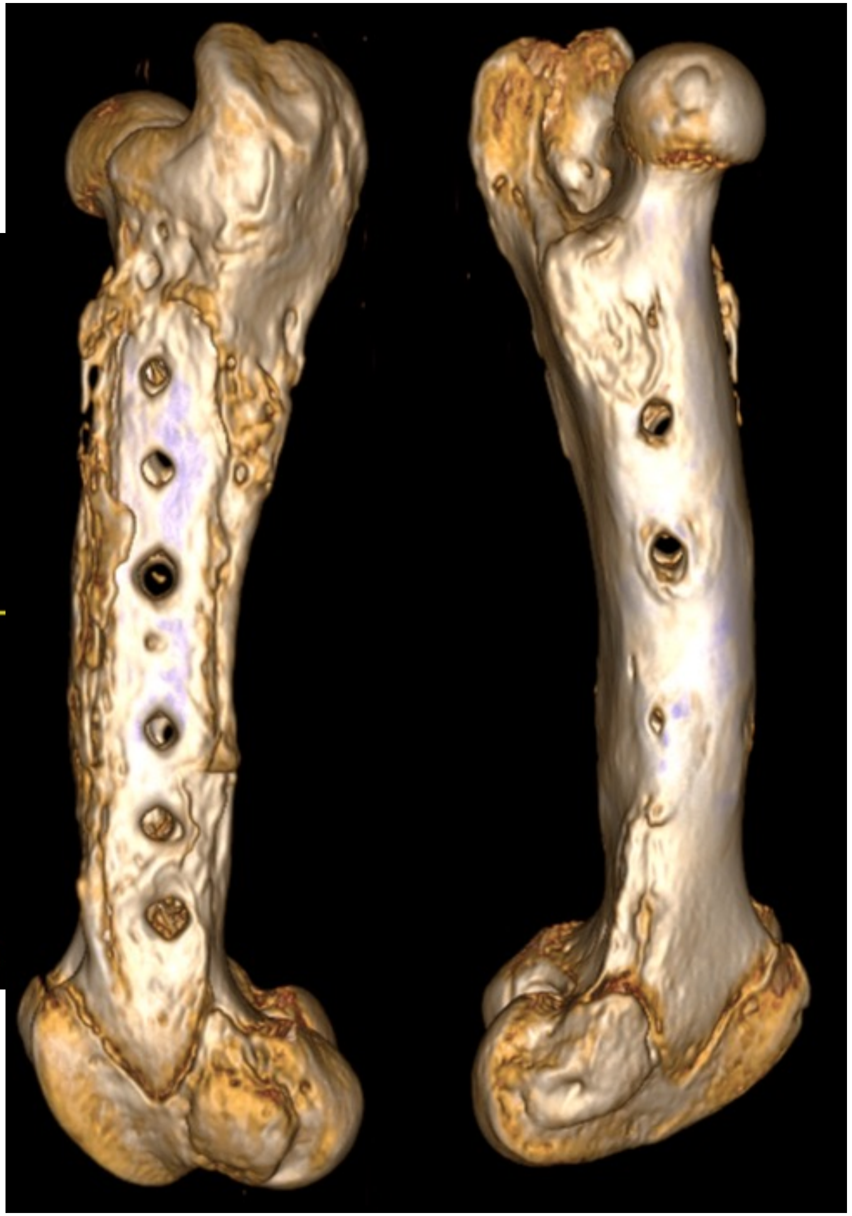

Infection Group / Infection and Uncoated Plate
Pig No. 30

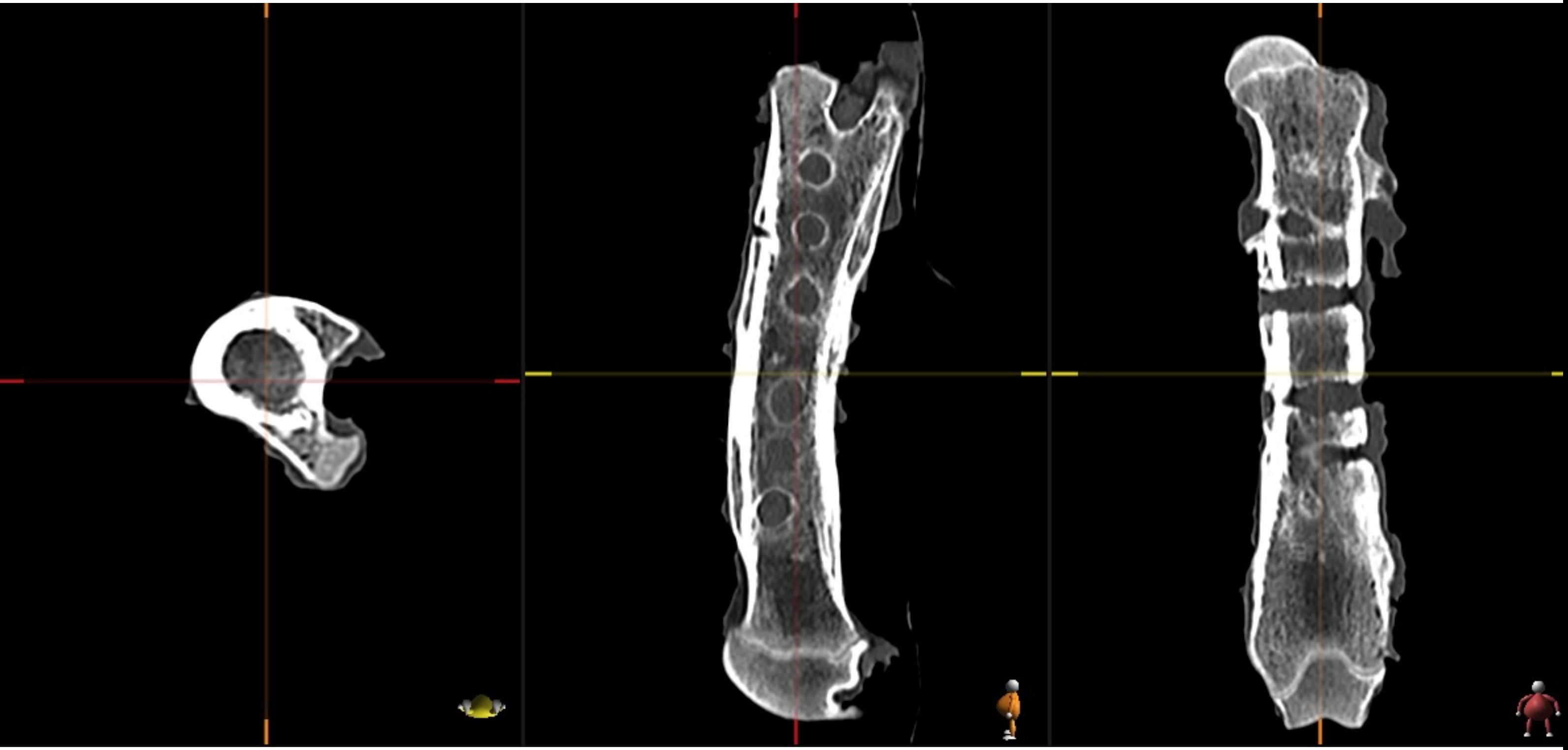

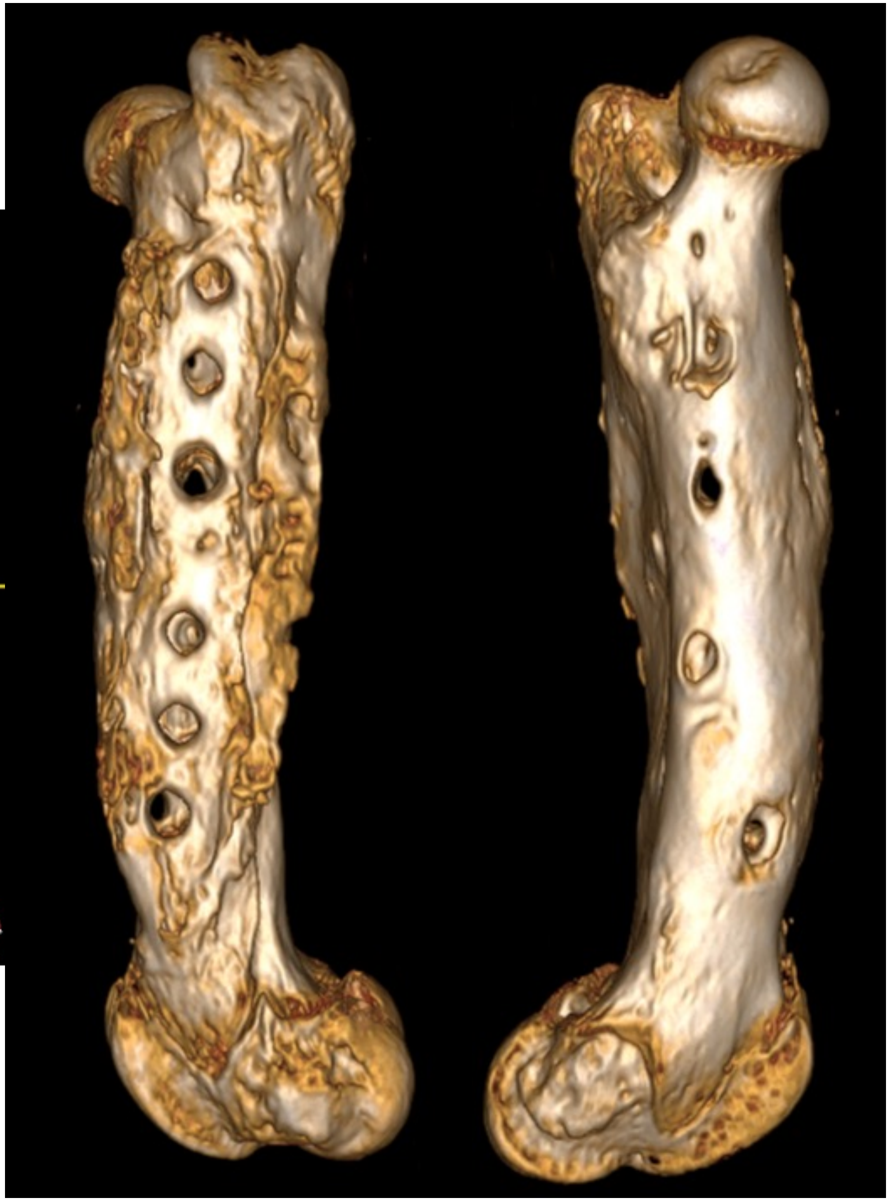

Infection Group / Infection and Uncoated Plate
Pig No. 31

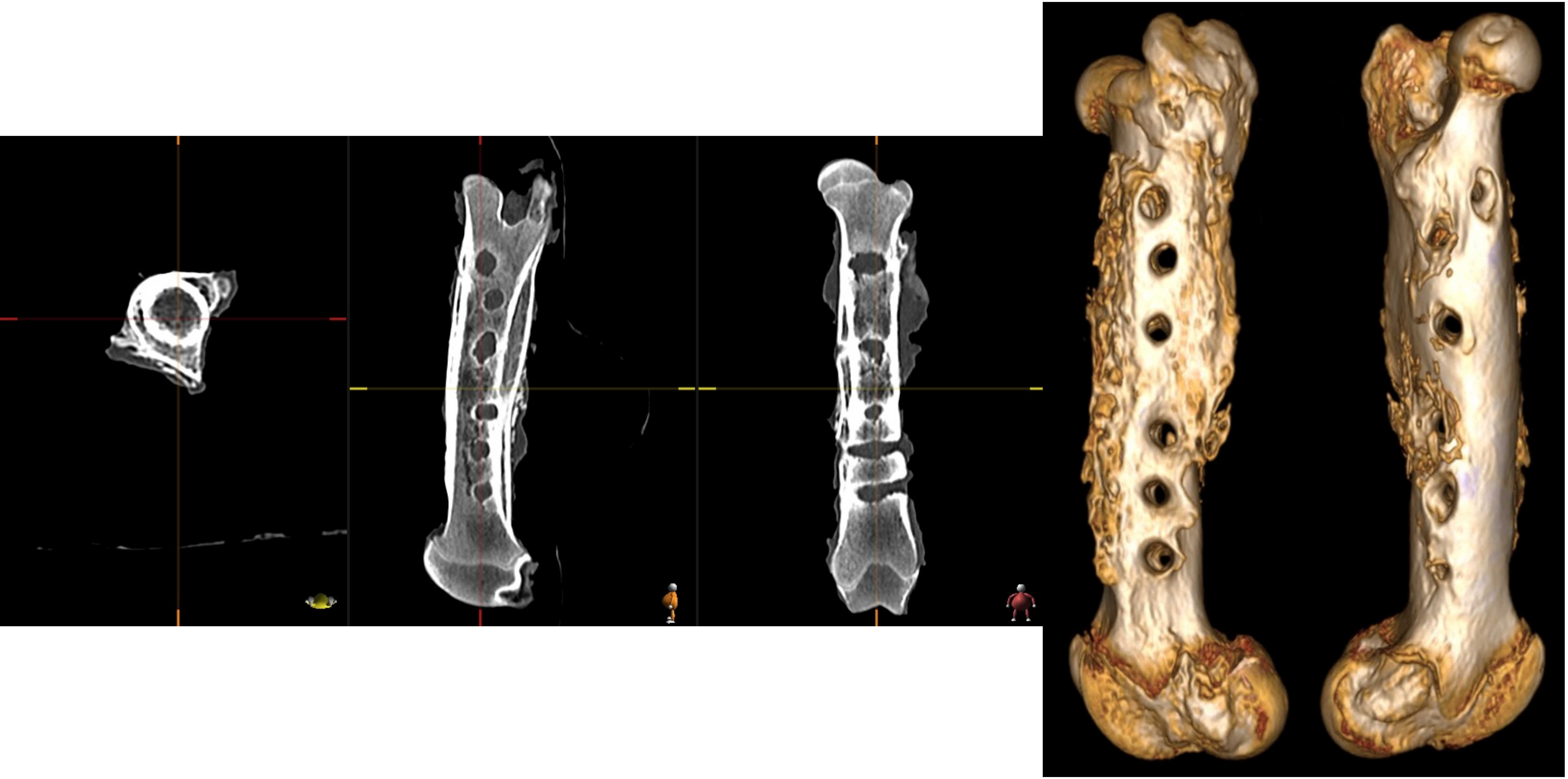

Infection Group / Infection and Uncoated Plate
Pig No. 32

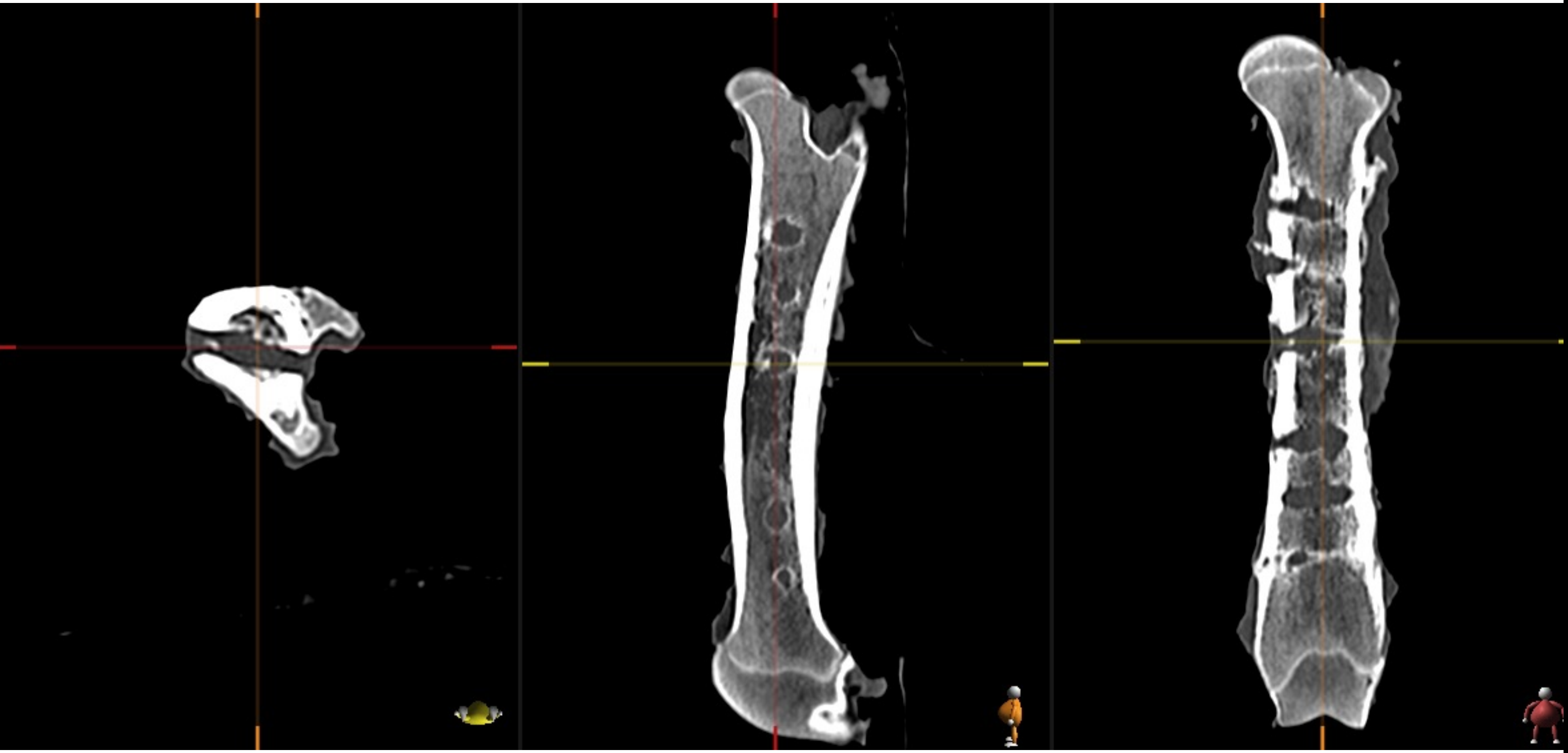

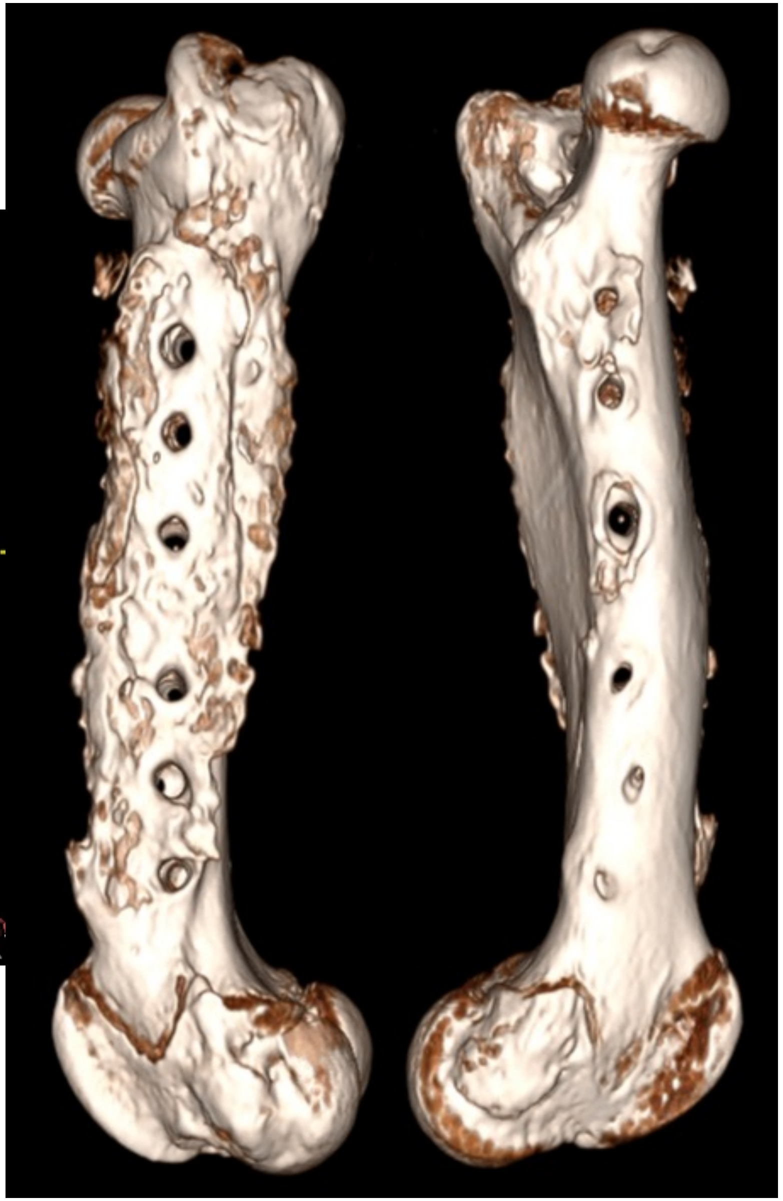

Lysostaphin Group / Infection and Coated Plate
Pig No. 18

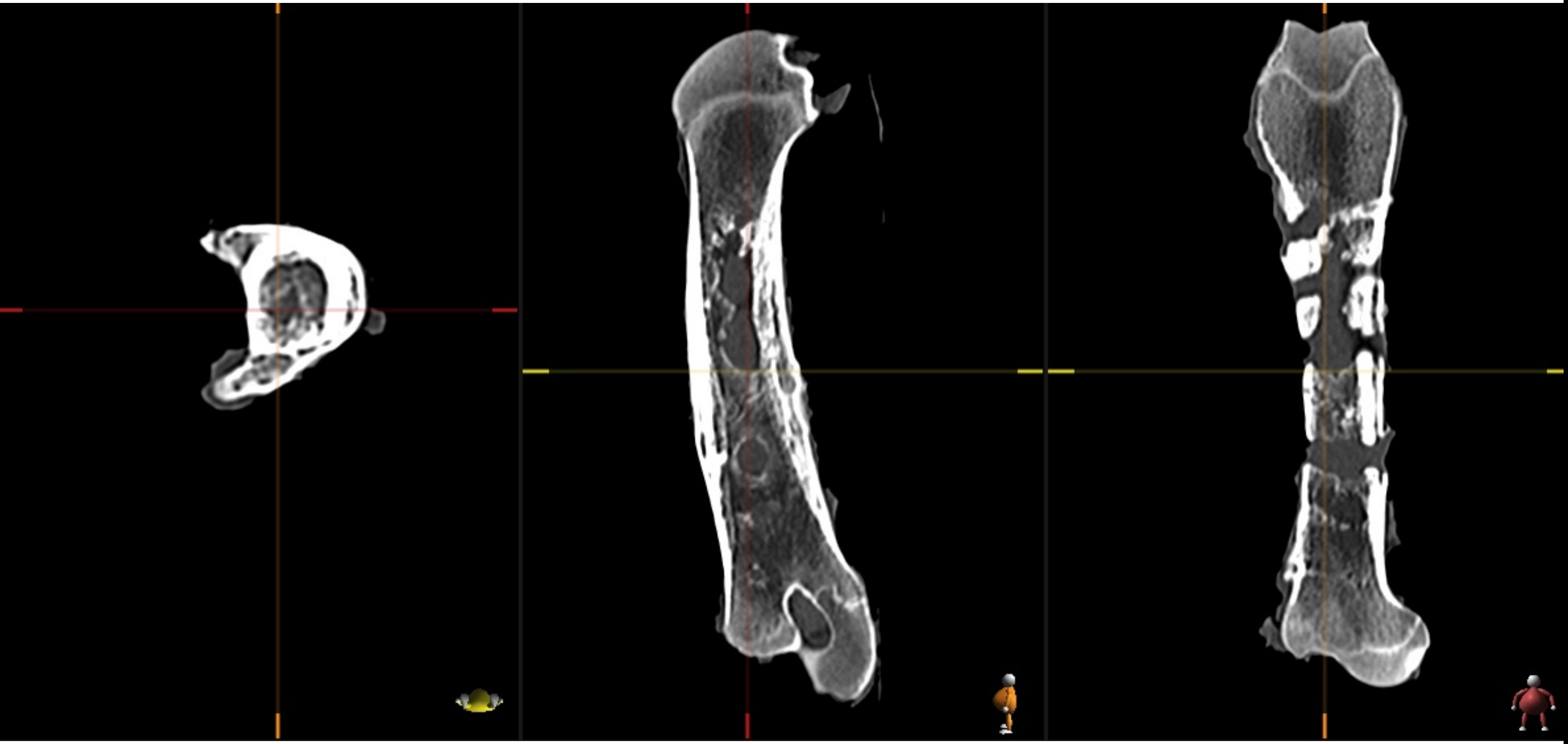

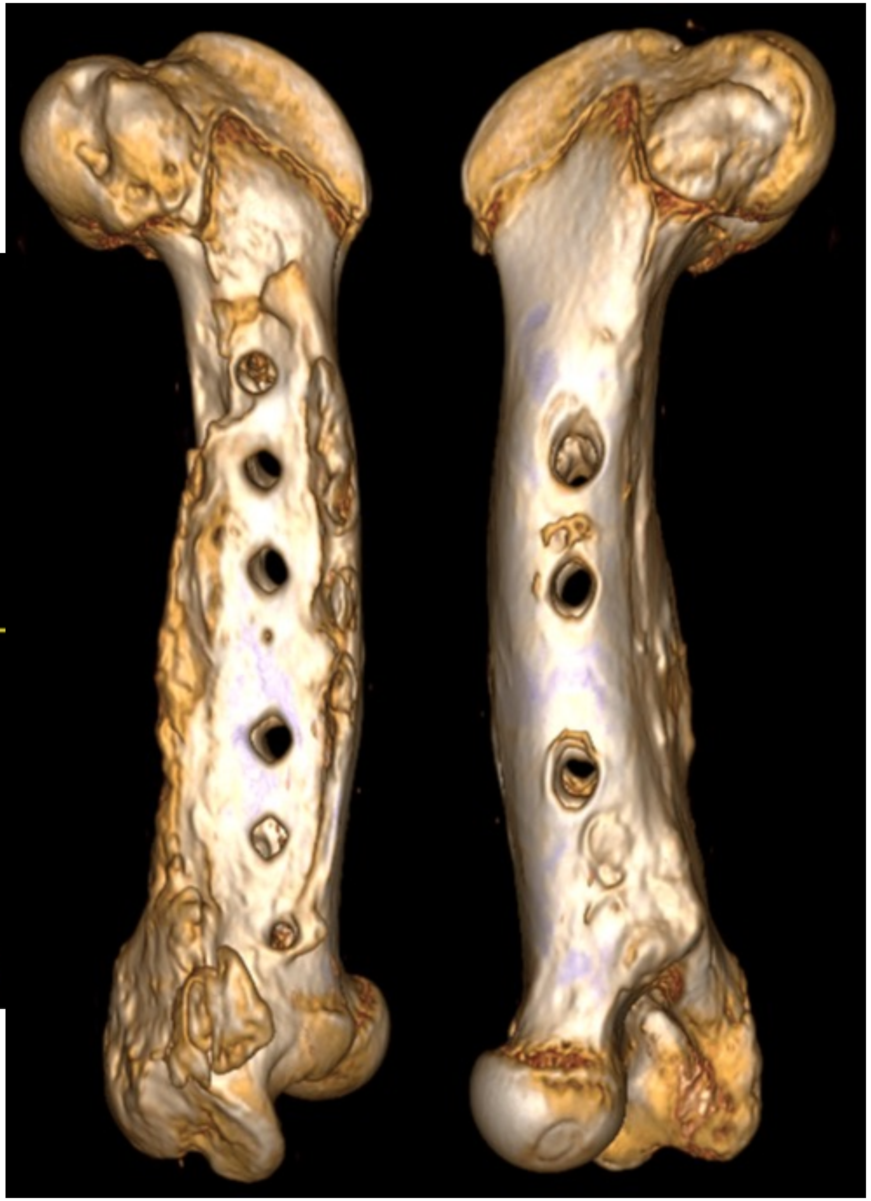

Lysostaphin Group / Infection and Coated Plate
Pig No. 19

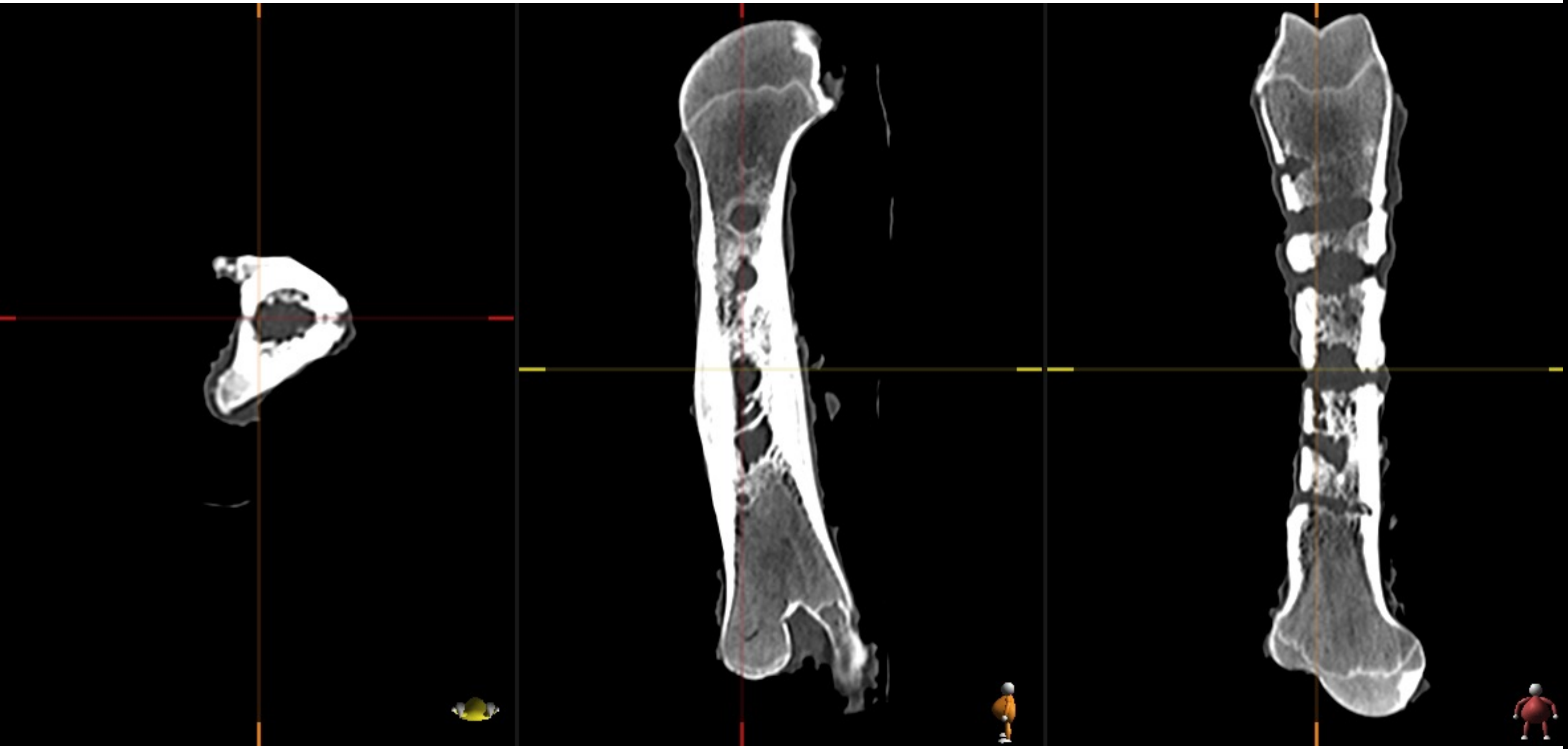

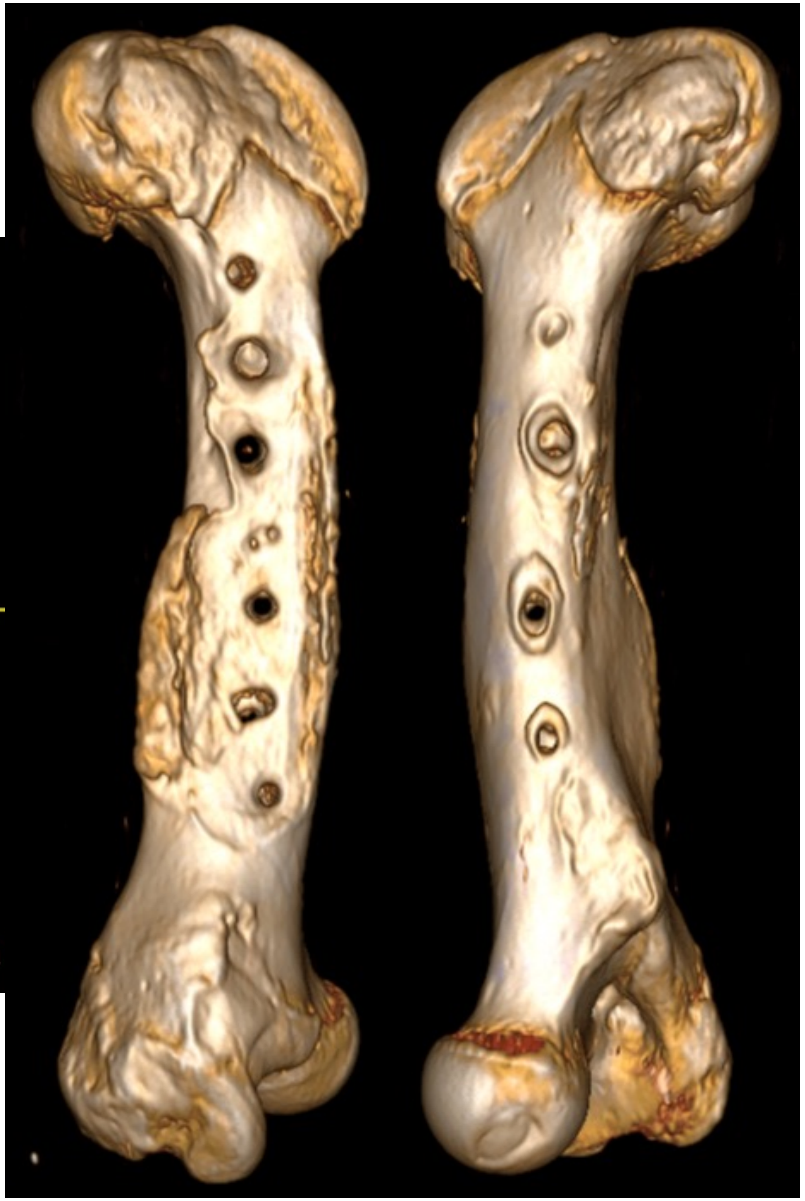

Lysostaphin Group / Infection and Coated Plate
Pig No. 20

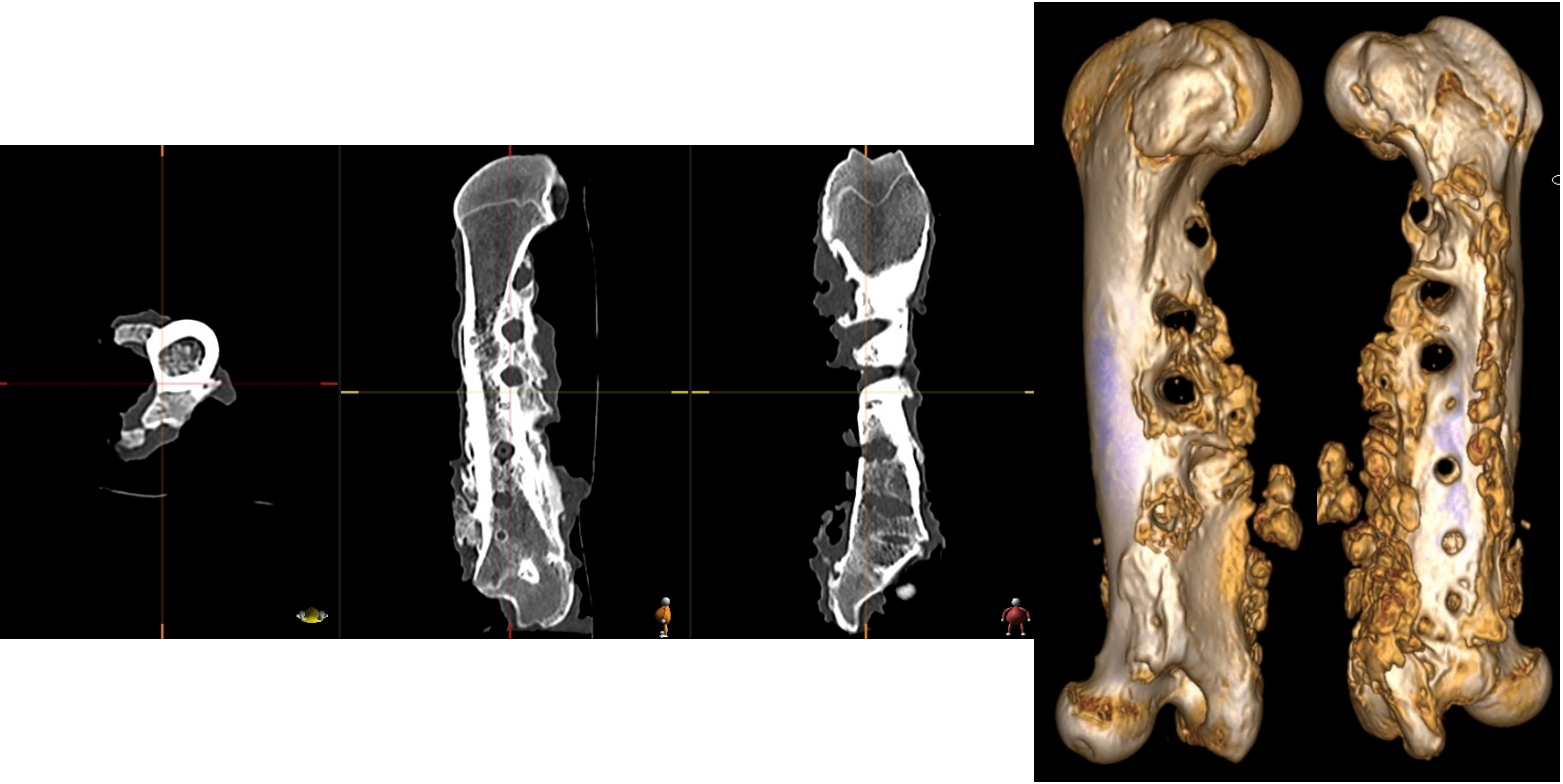

Lysostaphin Group / Infection and Coated Plate
Pig No. 21

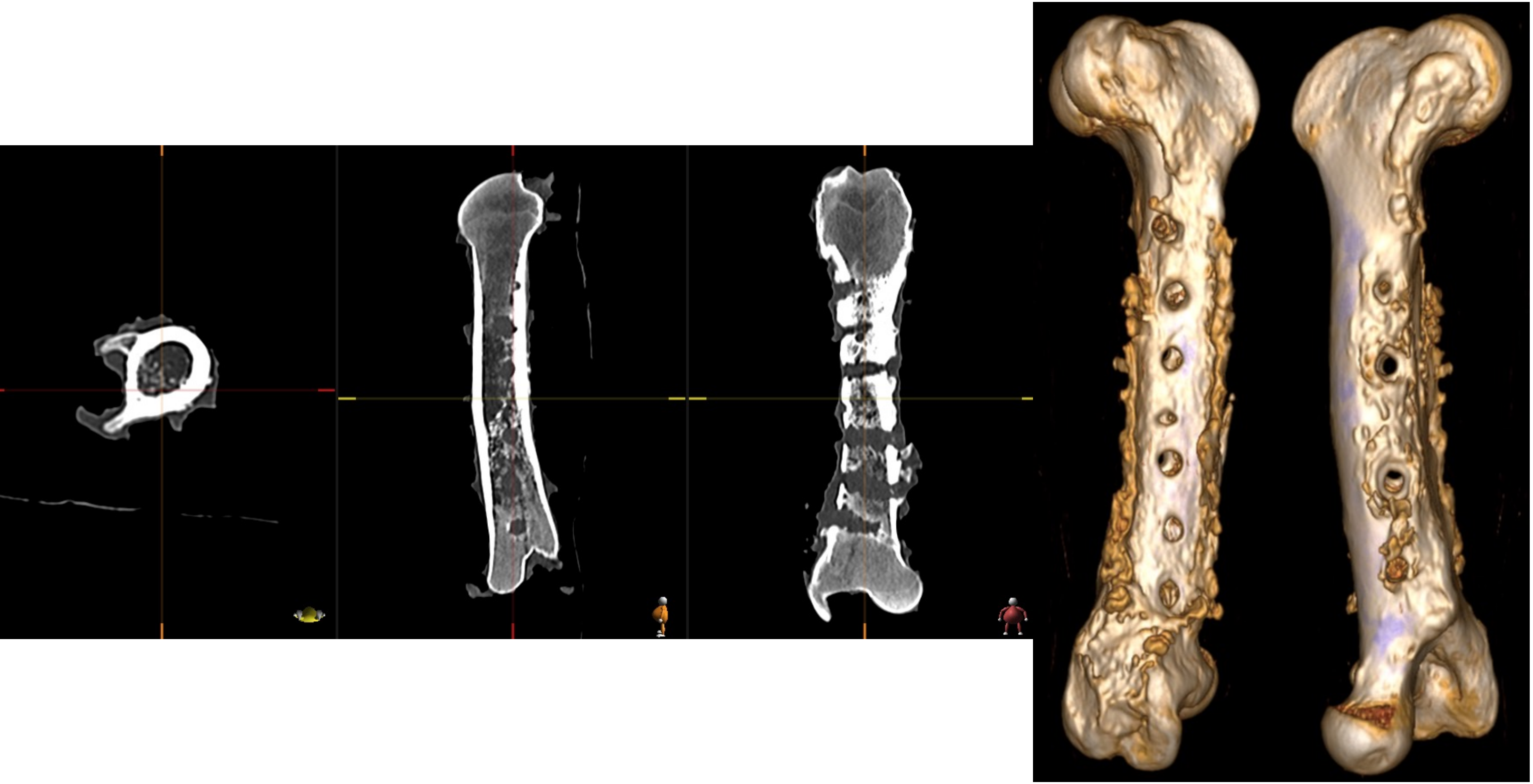

Lysostaphin Group / Infection and Coated Plate
Pig No. 22

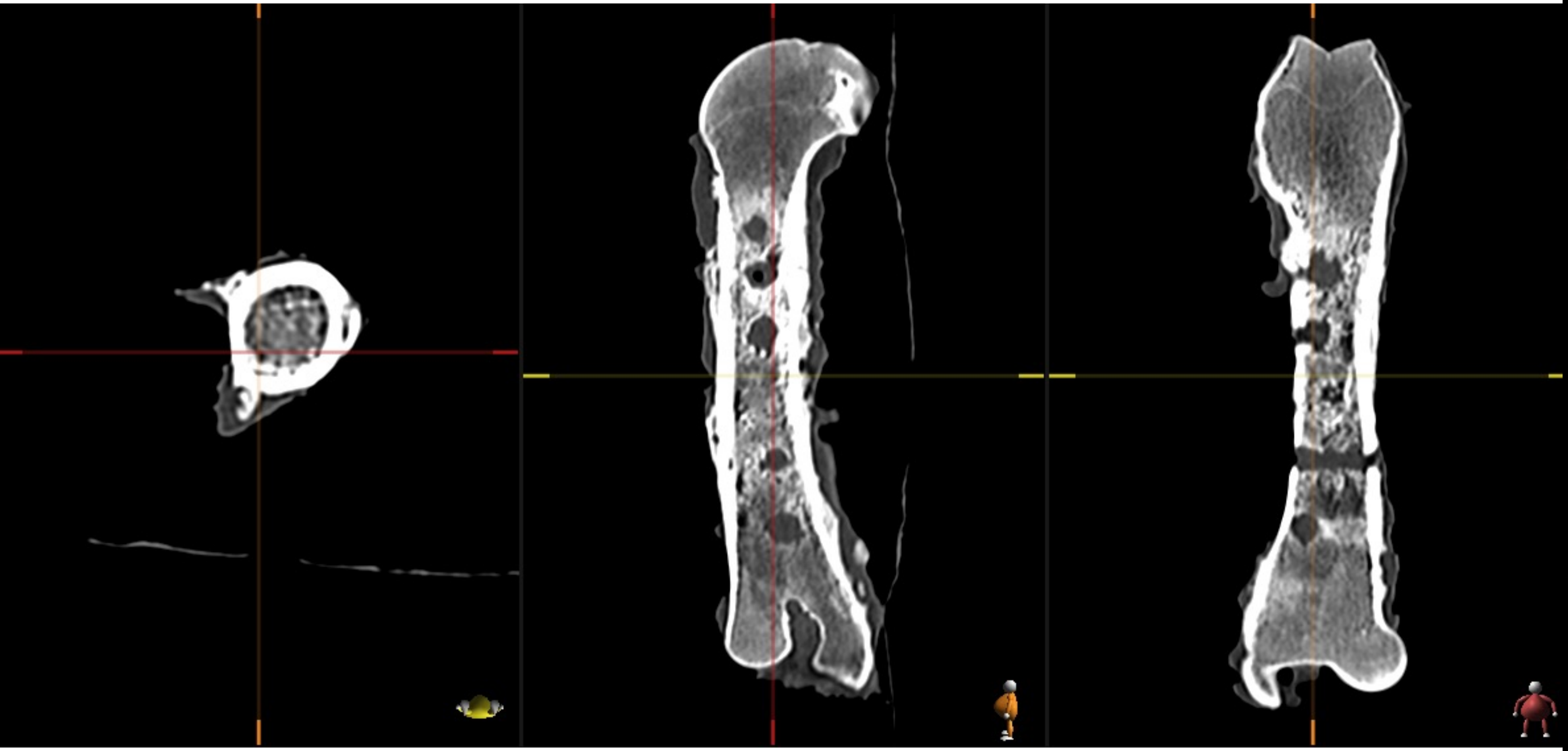

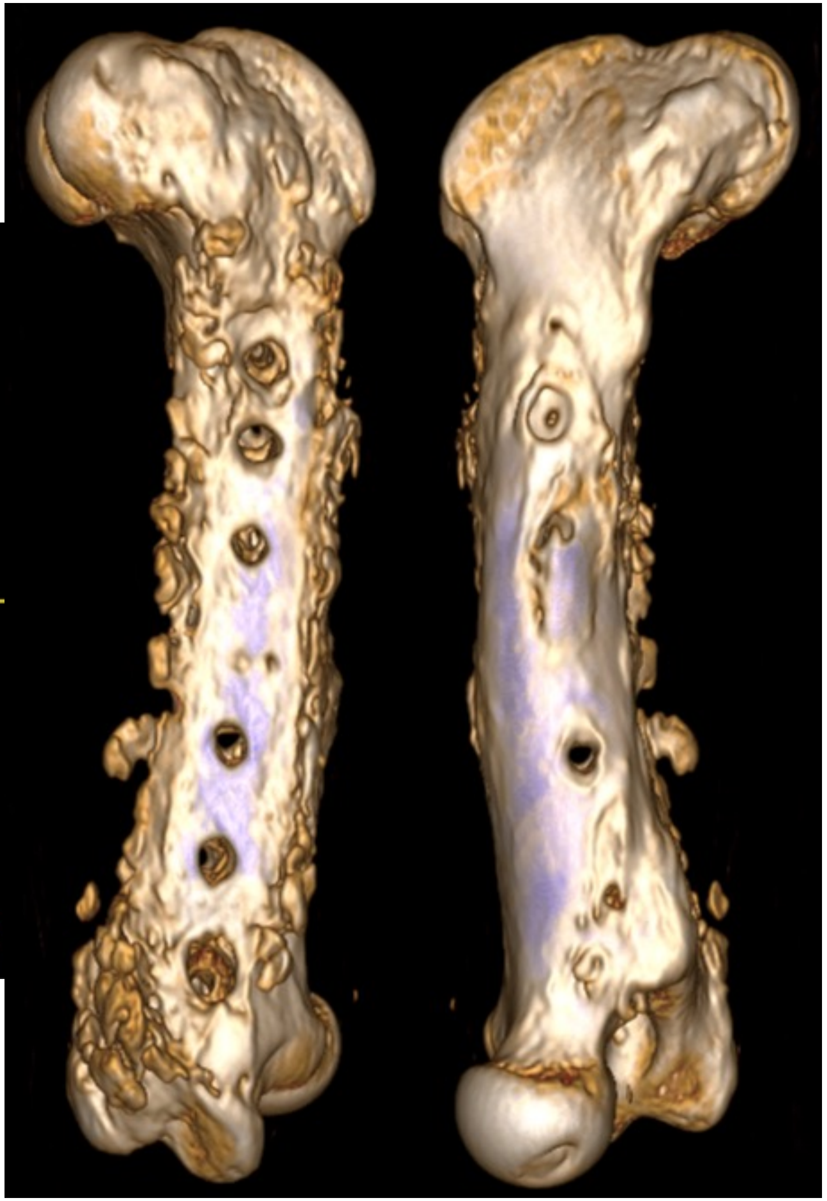

Lysostaphin Group / Infection and Coated Plate
Pig No. 23

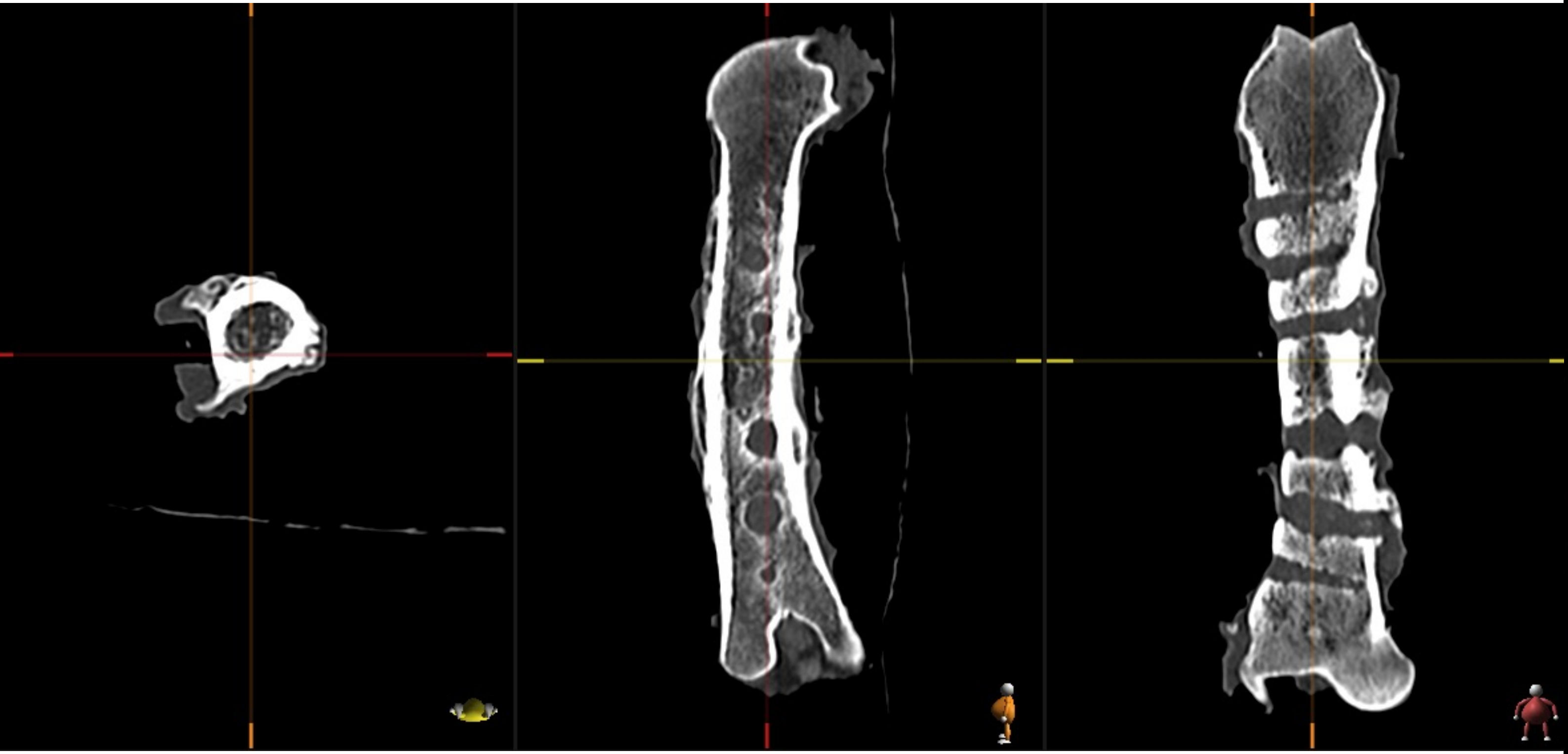

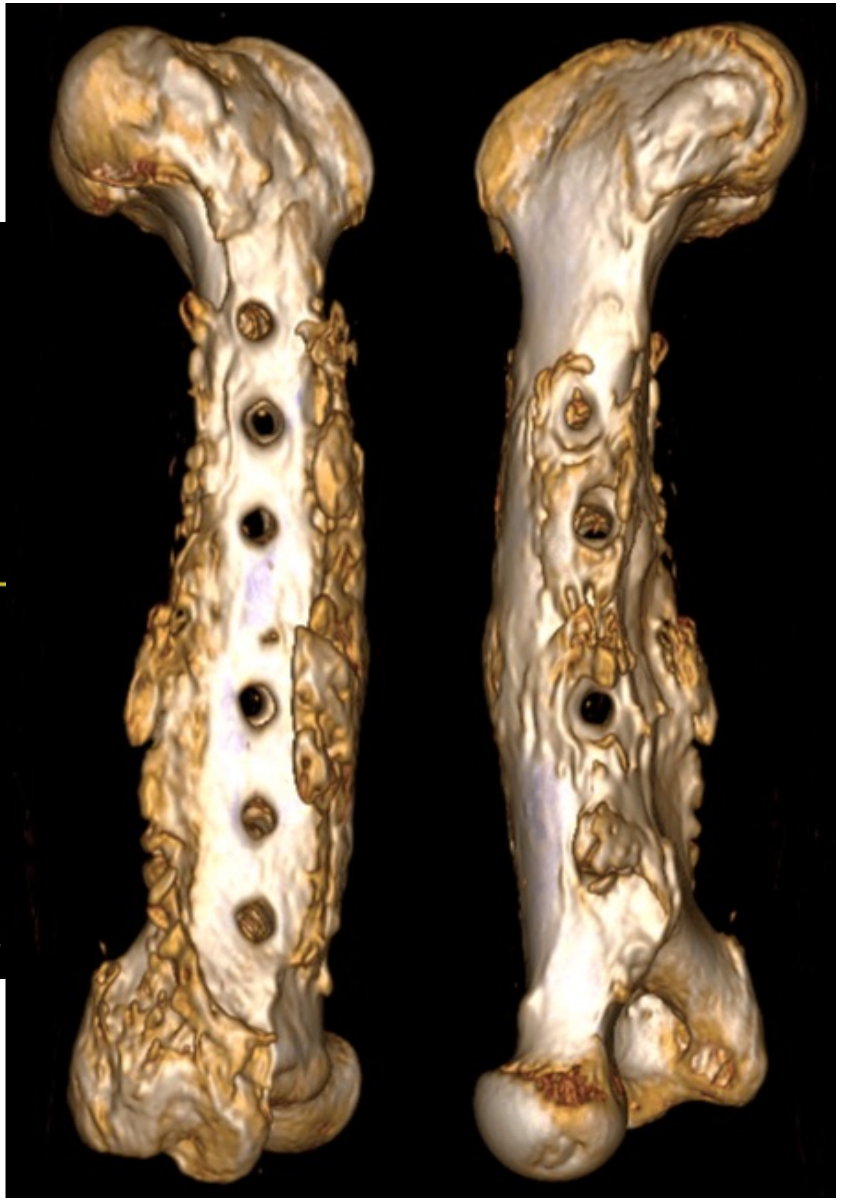

Lysostaphin Group / Infection and Coated Plate
Pig No. 24

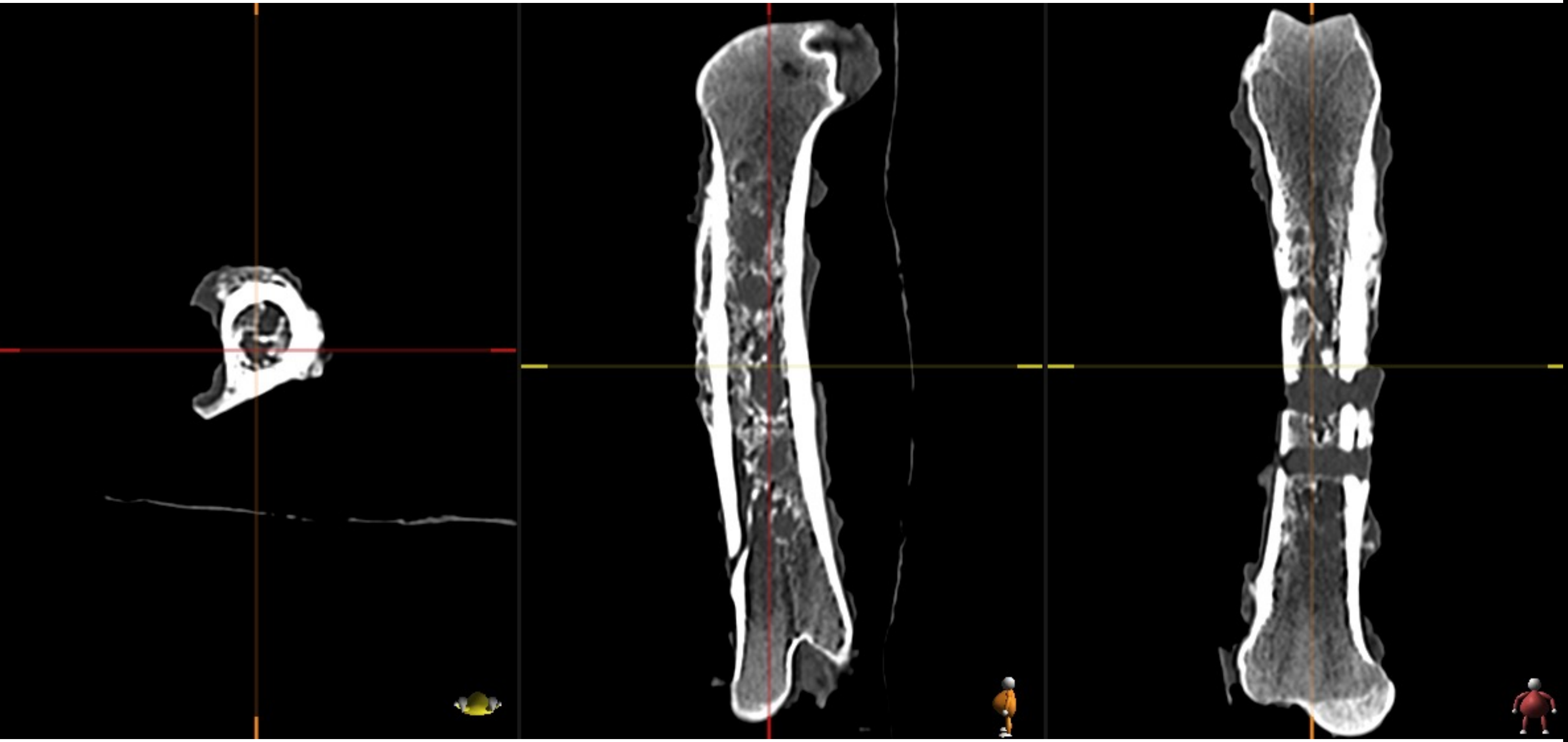

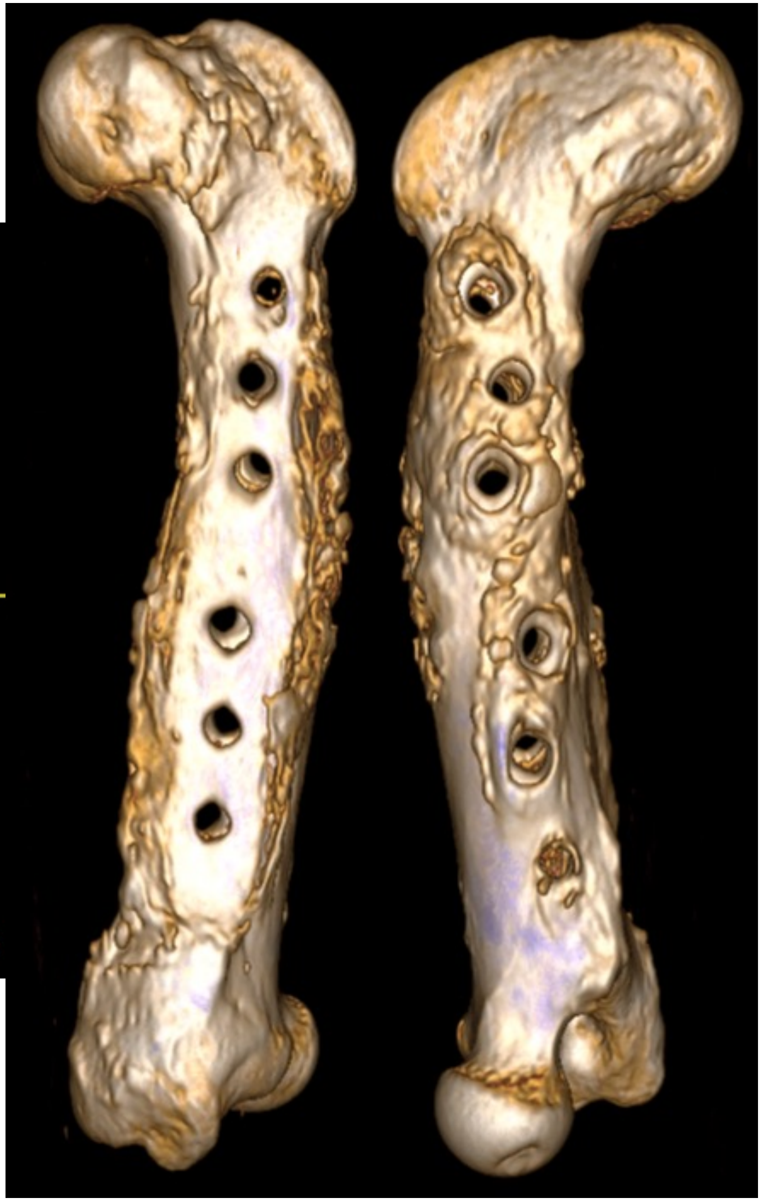

Lysostaphin Group / Infection and Coated Plate
Pig No. 25

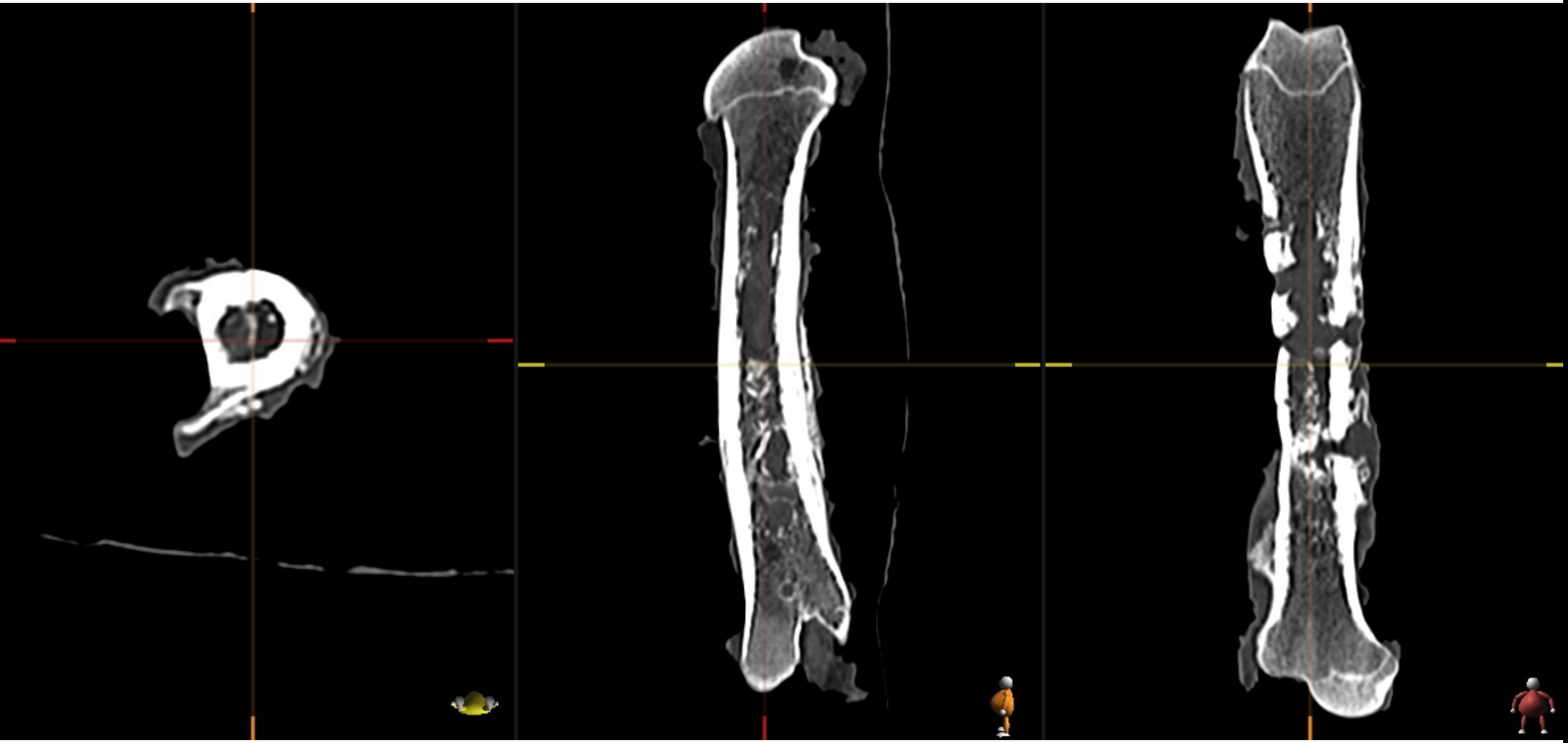

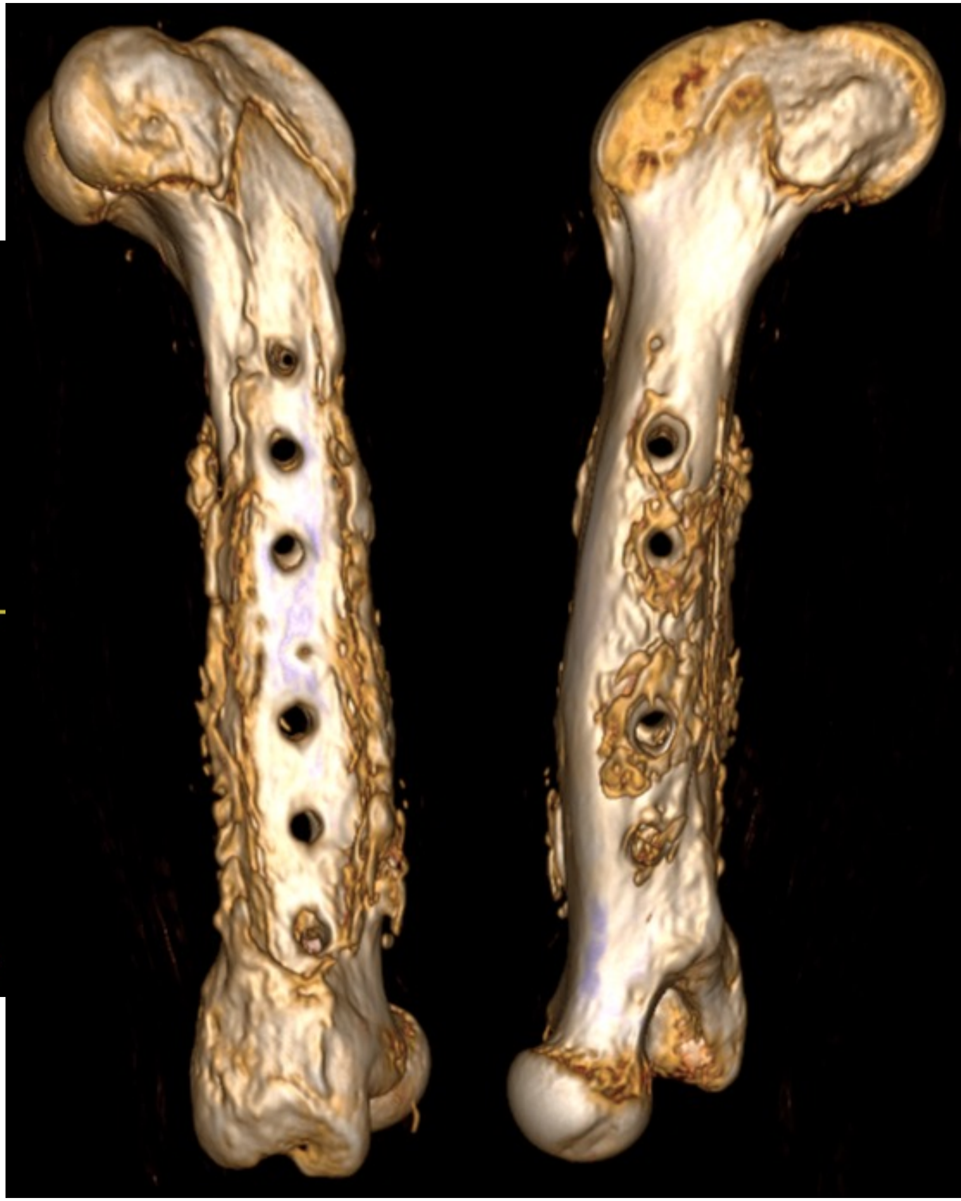

Lysostaphin Group / Infection and Coated Plate
Pig No. 26

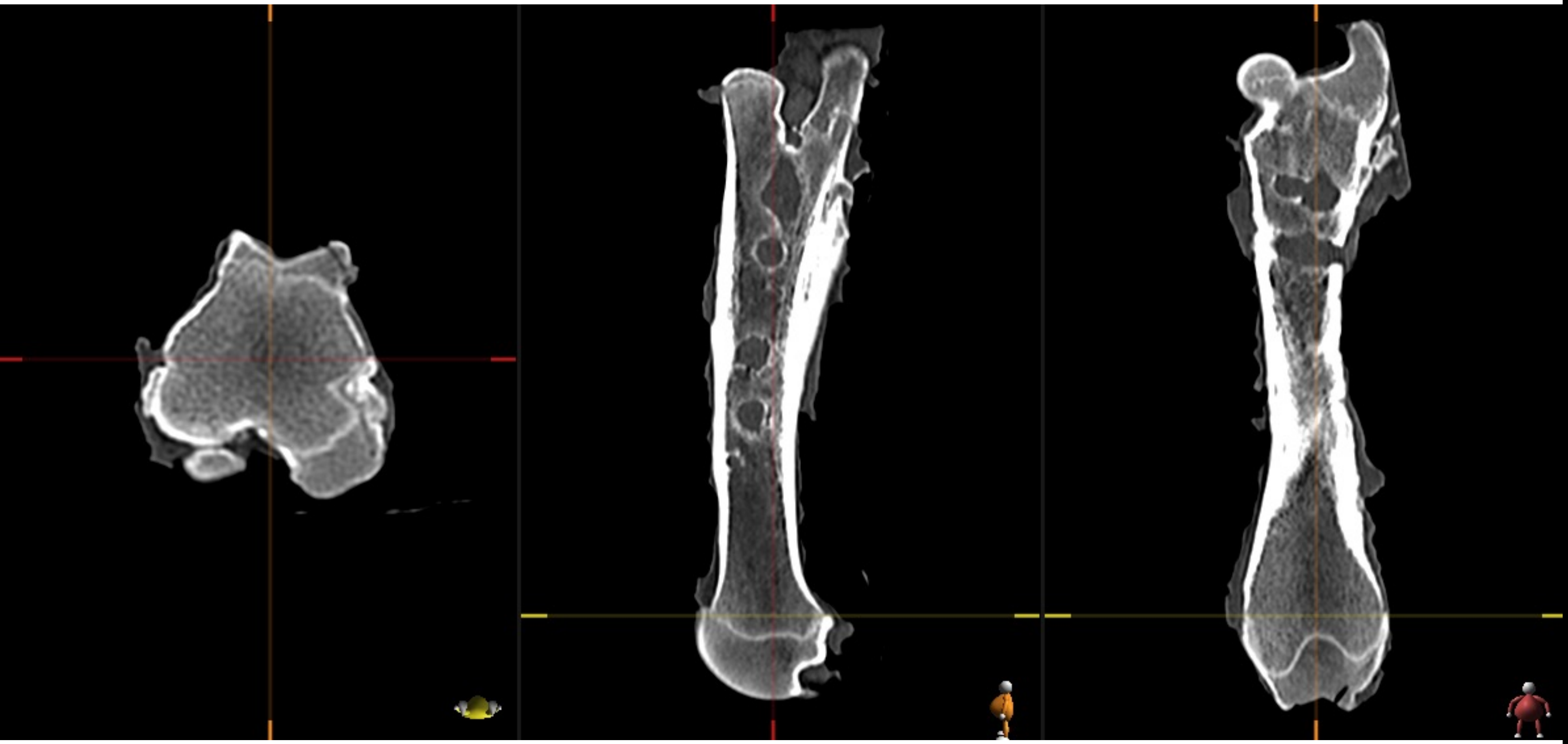

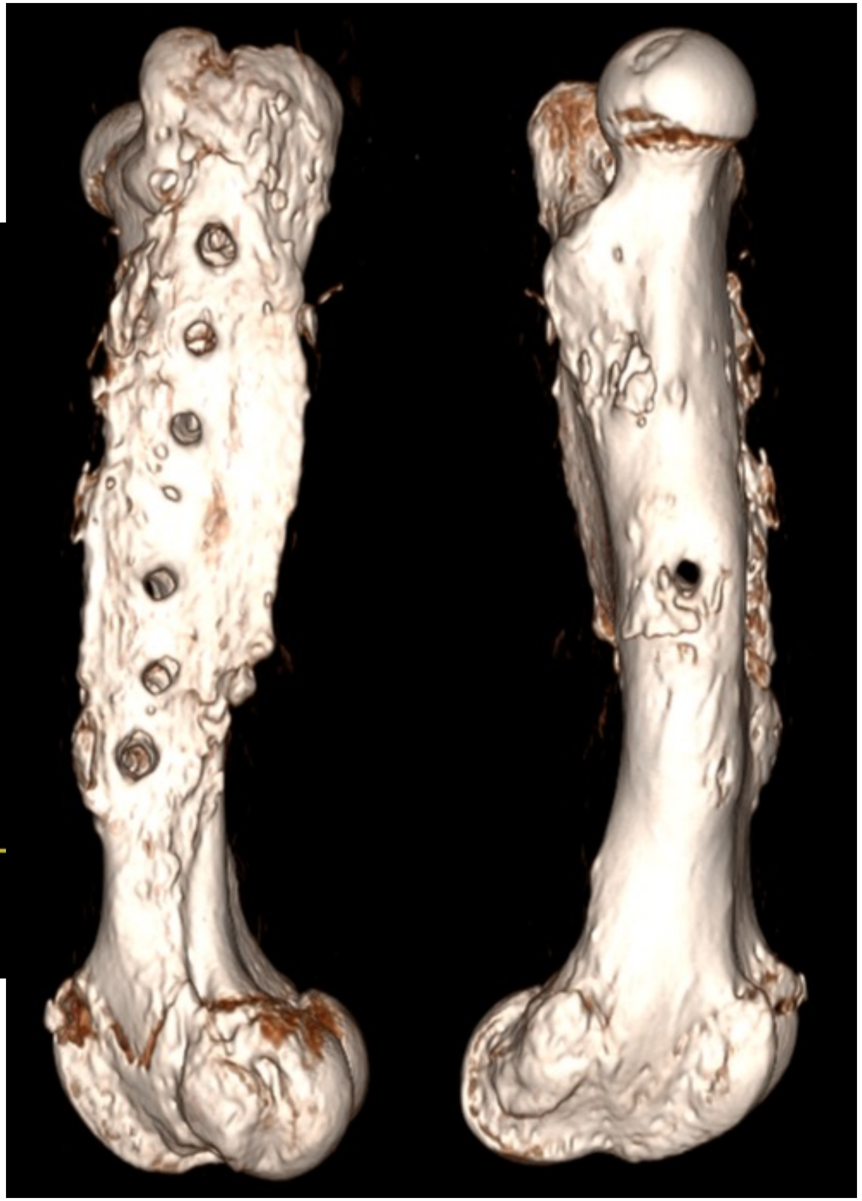

Lysostaphin Group / Infection and Coated Plate
Pig No. 27

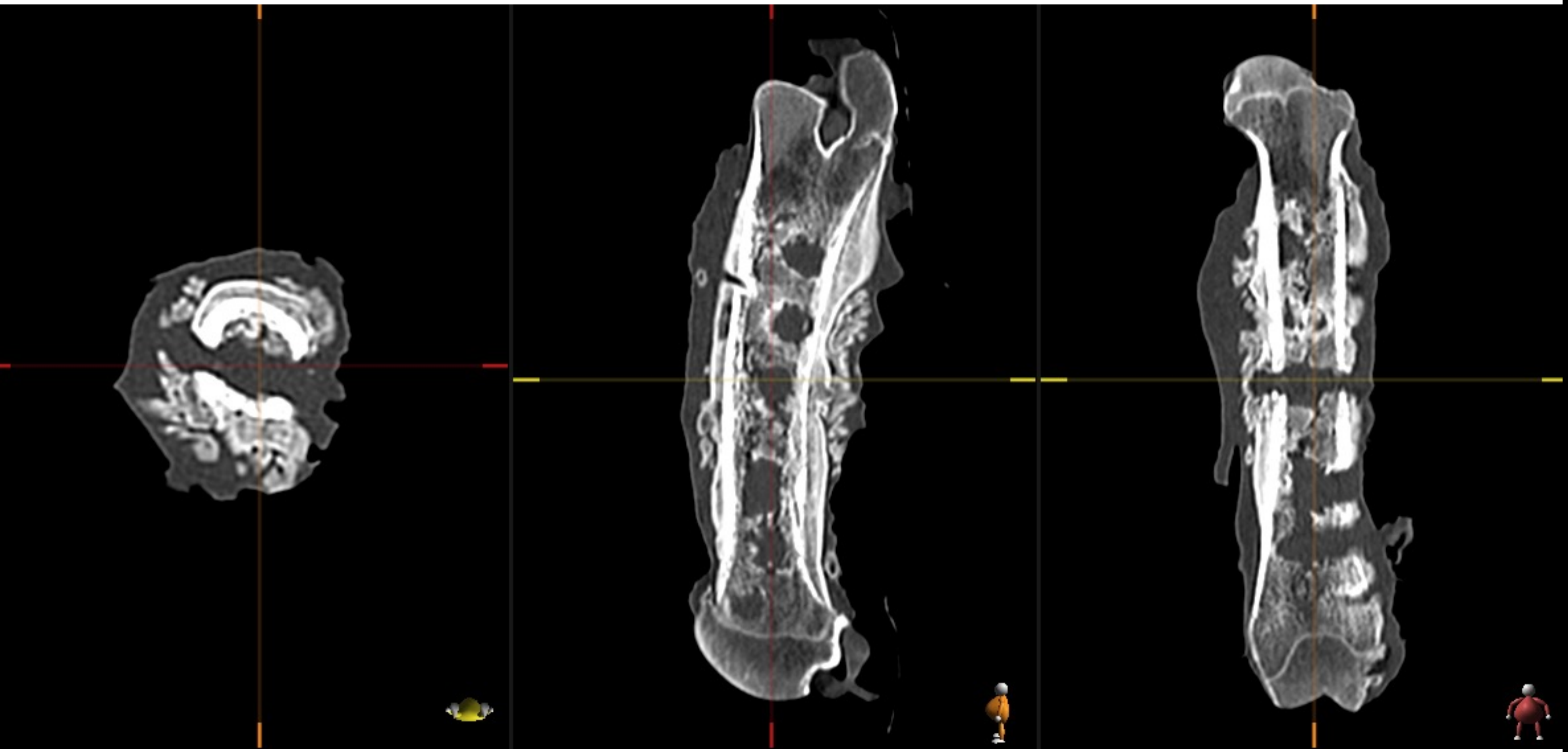

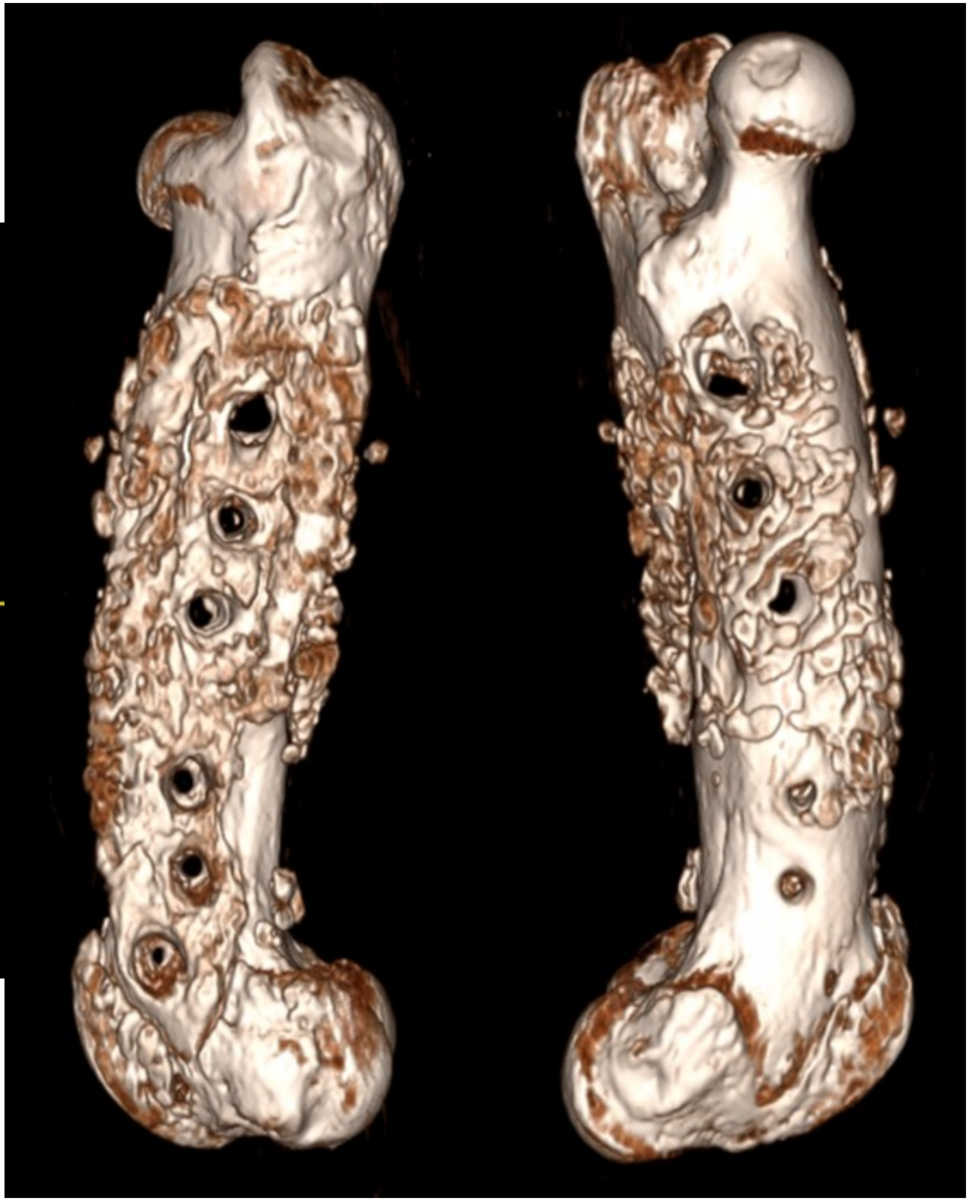

Supplement: Supplementary file 1 — (PDF 4043 kb) [file 68_2024_2448_MOESM1_ESM.pdf]
