# Supplementary figures and images for: Efficacy of lysostaphin-coated titanium plates on implant-associated MRSA osteitis in minipigs
Source: Eur J Trauma Emerg Surg. 2024 Jan 24;50(3):887–95. doi: 10.1007/s00068-024-02448-4 (PMC11249774; doi:10.1007/s00068-024-02448-4)

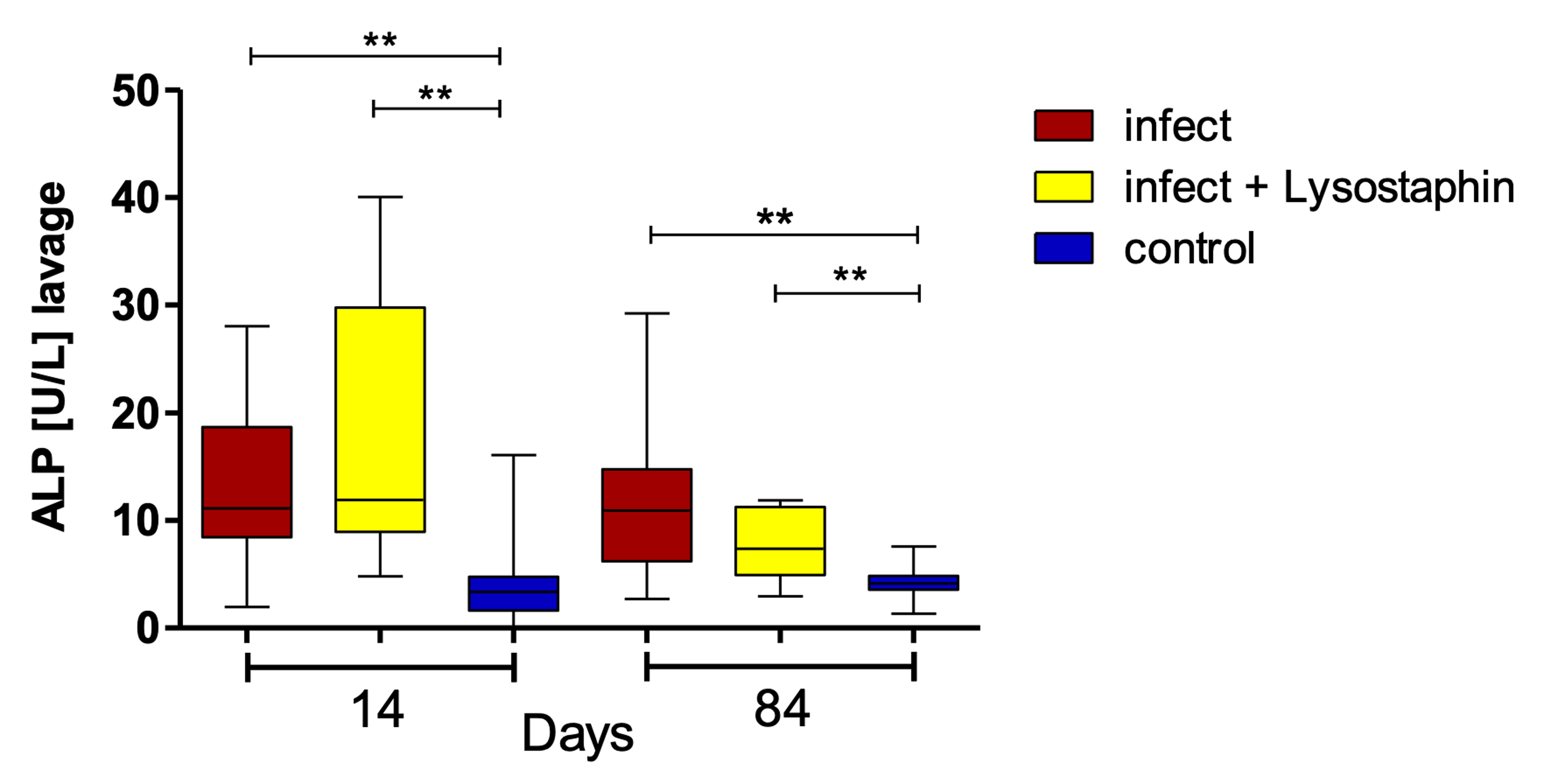

Supplement: Supplementary file 2 — (PNG 212 kb) [file 68_2024_2448_Fig8_ESM.png]

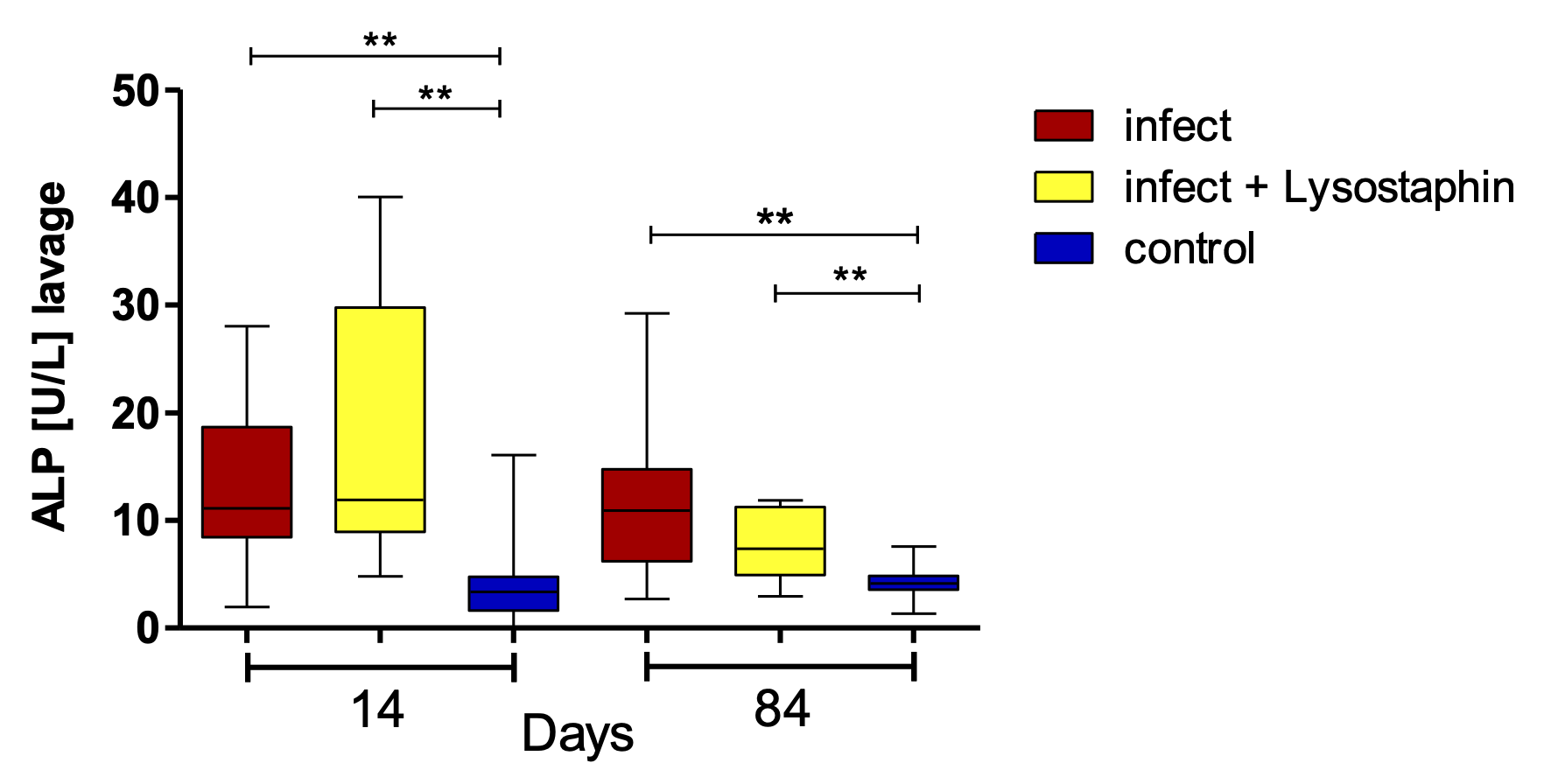

Supplement: Supplementary file 3 — High resolution image (TIF 101 kb) [file 68_2024_2448_MOESM2_ESM.tif]

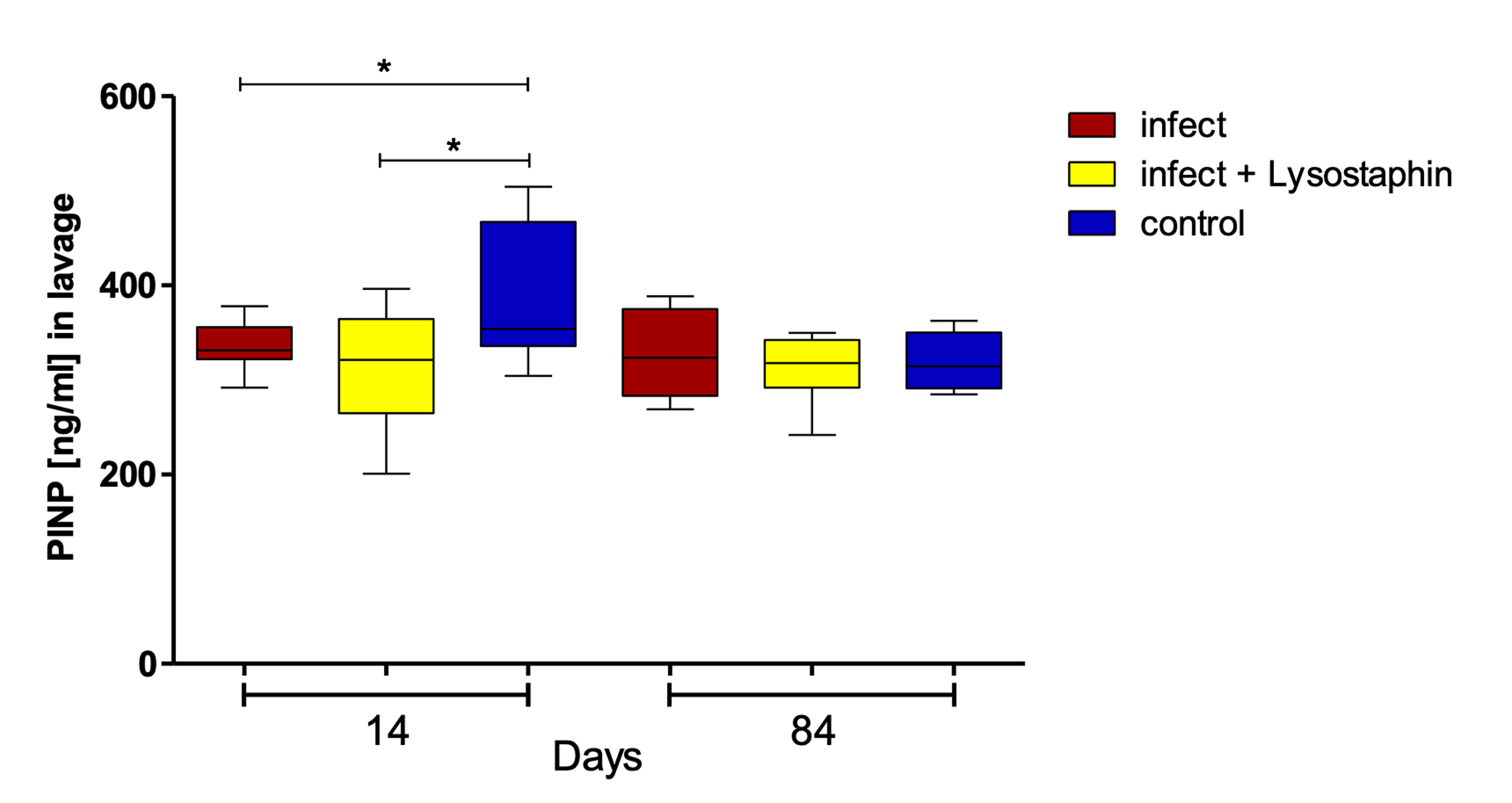

Supplement: Supplementary file 4 — (PNG 179 kb) [file 68_2024_2448_Fig9_ESM.png]

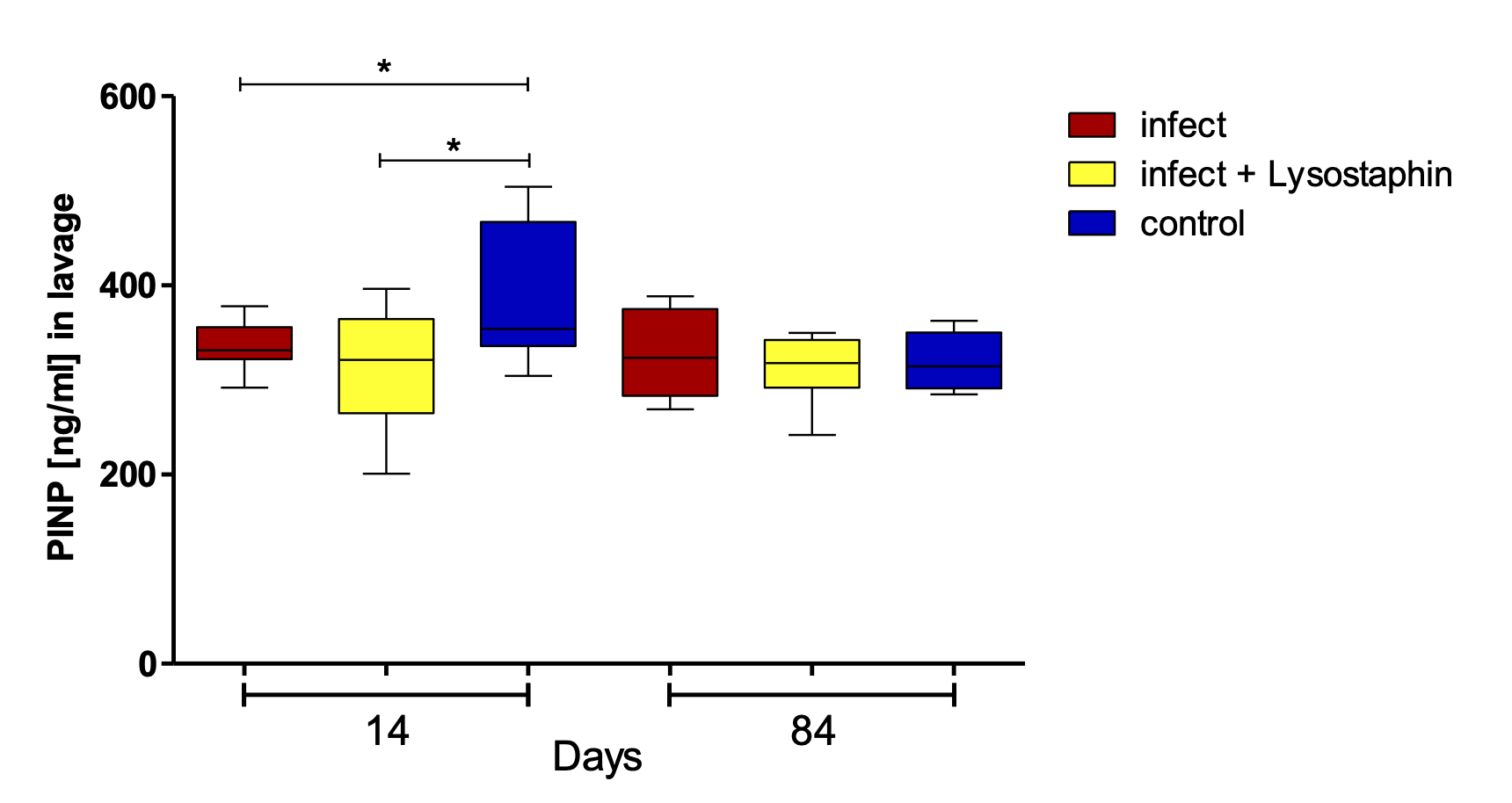

Supplement: Supplementary file 5 — High resolution image (TIF 86 kb) [file 68_2024_2448_MOESM3_ESM.tif]

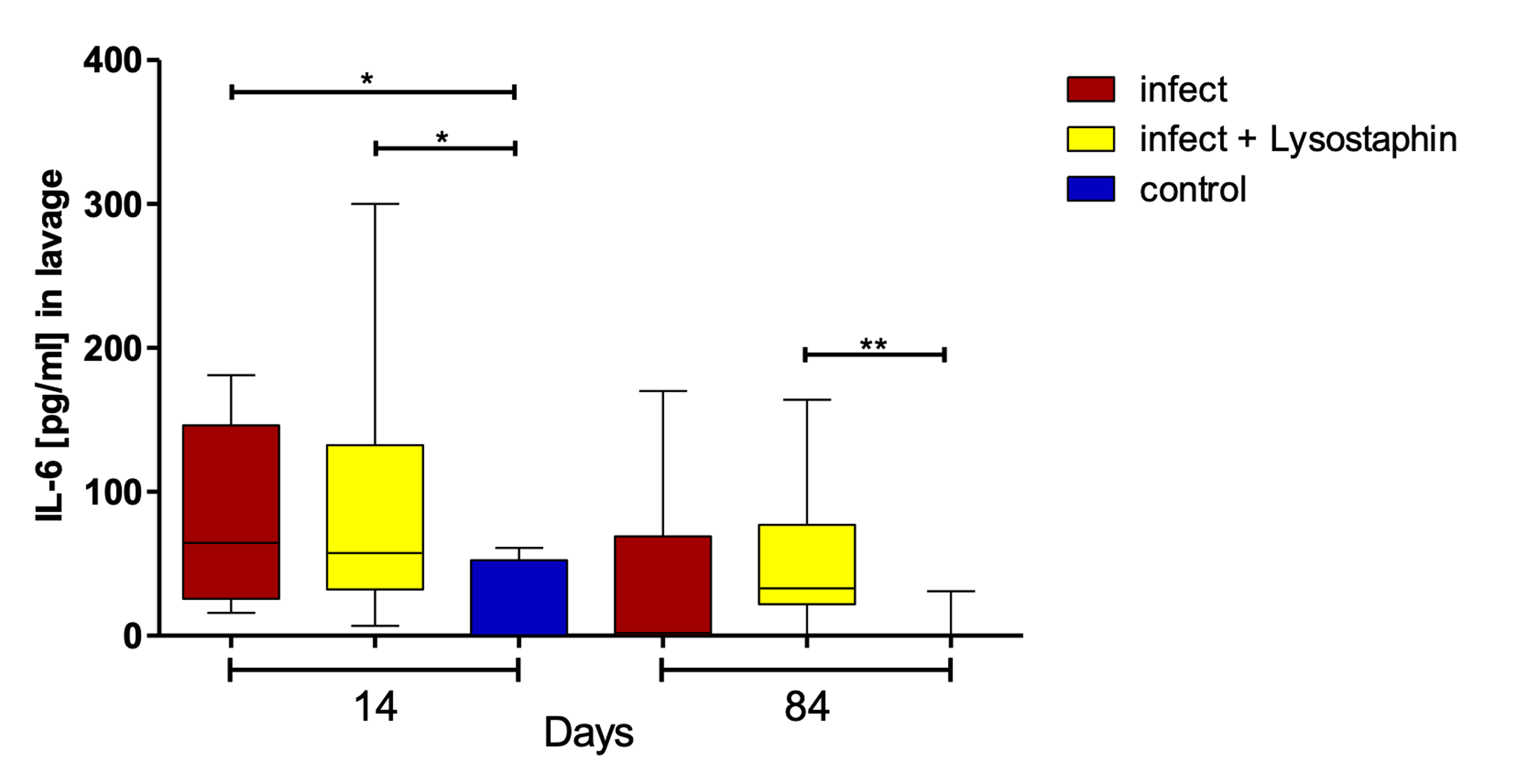

Supplement: Supplementary file 6 — (PNG 180 kb) [file 68_2024_2448_Fig10_ESM.png]

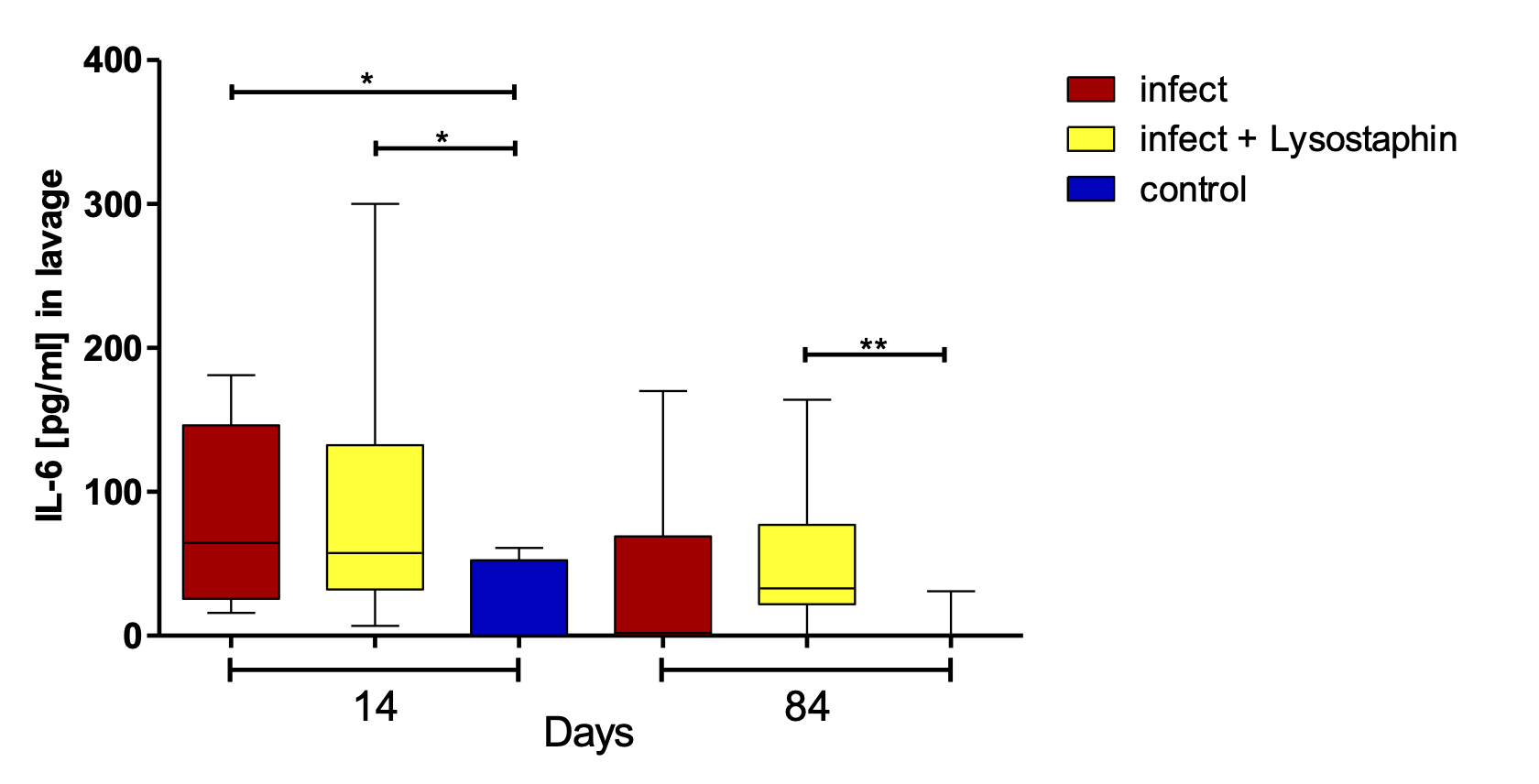

Supplement: Supplementary file 7 — High resolution image (TIF 85 kb) [file 68_2024_2448_MOESM4_ESM.tif]
